# Electronic Coupling of Molecular Complexes to Au Electrodes Mediated via Host–Guest Interactions

**DOI:** 10.1021/jacs.5c12957

**Published:** 2025-12-26

**Authors:** Isik Tuncay, Tzu-Chin Chang Chien, Florian Keller, Helena Roithmeyer, Laurent Sévery, Olivier Blacque, Marcella Iannuzzi, Murielle F. Delley, S. David Tilley

**Affiliations:** † Department of Chemistry, 27217 University of Zurich, Winterthurerstrasse 190, Zurich 8057, Switzerland; ‡ Department of Chemistry, 27209 University of Basel, Mattenstrasse 22, Basel 4002, Switzerland; § Laboratoire de Chimie et Biologie des Métaux, Université Grenoble Alpes, CNRS, CEA, IRIG, , 17 rue des Martyrs, Grenoble F-38054, Cedex, France

## Abstract

Immobilization of molecular catalysts onto electrode surfaces using host–guest (HG) interactions enables the facile regeneration of electrodes following catalyst degradation. Beyond this practical aspect, the architecture also offers a unique way to study the electronic coupling of molecules to the electrode through a nominally insulating linker. Here, we employ surface-enhanced infrared absorption spectroscopy (SEIRAS) to characterize the binding and electronic coupling of Au-bound HG complexes. Distinct spectral features and binding kinetics differentiate host-bound species from physisorbed analogues, confirming well-defined HG assemblies on the surface. Analysis of the wavenumber shifts of the guest as a function of applied potential suggests that while the coupling of physisorbed guests with the surface is not strong, the host-bound guests show a substantial electronic coupling considering their distance from the surface of ∼1.3 nm. Density functional theory calculations reveal the key role of the host in mediating this long-range coupling between the Au surface and the guest.

## Graphical abstract

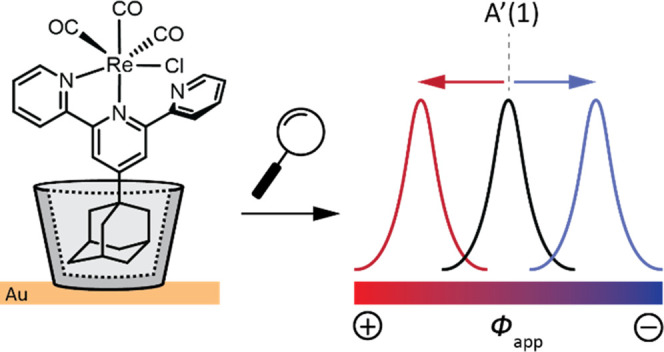

## Introduction

Electrocatalysts are critical to the development of sustainable energy and chemical processes. While molecular (electro)­catalysts often show higher activity and selectivity than heterogeneous catalysts, their applications are limited by durability, solubility, and challenging recovery. To overcome these limitations, extensive research has focused on the heterogenization of molecular catalysts.^1–10^ Heterogenization of molecular catalysts is typically achieved via direct anchoring, where the catalyst structure is modified with an anchoring group tailored to the chosen electrode material (Figure 1). Commonly, covalent bonds (e.g., addition of carboxylic or phosphonic acid groups for metal oxide substrates,^5,6,11^ thiols for gold substrates,^12–14^ and diazonium reduction for gold^15^ and carbon substrates^16^) and noncovalent interactions (e.g., π-interactions on carbon-based substrates^8,17–19^ ) are utilized for this purpose.

**Figure 1:**
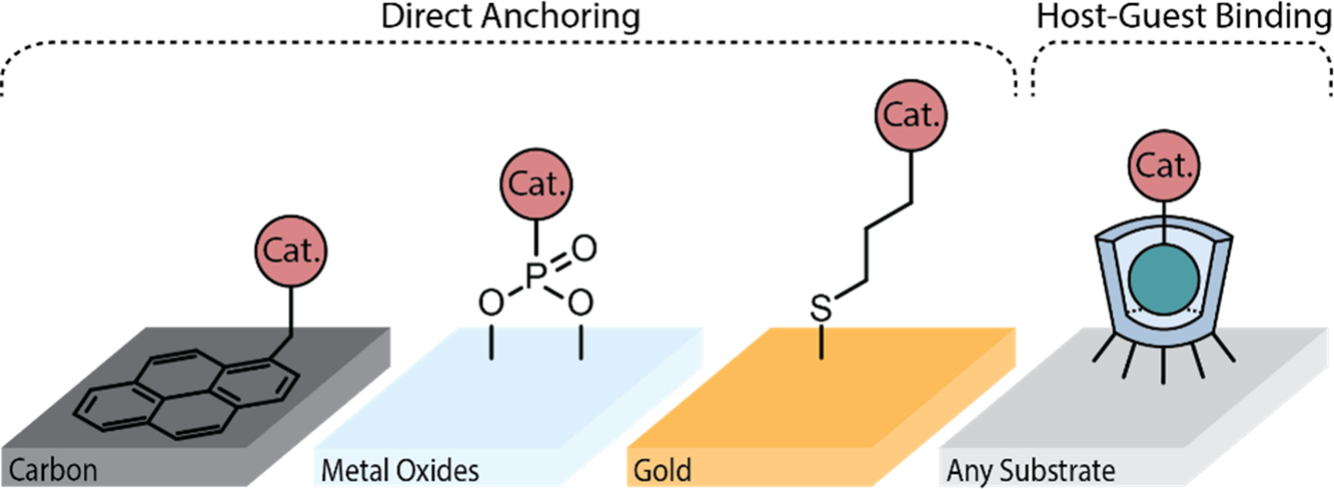
Schematic representation of immobilization of molecular catalysts on electrode surfaces via direct anchoring and host–guest complexation. In direct anchoring, the catalyst structure is altered with a binding unit that is suitable for the electrode material of interest. For host–guest complexation, the host has several of these anchoring groups that allows stable binding to the electrode surface and the guest is modified with a binding unit (blue sphere) that goes into the host cavity, and the same catalytic guest can therefore be used on different electrode materials.

Using host–guest (HG) interactions is another strategy for immobilizing molecular catalysts (Figure 1, right), where the host is a macrocyclic molecule and the guest is the molecular catalyst.^20–23^ In the HG strategy, the host is first covalently bound onto the electrode surface, followed by binding of the guest into its cavity. The molecular catalyst is modified with a binding unit that ensures stable HG complexation. Since HG interactions are typically driven by hydrophobic forces, the choice of binding unit depends only on its hydrophobicity and size,^24^ and catalyst modification is independent of the choice of the electrode material, allowing the same catalyst to be used across different electrode substrates. Furthermore, the macrocyclic structure of the host enables multivalent anchoring to the electrode surface, enhancing its stability compared to other anchoring strategies that use only one anchor. The stable attachment of the host to the electrode allows HG systems to withstand high pH conditions^21^ and enables the reutilization of the same electrode through guest exchange, either for the same target reaction or a different one.^22^

While immobilized molecular catalysts often exhibit redox-mediated activities, similar to their homogeneous counterparts, recent studies have shown that when a molecular catalyst is strongly coupled to the electrode surface, it can behave like a metal surface.^25^ In these cases, the catalysis is no longer redox-mediated, meaning the activity and selectivity of the catalyst are not constrained by its accessible oxidation states, allowing new reaction pathways.^25–27^ Such strong coupling behavior is generally observed when the catalyst is in close proximity to the surface so that it resides within the electrochemical double layer and, depending on the system, is generally lost when the catalyst is >0.6 nm away from the surface.^28^ In our previous study, we investigated HG complexes on Au surfaces using electrochemistry and tip-enhanced Raman spectroscopy (TERS).^20^ The absence of redox features of the guest in cyclic voltammetry (CV) measurements suggested strong coupling between the host-bound guests and the Au surface, and the observed change in the molecular HOMO–LUMO gap that was observed by TERS measurements supported this. However, the strong background oxidation from the Au surface made the electrochemical analysis of the system challenging; hence, the strength of this interaction was uncertain. We therefore wished to investigate this coupling by using a technique that would not be affected by the unwanted background activity.

Surface-sensitive in situ vibrational spectroscopic techniques, such as surface-enhanced infrared absorption spectroscopy (SEIRAS) and surface-enhanced Raman spectroscopy (SERS), are potent tools that allow characterization of submonolayers that are attached to a surface.^29,30^ Moreover, they can be coupled with electrochemistry to study shifts in the vibrational modes as a function of the applied potential. These shifts have been intensively studied to probe the electrochemical interfaces and to measure the electric field experienced by surface-bound molecules.^31–40^ While such shifts are mainly interpreted in terms of the effect of the generated electric field (i.e., vibrational Stark effect), the orbital overlap between the surface and the adsorbate can also contribute to these shifts (i.e., chemical bonding effect).^41–45^ Previous work carried out using molecules that consist of two vibrational probes (e.g., 4-isocyanobenzonitrile and 1,4-diisocyanobenzene) shows that the vibrational probe that is directly attached to the surface generally demonstrates a larger potential-induced shift compared to the free vibrational mode.^38,46^ Furthermore, such systems can help distinguish between the contributions from electric field and chemical bond effects, enabling a quantitative assessment of how strongly the molecule binds to the surface.^47^ These studies highlight that the magnitude of the potential-induced vibrational shifts provides valuable insight into the interaction strength between surface-bound molecules and the surface.

Here, we employed SEIRAS to study in detail the interaction between the host-bound guests and the Au surface through the investigation of the potential induced shifts in the guest vibrational modes. In addition, we observe the binding of the host to Au surfaces, HG complexation, and physisorption in real time. The difference between the HG and physisorption SEIRA spectra provides further evidence of the formation of HG complexes on the host-functionalized Au electrodes. The clear observation of a potential-induced vibrational shift for the HG complexes at ∼1.3 nm away from the surface in high-ionic-strength media suggests that the host-bound guests are electronically coupled to the surface. The host-bound guests showed similar (within error) vibrational shifts with applied potential as physisorbed guests, despite being significantly farther away from the electrode, in line with a stronger electronic coupling to the surface with HG complexes compared to physisorbed species. Although the projected density of states (PDOS) analysis indicates that the electronic structure of the host-bound guests remains unaffected by the Au surface, the host interacts with both the guest and the surface, suggesting that the host facilitates electronic communication between them, despite the use of a nominally insulating adamantane moiety as the guest binding unit.

## Results and Discussion

### Investigation of the Host Binding

A per-thiolated beta-cyclodextrin (beta-CD, **1**) was chosen as the host, where the host immobilization occurs via the seven sulfur atoms, ensuring stable binding to the gold surface (Figure 2).^48^ We attempted to monitor the binding of host **1** via the loss of the S–H vibrational mode ( ${ṽ}_{SH}$ ), but we were unable to detect the ${ṽ}_{SH}$ band, which could be due to the fast reaction between the thiol groups and the Au surface or due to the weak intensity of this band. To have stronger IR signals from the host that would not be affected by the surface attachment, we synthesized host **2**, in which the secondary hydroxyl groups were acetylated. Both SEIRAS and quartz crystal microbalance with dissipation monitoring (QCM-D) measurements indicated that the binding of the host to the Au electrode is complete within 5 min (Figures S4a and S5). From the QCM-D measurements, the surface density of host **2** was found to be ∼0.5 nm^–2^, suggesting a densely packed host layer on Au, consistent with our previous results.^20^ NMR titration studies revealed that the acetyl groups of host **2** hinder HG complexation (Figure S7). The guest binding studies described in the following sections were, therefore, carried out with host **1**.

**Figure 2:**
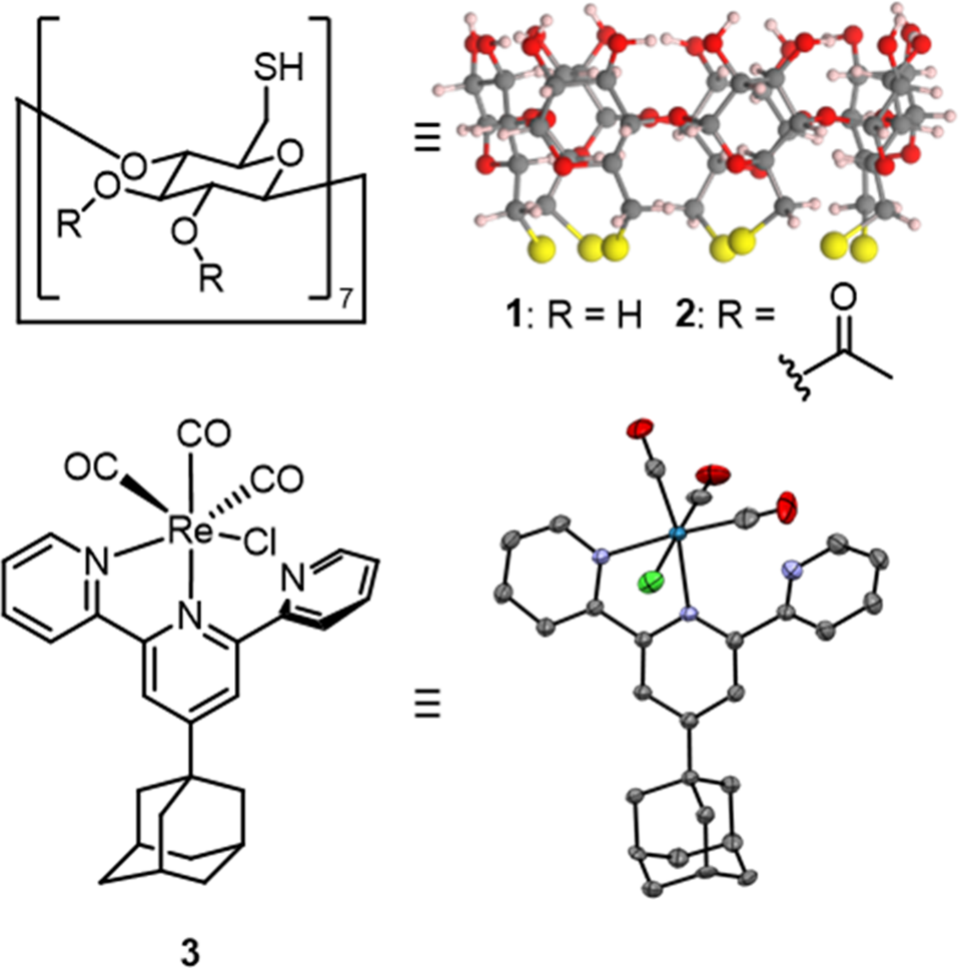
Chemical structures of (**1**) per-thiolated β-CD, along with its calculated structure, (**2**) the acetylated version of the same host, and (**3**) the rhenium tricarbonyl complex used as a guest in this study. The crystal structure of the guest is shown at a 30% probability, and the hydrogen atoms and solvent are omitted for clarity.

### Guest Molecule Design and Characterization

For the guest, [Re­(4′-(adamantan-1-yl)-2,2′:6′,2″-terpyridine) (CO)_3_Cl] complex **3** was used. Rhenium tricarbonyl complexes have been extensively studied and are known to exhibit strong carbonyl stretching modes in IR that are sensitive to the changes in the chemical environment.^49–51^ The terpyridine (terpy) ligand was selected based on our previously published catalytic HG systems.^20,21^ HG complexation is driven by the adamantane unit, which is known for its high binding affinity to the β-CD cavity.^52^ The bidentate coordination of the terpy ligand to the Re metal center was confirmed by ^1^H NMR (Figure S1a) and X-ray crystal structure analysis (Figure 2).^53,54^ The FT-IR absorption spectra of guest **3** in DMF (Figure S8) showed three tricarbonyl stretching modes ( ${ṽ}_{CO}$ ), which is typical for the fac- arrangement. The band around 2018 cm^–1^ is assigned to the symmetric stretching mode, A′(1), and the two bands around 1914 cm^–1^ and 1891 cm^–1^ are assigned to the two asymmetric stretching modes, A″ and A′(2), respectively, aligning well with previously reported values.^50^

### Guest Immobilization on Au via HG Complexation versus Physisorption

The formation of surface-bound HG complexes was investigated by recording SEIRA spectra during the exposure of a host **1**-functionalized Au surface to a methanolic solution of guest **3** (Figure S9). Physisorption measurements were taken by using an unmodified clean Au surface (Figure S11). Afterward, a MeOH wash was done to remove weakly bound guests (Figures S10–S12). Figure 3a shows the corresponding SEIRA spectra after 1 h of guest immersion, followed by a MeOH wash.

**Figure 3:**
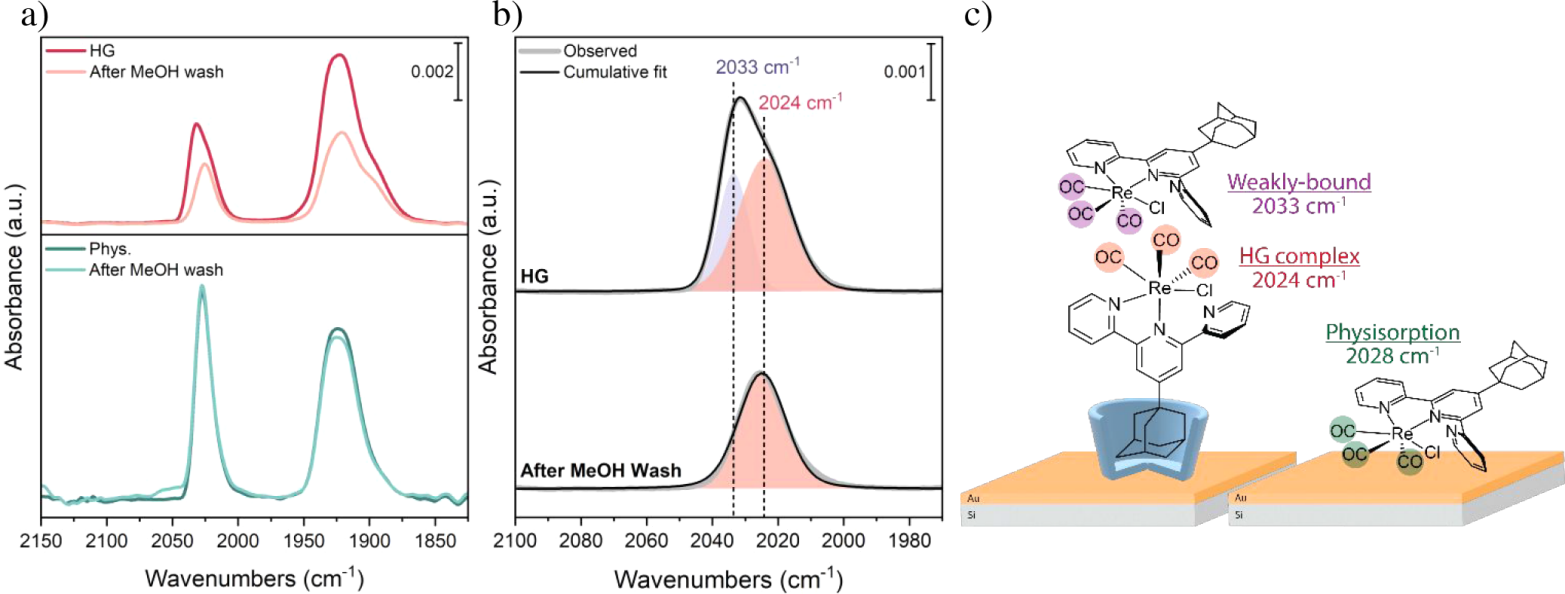
Detection of surface immobilization of guest **3** by SEIRAS in MeOH solution. (a) SEIRA spectra of the HG complexes (top) and physisorption (bottom). Light-colored lines show the spectra after MeOH wash in the respective plots. (b) Voigt fit of the A′(1) band of the HG spectra before (top) and after MeOH wash (bottom). (c) Schematic representation of the guests in different environments.

At least three ${ṽ}_{CO}$ bands were observed in both cases. Due to the less defined nature of the asymmetric stretching modes, we focused our analysis on the symmetric stretching mode, A′(1). From the FT-IR measurement of guest **3** in DMF, a single band above 2000 cm^–1^ is expected for the A′(1) mode. While physisorption SEIRA spectra showed a single A′(1) band around 2028 cm^–1^ (Figure 3a, dark-green line), a Voigt fit revealed the presence of two bands in the HG case (Figure 3b, top), one around 2033 cm^–1^ and another around 2024 cm^–1^. After the MeOH wash, the band around 2033 cm^–1^ disappeared (Figure 3b, bottom), whereas in the physisorption case, no significant spectral changes were observed (Figure 3a, light-green line). To further characterize the observed bands in the HG case, the integrated band areas, which depend on relative surface populations among other factors,^55^ were plotted against immersion time (Figure S13). The integrated band area for the band around 2024 cm^–1^ shows growth, as expected for a binding event, whereas the one around 2033 cm^–1^ shows almost no change. This suggests that the band around 2024 cm^–1^ corresponds to the HG complexation, whereas the one around 2033 cm^–1^ corresponds to weakly bound guests located outside of the host cavity or guests in the bulk solution. The IR spectrum of guest **3** in MeOH recorded without the Au layer displayed no ${ṽ}_{CO}$ bands at concentrations of 0.1 mM, 0.5 mM, and 1 mM. This suggests that the concentration of guest **3** used for SEIRAS measurements is below the detection limit of the ATR setup for the detection of bulk solution species. Moreover, the lack of a secondary band in the physisorption case further supports that the band around 2033 cm^–1^ corresponds to weakly bound guests that are outside the host cavity.

Solution-phase FT-IR spectra of guest **3** with or without 1 equiv of β-CD displayed no differences in the ${ṽ}_{CO}$ band positions and shape (Figure S14a), suggesting that the shift of the A′(1) band between weakly bound guests and HG complexes arises from an interaction between guest **3** and the Au surface and not due to HG interactions. Paoprasert et al. showed that the magnitude of the blue shift upon immobilization of a Re­(CO)_3_ complex on TiO_2_ depended on the interaction strength between the molecule and the surface, which weakened as the distance from the surface increased.^56^ Therefore, considering that β-CD has a height of 0.79 nm,^57^ we would expect the HG complexes to have a weaker interaction with the Au surface, compared to physisorption. To examine this, the solution FT-IR spectrum of guest **3** in MeOH was measured and the band around 2035 cm^–1^ was assigned as the A′(1) band of guest **3** in solution (Figures S15–S17). Upon immobilization of guest **3** on the Au surface, we observe a red shift for all the detected A′(1) bands in the SEIRAS measurements. The observed red shift in all cases can be explained by a partial charge transfer from the e^–^-rich Au to the Re center, hence weakening the CO bond. The magnitude of the red shift follows the order of HG (2023 cm^–1^) > physisorption (2029 cm^–1^) > weakly bound guests (2033 cm^–1^), obtained from the average A′(1) band position values across several independent replicate experiments (Figure S18c). In both HG and physisorption systems, similar solvent features were observed (Figures S19–S23), which suggests that the red shifts originate from an electronic interaction with the Au layer. Notably, the observed trend suggests that the host-bound guests are electronically more strongly coupled to the Au surface than the physisorbed guests, despite the larger distance from the surface. While the exact mechanism of this coupling is uncertain, the distance and the noncovalent attachment suggest a through-space coupling effect, similar to those observed in supramolecular junction systems that consist of σ–σ stacking or HG interactions.^58,59^

The comparison of SEIRA spectra of the HG complexes to physisorption also reveals that the A′(1) band appears broader and less intense for the HG case (Figure S18a,b). While quantitative comparison of band intensity from experiment to experiment is challenging due to the use of a fresh Au layer in each experiment, the reproducibility from experiment to experiment in the obtained band intensities suggests that a qualitative analysis is still possible (Figures S9–S12 and S18). We expect lower integrated band areas for HG compared to the physisorbed species due to (i) lower surface coverages in the HG case because of large host molecules and the known formation of a 1:1 HG complex of adamantane with β-CD, (ii) the possible formation of multilayers through π-interactions in the physisorption case, (iii) the larger distance from the surface in the HG case leading to lower surface enhancement factors in SEIRAS,^60^ and (iv) possible orientation differences between the two binding modes that would impact the band intensity according to surface selection rules.^55,60–62^ Dipole–dipole coupling effects as a function of surface coverage can also influence band intensities and vibrational shifts.^63,64^ However, variation of the concentration of guest in solution over 3 orders of magnitude in different physisorption experiments gave A′(1) bands with similar band intensity and vibrational shift, suggesting that dipole–dipole or surface coverage effects do not significantly influence our observations (Figure S24 and Table S4). Even though it is challenging to quantify how much each of these factors contributes, the observed differences indicate that we can clearly distinguish between the two different binding modes (HG complexation vs physisorption). This is also highlighted by their differences in binding kinetics, where the rate for HG complexation was on average ∼3 times slower than that of physisorption (Figure S25), likely due to the more restricted range of orientations that guests must have in order to enter the host cavity. Moreover, the lack of a second A′(1) band in the HG SEIRA spectra after MeOH wash suggests that when the surface is passivated with the host molecules, the only stable binding mechanism for the guests is via HG complexation.

### DFT Calculations

DFT calculations were utilized to investigate the adsorption selectivity of guest **3** on Au(111) through various physisorption and HG complexation geometries (see Supporting Information for further details). The two physisorption configurations, with either the trans –CO ligand or the –Cl ligand facing the Au, showed similar adsorption energies in both configurations, with PS2 being slightly more stable (Figure 4a,b). This is in agreement with the prior work done by Clark and co-workers.^65^ However, they also show that in the experimental case, the configuration where the –CO ligand is facing the Au surface is more present, which may also be the case here.

**Figure 4:**
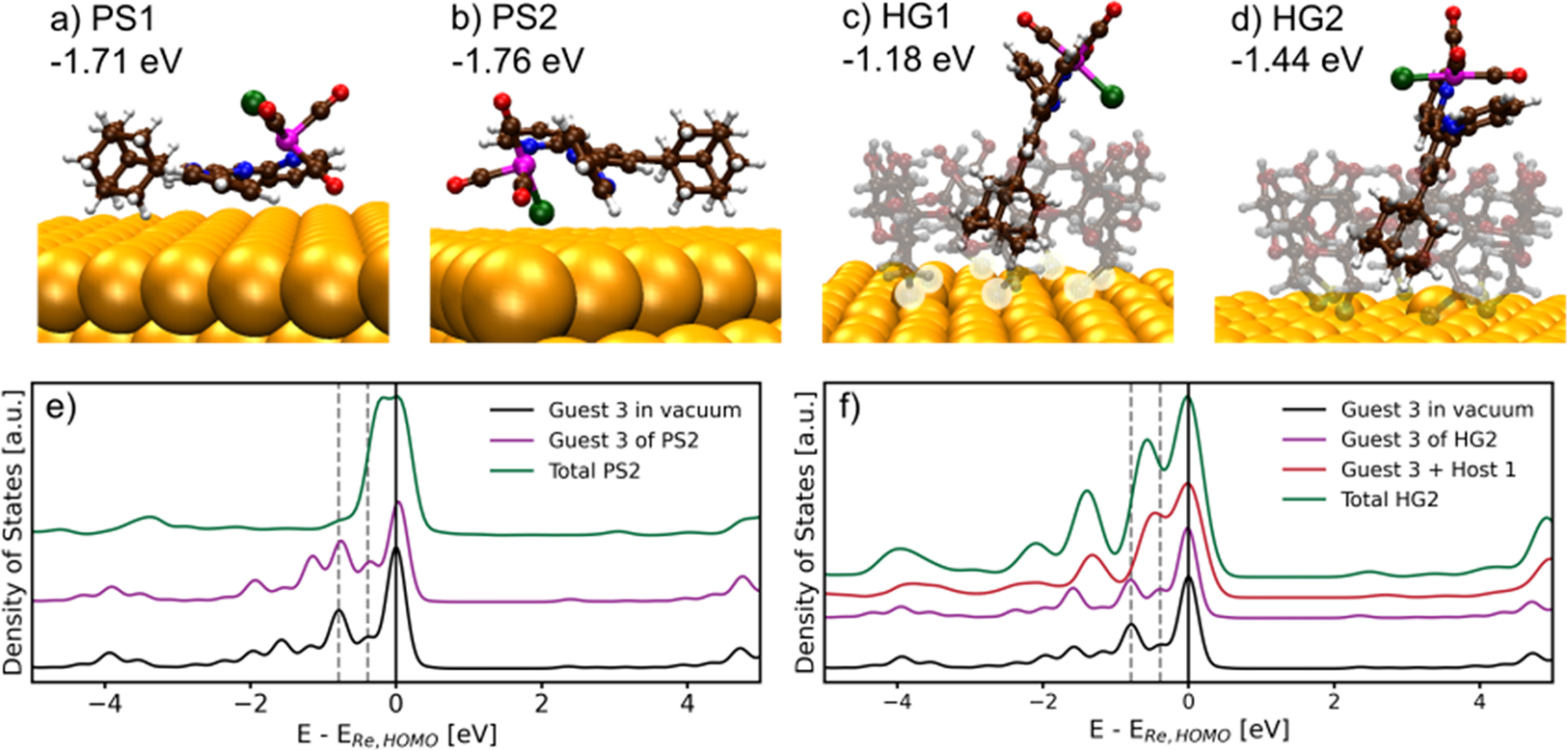
Energetically most favored configurations for physisorption, (a) PS1 and (b) PS2, and HG complexes, (c) HG1 and (d) HG2 with their respective adsorption energies. Color code for the atoms: Au = gold, C = brown, H = white, N = blue, Re = pink, O = red, Cl = green. (e) PDOS of Re states for the optimized guest **3** structure in vacuum (black line), in PS2 configuration without (purple line), and with (green line) the Au(111) surface. (f) PDOS of Re states for the optimized guest **3** configuration in vacuum (black line), in HG2 configuration without the host and Au(111) surface (purple line), and HG complex in HG2 configuration without (red line) and with (green line) the Au(111) surface. The gray dashed lines indicate the two states below the HOMO level of the optimized guest **3** in vacuum. A more detailed analysis of the PDOS is provided in the Supporting Information.

The two most stable HG configurations, HG1 and HG2, show that complexation occurs via inclusion of the adamantane unit in the host **1** cavity, resulting in a desirable orientation for a catalytic system by exposing the catalytic site to the electrolyte (Figure 4c,d). The stronger binding observed in HG2 compared to that in HG1 is attributed to the tilted orientation of guest **3** in HG1, which introduces strain in both host **1** and guest **3**, thereby reducing the interaction energy (Figure S27).

We then calculated the PDOS for the most stable HG and physisorption configurations to investigate the electronic interactions between guest **3** and the Au(111) surface (Figures S31–S34). Upon adsorption onto Au(111), the Re states of the physisorbed guest **3** exhibited pronounced changes, indicating electronic interaction with the surface (Figure 4e). However, in contrast to our previous study,^20^ the Re states of the host-bound guests remained largely unaffected upon adsorption of the HG complex onto Au (Figure 4f). This discrepancy is likely due to the different binding units of the guests, with the adamantane unit not being able to approach the Au surface as closely as the naphthyl binding unit in host **1** cavity. Nevertheless, in Figure 4f, we observe that the Re states change upon the formation of HG complexes in the absence of the Au layer, indicating an electronic coupling between the host and the guest. When the same HG configuration is adsorbed on Au, no further notable changes in the Re states were observed, showing that host-bound guest **3** is not affected by the Au surface directly. In Figure S34d, the appearance of new host **1** states upon HG binding on the Au surface was observed, which arises from the formation of seven S–Au bonds. Hence, we reason that as guest **3** is coupled with host **1** (evidenced by the changes in Re states upon HG complexation) and host **1** is coupled with the Au surface (evidenced by the new states upon adsorption on Au), host **1** can mediate electronic communication between guest **3** and the Au. To further investigate this, IR spectra of isolated guest **3** and guest **3** adsorbed on Au in PS1, PS2, HG1, and HG2 configurations were calculated (Figures S28 and S29 and Tables S5 and S6). A shift in the IR band positions was indeed observed upon complexation to the host-modified Au surface, indicating a modification of the electronic structure at the Re site. However, these calculations did not quantitatively match the experimental findings, likely due to a lack of thermal and dynamic effects in the DFT calculations.

### A′(1) Band Shifts as a Function of Applied Potential

The dependence of the vibrational band position on the applied potential can provide insight into the strength of the adsorbate–surface interaction. The Au surface with guest **3** was studied via either physisorption or HG complexation. Acetonitrile, which can dissolve the guest, was selected as the solvent for the electrochemical measurements to investigate possible stability differences between the two binding modes under applied potential (i.e., if the guests are unstable under applied potential, they will desorb from the surface, and we would observe a loss of signal). After the surface functionalization with guest **3**, 0.2 M TBAPF_6_ in MeCN was placed into the cell, and SEIRA spectra were recorded during a series of chronoamperometry (CA) measurements with 50 mV steps. The A′(1) band was then fitted with a Voigt function to obtain the band position as a function of applied potential (Figure 5). Before each CA series, CV curves were measured to ensure that no faradaic processes that could affect the shifts in ${ṽ}_{CO}$ were observed (Figure S35). The electrochemical inertness of guest **3** within the selected potential window is further supported by the absence of spectral features that are associated with 1 e^–^ redox products of similar Re complexes (Figure S36).^66,67^

**Figure 5:**
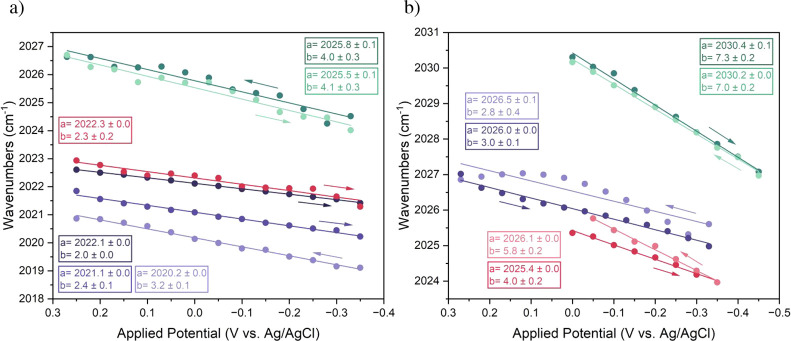
Plot of the A′(1) band position as a function of the applied potential for (a) HG and (b) physisorption. Different colors correspond to different repetitions. Arrows show the direction of the applied potential sweep. Linear fits of the A′(1) band position vs applied potential with intercept a and slope b for each repetition are shown in color-coded insets.

In each repetition, we measured both negative and positive directions of applied potential to ensure that the shifts were reversible as the direction of applied potential changed (Figures 5 and S37–S40). A positive applied potential direction resulted in blue shifts, while reversing the direction led to red shifts. This is generally consistent with what has been previously observed for similar anchored Re tricarbonyl complexes.^65^ Notably, as the direction of the applied potential was reversed, the response of the physisorbed guests showed more variability (Figure 5b) compared to that of HG complexes. This is likely due to the less controlled binding in the physisorption case, enabling guests to reorient more as the direction of the applied potential changes, hence leading to hysteresis.

To assess how strongly the guests experience the applied potential, we compared the slopes obtained from linear fits (insets in Figure 5). The different potential ranges in physisorption repetitions were due to the instability of the Au surface, which prevented the completion of some experiments. On average, the obtained slope values were 3.0 ± 0.9 cm^–1^/V for HG complexes and 5 ± 2 cm^–1^/V for physisorption, where the errors correspond to the standard deviation (σ). The σ values indicate that HG complexes showed less variability within different repetitions, which could be due to a more controlled orientation and surface binding, resulting in improved reproducibility. Prior work suggests that vibrational slopes with applied potential can be a function of both through-space electrostatic effects and electronic coupling with the surface.^41,42,44,45,47^ These contributions cannot, in general, be disentangled. However, some information may be obtained by comparison to literature reports of related systems. The slope values obtained herein are relatively small compared to slopes obtained for CO directly bound to metal surfaces (∼30–60 cm^–1^/V),^31,68–70^ and the slope obtained with a Re–CO complex anchored to Au via a thiol linker (21 cm^–1^/V).^65^ The latter system might be particularly suited to compare with our physisorbed data due to the similarity in the nature of the system, the proximity, and orientation with respect to the gold surface. The DFT calculations show that the distance of the Re center to the Au surface is between 0.4 and 0.5 nm for physisorption, while this distance is increased to 1.2–1.3 nm for the HG complex (Figure S30). The smaller slopes obtained in our case compared to the previously reported system may hence be due to differences in electronic coupling with the surface, electrolyte strength and identity, or interfacial structure, leading to differences in experienced potential drop. A smaller electronic coupling with the surface would be expected for physisorbed versus the reported thiol-linked Re complexes, in line with the observed smaller vibrational slopes. A comparison between the physisorbed and HG systems is made difficult by possible differences in interfacial structure, orientation, and packing influencing the potential screening through electrolyte components (Figure 6). In a simplified view, a smaller slope with the HG system compared to the physisorbed species was expected, considering the larger distance from the surface in the former case. Our data does indeed imply smaller average slopes for the HG system, though within statistical uncertainty of the slopes obtained with the physisorbed guests. While the orientation of the physisorbed guests on Au likely results in a reduced sensitivity to the electric field normal to the surface, the observation of a clear slope for the HG system at 1.2–1.3 nm from the surface is notable. In a 0.2 M TBAPF_6_ electrolyte, the field may already be largely screened at the HG complex. This implies that the HG complex experiences a considerable electronic coupling to the surface, leading to the observed shift. To further investigate this, a repetition experiment of the HG complexes at a 1 M TBAPF_6_ concentration was conducted (Figure S41). Upon increasing the concentration from 0.2 to 1 M TBAPF_6_ in MeCN, the estimated Debye length decreases from ∼0.5 to ∼0.2 nm at room temperature. Assuming that the diffuse layer begins at the outer rim of the host (∼0.8 nm) due to the inability of the large TBA^+^ ions to penetrate into the tightly packed host layer (Figure 6a), the potential is likely completely screened before it reaches the Re center of the guest **3** at a 1 M TBAPF_6_ electrolyte concentration. Nevertheless, the observed slope values (Figure S42) were similar to those at 0.2 M TBAPF_6_, suggesting that electronic coupling plays a more dominant role than electric field effects in the HG system. This is consistent with prior work by Bhattacharyya and co-workers that attributes an observed slope of 1.4 cm^–1^/V for an anchored W–CO complex at a similar distance from the surface to large Stark tuning rates.^36^ A considerable electronic coupling in the case of the HG system is further in line with the observed red shifts of the A′(1) modes upon surface binding in HG and physisorbed systems. In sum, the observed vibrational slopes with applied potential likely arise from both through-space effects and electronic coupling, which present a considerable contribution, especially in the case of the HG system.

**Figure 6:**
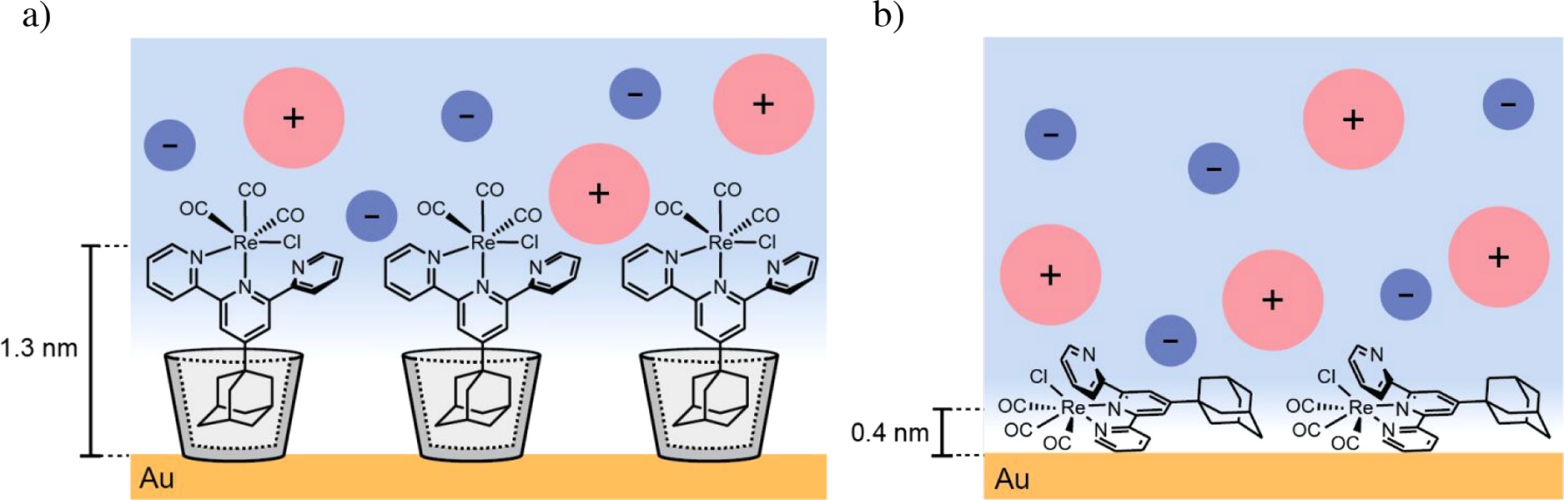
Schematic representation of (a) HG and (b) physisorption on the Au surface showing the differences in surface orientation, distance of the Re center from the Au surface (insets), and ion penetration in both systems. Due to the tight packing of the host **1** layer, we assume that the ions can approach the electrode only up to the upper rim of host **1**.

While PDOS analysis showed no direct electronic interaction between the host-bound guests and the Au surface, the experimental evidence of such coupling suggests that the host mediates an electronic communication between the guest and the Au surface. This coupling may resemble the electrostatic coupling observed between a physisorbed Co molecular catalyst and graphene surface, as demonstrated by Hutchison and co-workers.^28^ Their work shows that, although the electronic structure of the catalyst is unaffected by the graphene surface, the hydrophobic nature of both the catalyst and the surface leads to cosolvation if the distance between them is small enough to prevent solvent penetration (∼0.3 nm). While this might suggest that the HG system enables cosolvation at greater distances from the surface, the closer proximity of the physisorbed guest to Au suggests that cosolvation alone may not fully account for the stronger coupling observed in the HG system. Therefore, it is plausible that the host mediates electronic communication in a manner analogous to molecular junction systems that consist of noncovalent interactions, where a through-space charge transport mechanism is observed.^58,59,71,72^

In a recent study, Abdinejad and co-workers demonstrated that strong coupling between a molecular catalyst and an electrode surface can change the selectivity of a molecular catalyst.^27^ The electrocatalytic performance of the HG system needs to be investigated to see whether the stronger coupling in this case is sufficient to similarly influence its reactivity. Although Re­(CO)_3_ complexes are known to be active for electrocatalytic CO_2_ reduction, we were unable to study this due to the reductive desorption of host **1** from the Au surface at highly reductive conditions. The influence of this coupling on catalytic selectivity will be addressed in future studies.

## Conclusions

By employing SEIRAS, we were able to provide further evidence for the formation of HG complexes on Au surfaces, while direct observation with techniques such as STM remains challenging. In situ SEIRAS and QCM-D measurements revealed the rapid formation of a densely packed host layer within 5 min. Comparative analysis of the A′(1) vibrational mode for host-bound and physisorbed guests revealed differences in band locations and integrated areas, reflecting the distinct environment in which the guests reside in these two systems. The observed red shifts in the guest A′(1) band upon HG complexation on the Au surface, along with the potential-induced vibrational shifts, indicate that the host-bound guests are substantially coupled to the Au surface, despite the considerable distance. DFT calculations indicate that this long-range coupling is mediated by the host, analogous to supramolecular junction systems. These findings reveal favorable implications of the HG strategy for electrocatalytic applications: the controlled orientation enhances the reproducibility and ensures that the catalytic site is facing the bulk solution, while electronic coupling to the surface may enable new reactivity pathways. Together with the ability to reuse the same host-modified electrode surface for different target reactions, the host-mediated strong coupling suggests that the HG system could provide a flexible platform to harness strong coupling between molecular catalysts and the electrode surface across various reactions.

## Supplementary Material



## Notes

Additional experimental details, spectroscopic data, synthesis and characterization of compounds (PDF)
