# Supplementary material for: Electronic Coupling of Molecular Complexes to Au Electrodes Mediated via Host–Guest Interactions
Source: J Am Chem Soc. 2025 Dec 26;148(1):330–9. doi: 10.1021/jacs.5c12957 (PMC12814342; doi:10.1021/jacs.5c12957)
Supplement: Supplementary file 1 [file ja5c12957_si_001.pdf]

Supporting Information

Electronic Coupling of Molecular Complexes to Au electrodes Mediated via Host-Guest Interactions

Isik Tuncay[1], Tzu-Chin Chang Chien[2], Florian Keller[1], Helena Roithmeyer[1], Laurent Séverу[3], Olivier Blacque[1], Marcella Iannuzzi[1], Murielle F. Delley[2] *, S. David Tilley[1] *

[1]Department of Chemistry, University of Zurich, Winterthurerstrasse 190, 8057 Zurich, Switzerland

[2]Department of Chemistry, University of Basel, Mattenstrasse 22, 4002 Basel, Switzerland

[3]Université Grenoble Alpes, CNRS, CEA, IRIG, Laboratoire de Chimie et Biologie des Métaux, 17 rue des Martyrs, F-38054 Grenoble, Cedex, France

Corresponding author email: murielle.delley@unibas.ch & david.tilley@chem.uzh.ch

Table of Contents

General information & instrumentation **4**

NMR spectroscopy 4

Mass spectrometry 4

X-ray crystallography 4

FT-IR spectroscopy 4

Quartz crystal microbalance with dissipation monitoring (QCM-D) 4

Surface enhanced infrared absorption spectroscopy (SEIRAS) 5

Electrochemical measurements 5

Computational deatils 5

Synthesis and characterization **6**

Procedures for guest adsorption SEIRAS measurements **12**

SEIRA spectra during host 2 adsorption **12**

QCM-D measurements of host 2 .... **13**

NMR titration studies **13**

FT-IR characterization of guest 3 .... **17**

SEIRAS replicate measurements of HG complexation **18**

SEIRAS replicate measurements of MeOH wash after HG complexation **19**

SEIRAS replicate measurements of physisorption **20**

SEIRAS replicate measurements of MeOH wash after physisorption **21**

Data fitting of the SEIRA spectra **21**

SEIRAS HG band growth overtime **22**

FT-IR measurement of solution phase HG complex **23**

FT-IR measurements of guest 3 in MeOH and band assignments **23**

A'(1) band comparisons **26**

Analysis of solvent features **27**

Physisorption A'(1) band comparison with varying guest 3 solution concentration ... **32**

Binding kinetics analysis **32**

Adsorption selectivity of guest 3 on Au(111) **34**

Adsorption selectivity of guest 3 in and around host 1 .... **35**

Vibrational spectra of guest 3 .... **36**

Distance analysis of guest 3 for most stable configurations of physisorption & HG ... **42**

Projected density of state (PDOS) analysis **43**

PS1 44
PS2 45
HG1 46
HG2 48
Cyclic voltammograms of Au with adsorbed guest 3 49
Examples of SEIRA spectra showing the vCO bands during the applied potential 49
SEIRAS replicate measurements of HG complexes as a function of applied potential 50
SEIRAS replicate measurements of HG complexes as a function of applied potential (reverse scans) 51
SEIRAS replicate measurements of physisorption as a function of applied potential 52
SEIRAS replicate measurements of physisorption as a function of applied potential (reverse scans) 53
SEIRAS measurement of HG complexes as a function of applied potential at 1 M $TBAPF_6$ concentration 54
A'(1) band shifts as a function of applied potential for the HG complex at 1 M $TBAPF_6$ concentration 55
References 56

General information & instrumentation

All reagents and solvents were purchased from Merck/Sigma Aldrich or Chemie Brunschwig, except for **1** (6-per-thio-β-CD) (≥ 97%, Cyclodextrin-Shop, Netherlands). The 4'-(adamantan-1-yl)-2,2':6',2"-terpyridine was synthesized and characterized as published[1]. All solvents used were analytical grade.

NMR spectroscopy

All spectra were recorded with a 400 or 500 MHz Bruker Avance spectrometer. All spectra were referenced according to their residual solvent signals and processed with Mnova.

Mass spectrometry

All mass spectra were recorded using a Thermo DFS high-resolution mass analyzer (GC-)MS equipped with electron- and chemical-ionization (EI and CI), direct chemical ionization (DCI), gas chromatograph (GC), and autosampler incl. headspace sampling and automatic derivatization.

X-ray crystallography

Single crystal X-ray diffraction data were collected at 160.0(1) K on a Rigaku OD Synergy/Hypix diffractometer using the copper X-ray radiation (λ= 1.54184 Å) from a dual wavelength X-ray source and an Oxford Instruments Cryojet XL cooler. The selected suitable single crystal was mounted using polybutene oil on a flexible loop fixed on a goniometer head and immediately transferred to the diffractometer. Pre-experiment, data collection, data reduction and analytical absorption correction[2] were performed with the program suite *CrysAlisPro*.[3] Using *Olex2*,[4] the structure was solved with the *SHELXT*[5] small molecule structure solution program and refined with the *SHELXL* program package[6] by full-matrix least-squares minimization on F^2. *PLATON*[7] was used to validate the result of the X-ray analysis. The adamantane ligand is disordered over two sets of positions with site-occupancy factors of 0.465(4) and 0.535(4). Similarity restraints (*SADI* instruction in *SHELXL*) were applied to the C-C bond lengths in the disordered ligand. All H-atoms were placed geometrically and refined isotropically using a riding model, with C—H = 0.95 Å (C-aromatic), 0.99 Å (C-methylene) and 1.00 Å (C-methine), in association with U_{iso}(H) = 1.2U_{eq}(C). CCDC- 2466002 (**3**) contains the supplementary crystallographic data for this paper. The data can be obtained free of charge from The Cambridge Crystallographic Data Centre via www.ccdc.cam.ac.uk/structures.

FT-IR spectroscopy

FT-IR spectra were recorded on a Bruker Tensor 27 spectrometer equipped with a single element HgCdTe detector, using a home-made air-tight flow cell consisting of two CaF_2 windows (2 mm thickness) separated by a 50 µm PTFE spacer.

Quartz crystal microbalance with dissipation monitoring (QCM-D)

QCM-D measurements were done by using a QSense Explorer (Biolin Scientific) module with 5 MHz AT-cut 14 mm diameter Cr/Au coated quartz sensors (QSX 301, Biolin Scientific). The sensors were cleaned by UV/ozone treatment for 20 minutes and rinsed with EtOH then dried under N_2 flow. All measurements were performed at a set temperature of 22°C and a flow rate of 0.1 mL/min. Before each measurement, MeOH was drawn through the cell until a stable baseline

was reached. The monitored frequency and dissipation shifts were analyzed using QSense DFind software. The surface density (Δm) was calculated using the Saurbrey equation[8]:

$$\Delta m = -\frac{C * \Delta f}{n} \tag{1}$$

where Δf is the change in the frequency, C is the sensitivity constant (17.7 ng cm^{-2} Hz^{-1} for 5 MHz crystal) and n is the number of the overtone. For all measurements, 7th overtone was used for Δm calculations.

Surface enhanced infrared absorption spectroscopy (SEIRAS)

SEIRA spectra were measured in a commercial three electrode spectrochemical cell (Jackfish SEC) with a Si face-angled crystal (FAC) mounted on a VeeMAX III ATR accessory (PIKE Technologies). A Bruker VERTEX 70v or VERTEX 80 Fourier Transform Spectrometer equipped with a photovoltaic liquid N_2 cooled MCT detector and purged with dry air was used for all infrared spectroscopic measurement. The adjusted angle of incident (AOI) was 60° and a manual precision ZnSe polarizer (PIKE Technologies) was used to obtain a p-polarized incident IR beam. The IR beam was filtered with a Bruker F321-H optical filter (<4000 cm^{-1}) before entering the detector. The measurement parameters used were an aperture of 8 mm, a resolution of 4 cm^{-1} and 32 scans per spectrum. Fourier transformation was performed using the Mertz phase correction and Norton-Beer, Medium apodization function with a phase resolution of 32 and a zerofilling factor of 4. Bruker OPUS software was used for IR data collection and analysis. The experimental setup and the procedures for the preparation and electrochemical polishing of the Au film are described elsewhere.[9]

Electrochemical measurements

Electrochemical measurements were conducted on a BioLogic VSP-300 potentiostat. A platinum coil was used as the counter electrode. The reference electrode was typically Ag/ Ag^+ (10 mM $AgNO_3$) in 0.2 M TBAP in MeCN. Once, Ag/AgCl in 0.2 M solution of $TBAPF_6$ in MeCN was used (noted in the captions of Figures S37-S40). The Ag/Ag^+ electrode was calibrated with reference to the redox potential of the $K_3[Fe(CN)_6]/K_4[Fe(CN)_6]$ (=+161 mV vs. Ag/AgCl @ 25°C) couple in 3 M $KCl_{(aq.)}$ and the potentials were corrected and reported versus Ag/AgCl. The Ag/Ag^+ electrode was also referenced against Fc/Fc^+ before and after each measurement to ensure its stability. All electrochemical measurements were conducted with 0.2 M $TBAPF_6$ in MeCN solution as the electrolyte and under continuous N_2 flow.

Computational details

All calculations were performed using the Quickstep method of the CP2K program package.[10] For the hybrid Gaussian and plane waves framework, the Kohn-Sham DFT was applied.[11,12] The interactions with the atomic cored were described though the Goedecker-Teter–Hutter (GTH) pseudopotentials[13] and the molecular orbitals of the valence electrons expanded using Gaussian type orbitals. For the description of lighter elements (H, C, N, O, Cl, S) was done by using a triple zeta polarized basis sets TZV2P-MOLOPT-GTH and a double zeta basis set for the heavy atoms (Au and Re) DZVP-MOLOPT-SR-GTH. For hybrid calculations, auxiliary uncontracted basis sets

were applied to accelerate the calculations using cFIT3 for H, C, N, O, Cl and S, cFIT12 for Au and cFIT10 for Re. For general generalized gradient calculations (GGA) the plane wave cutoff was set to 600 Ry, while for the use in vibrational analysis and implicit solvent calculations the cutoff was set to 800 Ry for proper convergence. All calculations were performed with a periodicity in three dimensions. For all GGA calculations, the Perdew-Burke-Ernzerhof (PBE) functional[14] was used together with rVV10 non-local van-der-Waals potentials[15] and HSE06 for hybrid calculations. The basis of all analysis were done by using 7x7x4 layer Au(111) slab (324 atoms) with a vacuum layer of at least 15 Å above the molecules and HG complexes. Optimization of all structures were done by ab-initio molecular dynamics for a time of 5 ps in the micro-canonical ensemble at 100 K followed by a geometry optimization using the LBFGS optimizer. All structures found convergence at an accuracy of 1x10-6 Hartree. In the AIMD and geometry calculations the bottom two layers of the Au(111) surface were fixed upon relaxation. All other atoms were allowed to relax in all dimensions.

Synthesis and characterization

Synthesis of Re(4'-(adamantan-1-yl)-2,2':6',2"-terpyridine)$(CO)_3Cl$ – 3:

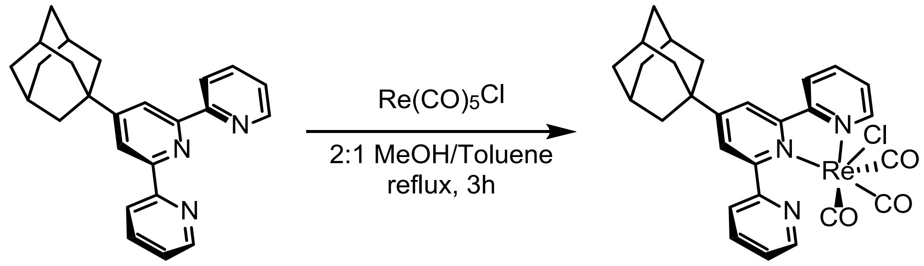

Scheme S1. Synthetic route to Re(4'-(adamantan-1-yl)-2,2':6',2"-terpyridine)$(CO)_3Cl$ guest **3**.

Guest **3** was synthesized by adapting the procedure from literature[16]: 100 mg of 4'-(adamantan-1-yl)-2,2':6',2"-terpyridine (0.27 mmol, 1 equiv.) and 98.4 mg $Re(CO)_5Cl$ (0.27 mmol, 1 equiv.) were refluxed in 30 mL 2:1 MeOH/Toluene mixture. After 3h, the reaction mixture was cooled down to room temperature and the solvent was removed under vacuum. After recrystallization from MeOH, the yellow precipitate was filtered and washed with ice cold MeOH and Et_2O (67%).

HRMS ESI+: *m/z* $[M\text{-}Cl]^+$ $C_{28}H_{25}N_3O_3Re$ calc. 638.1448 found 638.1449

^{1}H NMR (500 MHz, Chloroform-d) δ 9.10 (d, J = 5.4 Hz, 1H), 8.82 (d, J = 4.9 Hz, 1H), 8.28 (d, J = 8.1 Hz, 1H), 8.19 (d, J = 2.0 Hz, 1H), 8.08 (td, J = 7.9, 1.7 Hz, 1H), 7.96 (d, J = 4.4 Hz, 2H), 7.70 (d, J = 1.9 Hz, 1H), 7.52 (dd, J = 7.9, 4.9 Hz, 2H), 2.21 – 2.15 (m, 3H), 2.00 (d, J = 2.8 Hz, 6H), 1.88 – 1.75 (m, 6H).

^{13}C NMR (126 MHz, Chloroform-d) δ 196.93, 193.98, 189.97, 164.06, 157.12, 156.48, 153.25, 148.91, 138.77, 137.89, 126.75, 126.38, 125.36, 125.12, 123.67, 119.71, 77.41, 77.16, 76.90, 43.28, 42.18, 37.49, 36.34, 28.66, 28.49.

Single crystals were obtained by slow diffusion of Et_2O into MeCN/toluene mixture of the compound.

a)

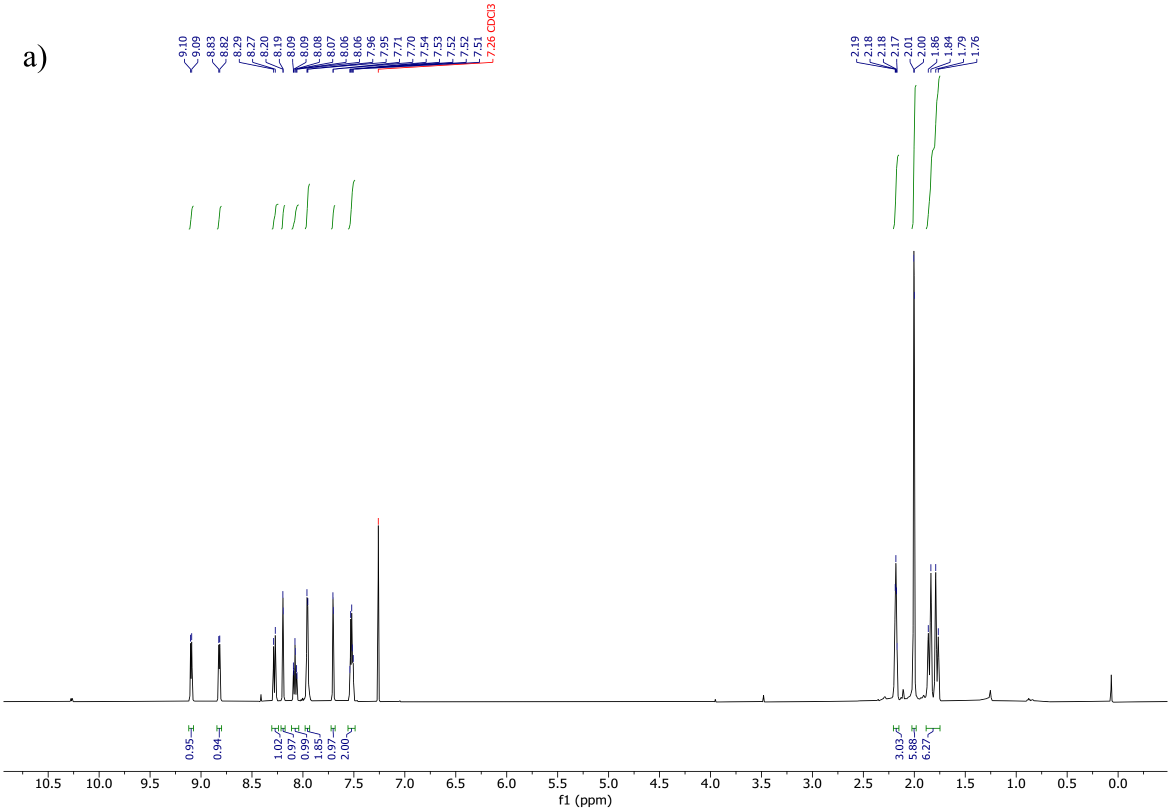

b)

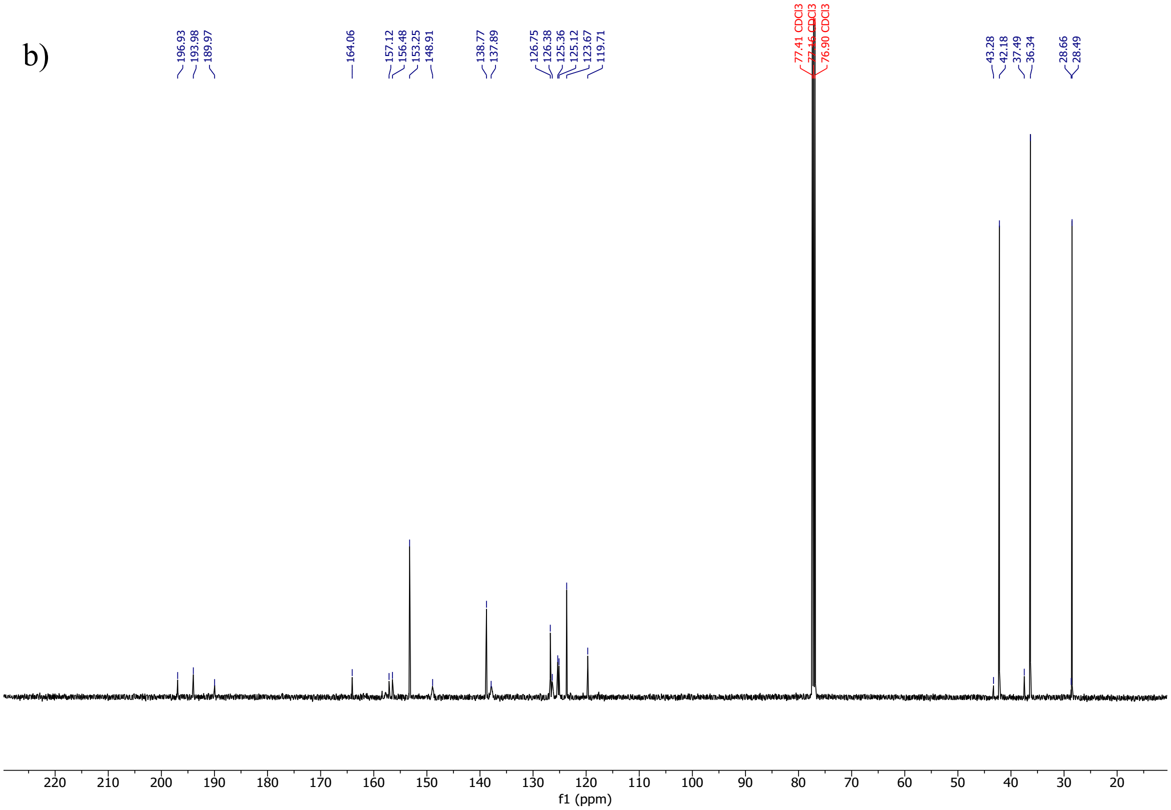

Figure S1. (a) ^{1}H & (b) ^{13}C-NMR of **3** in $CDCl_3$.

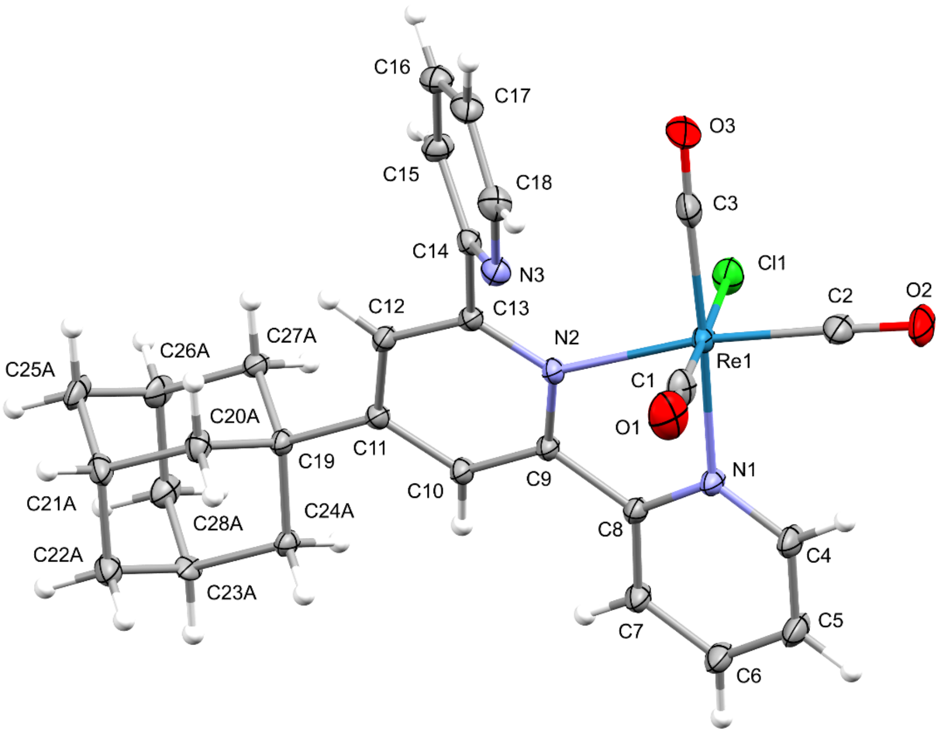

Figure S2. The molecular structure of **3**. Displacement ellipsoids are drawn at 30% probability level. The second component of the disordered adamantane is omitted for clarity.

Table S1. Crystal Data and Structure Refinement of **3**

Empirical formula	$C_{28}H_{25}ClN_3O_3Re$
Formula weight	673.16
Temperature/K	160.0(1)
Crystal system	orthorhombic
Space group	Pbca
a/Å	17.2623(3)
b/Å	11.6959(2)
c/Å	25.0987(3)
α/°	90
β/°	90

γ/°	90
Volume/Å^3	5067.38(14)
Z	8
ρ_{calc}g/cm^3	1.765
μ/mm^{-1}	10.641
F(000)	2640.0
Crystal size/mm^3	0.12 × 0.04 × 0.03
Radiation	Cu Kα (λ = 1.54184)
2Θ range for data collection/°	7.044 to 148.876
Index ranges	-21 ≤ h ≤ 18, -14 ≤ k ≤ 14, -31 ≤ l ≤ 31
Reflections collected	29196
Independent reflections	5170 [R_{int} = 0.0245, R_{sigma} = 0.0173]
Data/restraints/parameters	5170/96/407
Goodness-of-fit on F^2	1.047
Final R indexes [I>=2σ (I)]	R_1 = 0.0198, wR_2 = 0.0502
Final R indexes [all data]	R_1 = 0.0218, wR_2 = 0.0513
Largest diff. peak/hole / e Å^{-3}	0.67/-0.68

Synthesis of Heptakis(6-p-thio)(2,3-OAcetyl)-β-CD – 2:

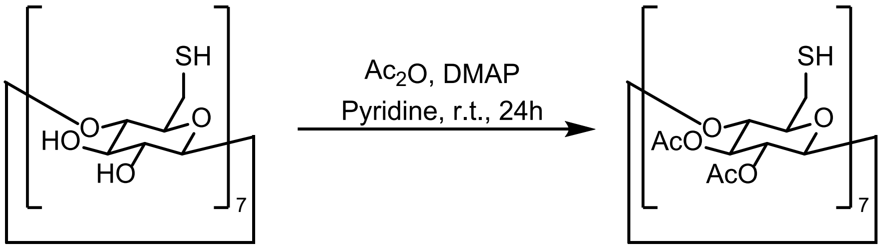

Scheme S2. Synthetic route to heptakis(6-p-thio)(2,3-OAcetyl)-β-CD host **2**.

100 mg of **1** (80.2 umol, 1 equiv.) was dissolved in 3 mL dry pyridine. After the reaction temperature was dropped to 0°C in the ice bath, 107 uL Ac_2O (1.10 mmol, 14 equiv.) and 274.2

mg DMAP (2.19 mmol, 28 equiv.) was added. After 10 minutes, the ice bath was removed and the reaction was left stirring for 24h at room temperature. The reaction mixture was then diluted with EtOAc and the organic phase was washed with 0.2 M HCl (3 x 15 mL), sat. $NaHCO_3$ (2 x 15 mL) and brine (2 x 15 mL). The organic phase was dried over $MgSO_4$, filtered and the solvent was removed under vacuum. The remaining crude was then dissolved in small amounts of acetone and this mixture was then added to 500 mL pentane dropwise. The white precipitate was then filtered and washed with pentane (70%).

HRMS ESI+: *m/z* $[M+Na]^+$ $C_{70}H_{98}O_{42}S_7Na$ calc. 1857.3470 found 1857.3454 & *m/z* $[M+2Na]^{2+}$ calc. 940.1681 found 940.1679

^{1}H NMR (500 MHz, Chloroform-*d*) δ 5.46 – 4.82 (m, 13H), 4.32 – 3.83 (m, 11H), 3.80 – 3.56 (m, 10H), 3.49 (t, *J* = 4.9 Hz, 4H), 3.42 – 3.06 (m, 11H), 2.50 – 1.97 (m, 49H).

^{13}C NMR (126 MHz, $CDCl_3$) δ 194.83, 194.40, 169.61, 100.96, 100.45, 86.81, 80.42, 77.41, 77.16, 76.91, 74.13, 72.60, 71.90, 70.68, 70.31, 70.03, 30.86, 30.82, 30.71, 21.30, 21.08, 21.03, 20.80.

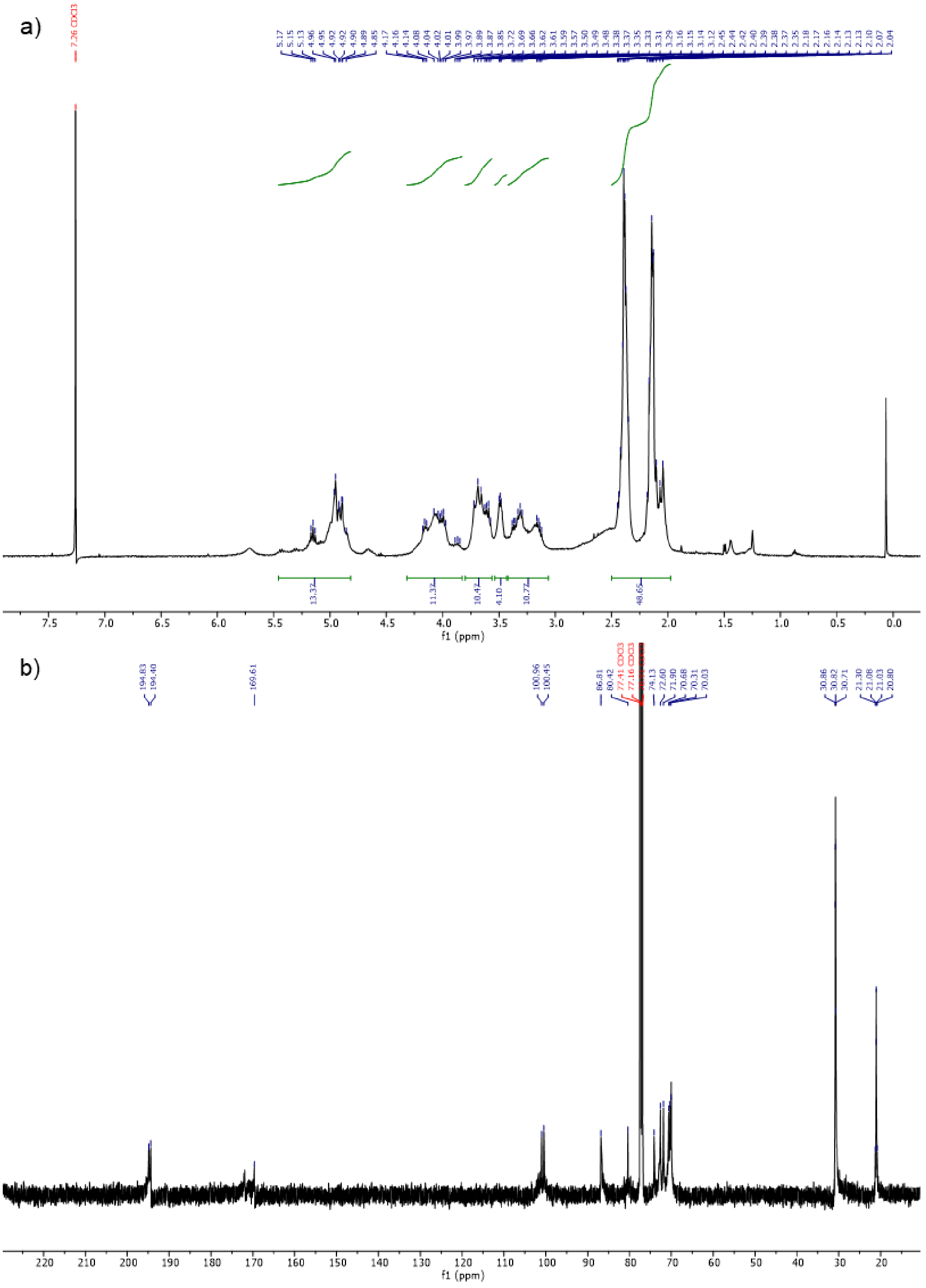

Figure S3. ^{1}H & ^{13}C-NMR of **2** in $CDCl_3$.

The same procedure was used to synthesize β-CD-$(OAc)_{21}$ with only changing the equivalents of Ac_2O to 28 and DMAP to 56. The pure product was obtained with 64% yield.

HRMS ESI+: *m/z* $[M+Na]^+$ $C_{84}H_{112}O_{56}Na$ calc. 2039.5808 found 2039.5827 & *m/z* $[M+2Na]^{2+}$ calc. 1031.2850 found 1031.2846

^{1}**H NMR** (400 MHz, Chloroform-*d*) δ 5.44 – 5.23 (m, 6H), 5.09 (d, *J* = 3.9 Hz, 8H), 4.80 (dd, *J* = 9.5, 3.9 Hz, 7H), 4.57 (d, *J* = 12.2 Hz, 7H), 4.27 (dd, *J* = 12.6, 4.2 Hz, 7H), 4.15 (d, *J* = 9.2 Hz, 7H), 3.83 – 3.64 (m, 7H), 2.20 – 2.01 (m, 63H).

Procedures for guest adsorption SEIRAS measurements

Physisorption

The electrochemically polished and washed Au film was exposed to 0.1 mM solution of guest **3** in MeOH. After 1 h the guest solution was removed and the film was washed twice with MeOH and then MeOH was left in the cell for 10 minutes to remove any loosely bound species. During both 1 h guest **3** solution exposure and 10 minute MeOH wash, SEIRA spectra were recorded every 5 minutes.

HG complexation

The electrochemically polished and washed Au film was exposed to 0.1 mM solution of host **1** in DMSO. After 0.5-1 h the host solution was removed and the film was washed twice with DMSO and then DMSO was left in the cell for 10 minutes. Then the same cleaning procedure was repeated with MeOH. Afterwards, 0.1 mM solution of guest **3** in MeOH was placed into the cell following the procedure given above for physisorption.

SEIRA spectra during host 2 adsorption

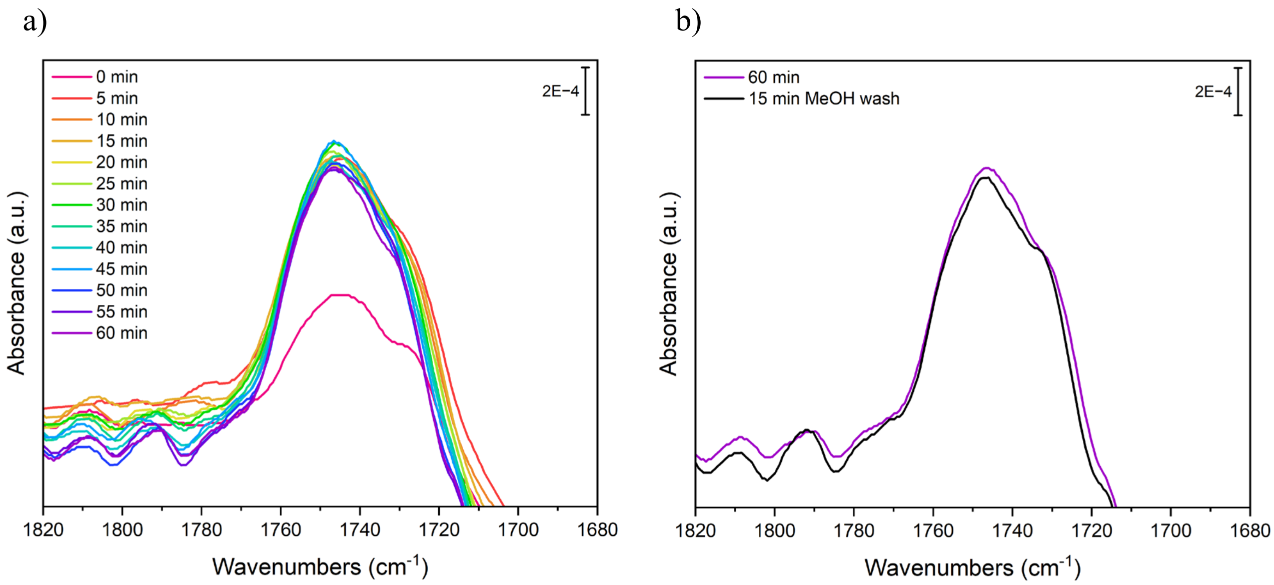

Figure S4. SEIRA spectra recorded (a) during 1h immersion of Au surface in 0.1 mM solution of host **2** in MeOH and (b) after the MeOH wash, using the clean Au surface in MeOH as background spectrum. Rainbow colored lines in (a) show the SEIRA spectra obtained from 0 minute (pink) to

60 minute (purple) with 5 minute intervals. The band between 1740-1760 cm^{-1} was assigned to be C=O stretching mode from the acetyl groups. This band is visible as soon as the host solution is in contact with the Au surface. After 5 minutes, the absorbance intensity reaches a plateau, indicating that the host binding is completed withing few minutes.

QCM-D measurements of host 2

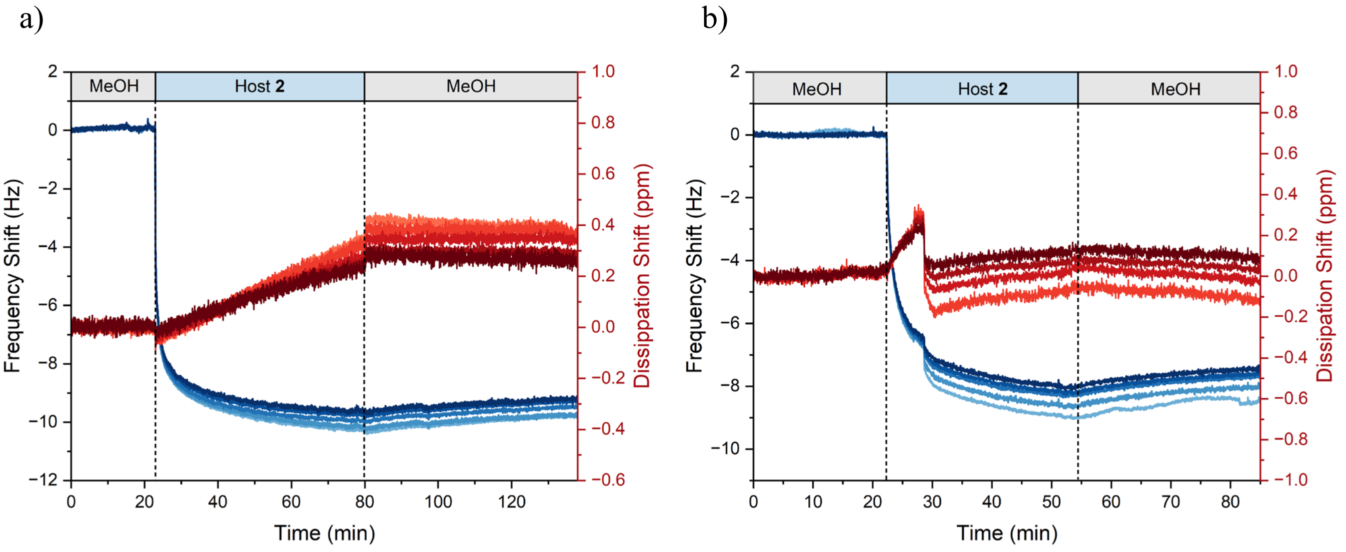

Figure S5. Two independent replicate experiments of QCM-D measurements for detection of the host **2** binding on Au surface. The shaded blue and red lines show the frequency and dissipation shift, respectively. The light to darker colors denote the shifts in the 5th to 13th overtones. The spike in (b) is due to the small pressure fluctuations when changing the solution and the more dispersed overtones could also be due to this. The observed frequency shifts of the 7th overtone were -9.7 Hz (a) & -8 Hz (b), using the Saurbrey equation, the corresponding surface density values were found to be 0.56 nm^{-2} & 0.46 nm^{-2}, respectively. The fast stabilization of the frequency shift after flowing the host **2** solution in both measurements, shows that this discrepancy is more likely due to sample-to-sample variation rather than the effect of the flow time.

NMR titration studies

In order to test the ability of host **2** to form HG complexes, NMR titration studies were done by using solution phase HG complexes.[17-19] For this, a 1 mM host solution (either with β-CD or per-acetylated β-CD) was prepared in 3 mL 7:3 MeOD/D_2O mixture. 0.6 mL of this solution was used to record ^{1}H-NMR. The remaining 2.4 mL solution was used to prepare the HG mixture by dissolving 1-adamantanecarboxylic acid to a 5 mM concentration, which was used as the guest instead of guest **3** due to solubility limitations. This HG mixture was then added stepwise into the NMR tube containing the host solution. After each addition, ^{1}H-NMR spectra were recorded. The chemical shift changes δ (ppm) of the host were plotted with supramolecular *BindFit* to obtain the binding constants.[20]

A binding constant of $K_{11} = 1210 \pm 60$ M^{-1} was obtained for β-CD (Figure S6). However, minimal to no changes were observed in the ^{1}H-NMR spectra for the per-acetylated β-CD (Figure S7), which made determination of the binding constant unreliable, with a fitting error exceeding 100%.

The lack of a clear change in the ^{1}H-NMR spectra indicates that even with a less bulky guest, the acetyl groups hinder HG complexation. Consequently, host **2** is unsuitable for surface immobilization of guests.

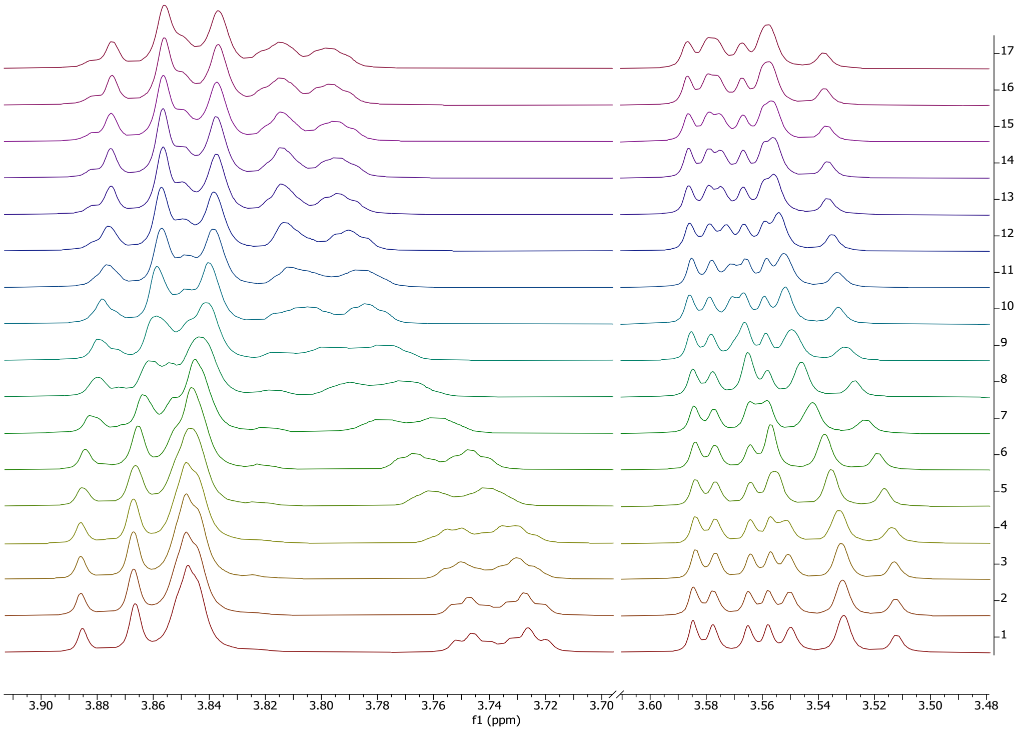

Figure S6. ^{1}H-NMR spectra of the HG titration of β-CD with 1-adamantanecarboxylic acid in 7:3 MeOD/D_2O with increasing H:G ratio from 1:0 (bottom) to 1:3 (top) according to the Table S2. The resulting binding constant was K_{11}=1210.28 ± 60.5 M^{-1}.

Table S2. Data of the host-guest titration of β-CD with 1-adamantanecarboxylic acid.

Host c(M)	Guest c(M)	G/H equiv.	y1: Shift (ppm)	y2: Shift (ppm)
0.001	0	0	3.726733	3.512242
0.001	4.95E-05	0.049505	3.727453	3.512757
0.001	0.000122	0.121951	3.730256	3.513198
0.001	0.000238	0.238095	3.732819	3.513932
0.001	0.000349	0.348837	3.741227	3.516504
0.001	0.000455	0.454545	3.747073	3.519076
0.001	0.000833	0.833333	3.758845	3.523485

0.001	0.001154	1.153846	3.770296	3.527454
0.001	0.001429	1.428571	3.777103	3.530246
0.001	0.001774	1.774194	3.78431	3.532818
0.001	0.002059	2.058824	3.785831	3.533333
0.001	0.002297	2.297297	3.790396	3.53539
0.001	0.0025	2.5	3.793919	3.536786
0.001	0.002778	2.777778	3.793919	3.536786
0.001	0.003	3	3.793919	3.537007
0.001	0.003214	3.214286	3.795201	3.537742
0.001	0.003446	3.445596	3.796482	3.537742

Link to *BindFit* (β-CD):

http://app.supramolecular.org/bindfit/view/056db83c-5896-42da-bb2c-7872c7096c9e

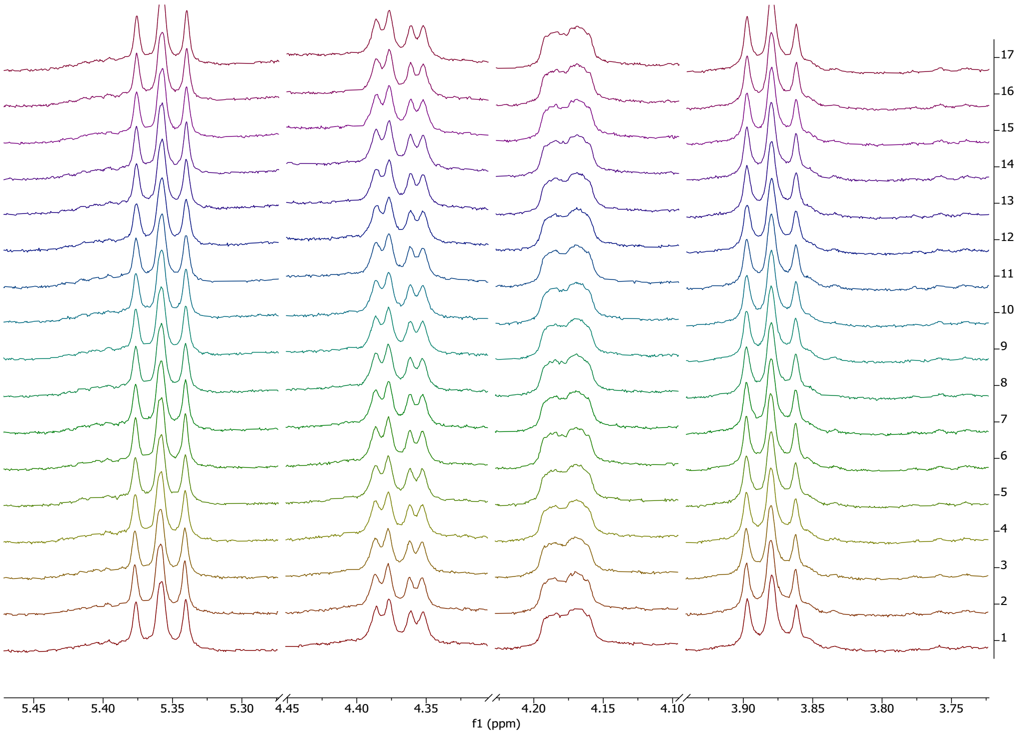

Figure S7. ^{1}H-NMR spectra of the HG titration of per-acetylated β-CD with 1-adamantanecarboxylic acid in 7:3 MeOD/D_2O with increasing H:G ratio from 1:0 (bottom) to 1:3 (top) according to the Table S3. The resulting binding constant was K_{11}=8011.31 ± 8241.13 M^{-1}.

Table S3. Data of the host-guest titration of per-acetylated β-CD with 1-adamantanecarboxylic acid.

Host c(M)	Guest c(M)	G/H equiv.	y1: Shift (ppm)	y2: Shift (ppm)	y3: Shift (ppm)
0.001	0	0	2.108508	3.90004	5.343797
0.001	4.95E-05	0.049505	2.108508	3.90004	5.343431
0.001	0.000122	0.121951	2.108508	3.900298	5.343431
0.001	0.000238	0.238095	2.108508	3.900298	5.343193
0.001	0.000349	0.348837	2.108508	3.900298	5.343193
0.001	0.000455	0.454545	2.108593	3.900298	5.343193
0.001	0.000833	0.833333	2.108593	3.900298	5.343193
0.001	0.001154	1.153846	2.108678	3.900298	5.343193
0.001	0.001429	1.428571	2.108678	3.900298	5.343193
0.001	0.001774	1.774194	2.108678	3.900298	5.343193
0.001	0.002059	2.058824	2.108678	3.900298	5.343193
0.001	0.002297	2.297297	2.108678	3.90054	5.343193
0.001	0.0025	2.5	2.108508	3.90054	5.342956
0.001	0.002778	2.777778	2.108508	3.90054	5.342956
0.001	0.003	3	2.108508	3.90054	5.342956
0.001	0.003214	3.214286	2.108508	3.90054	5.342956
0.001	0.003446	3.445596	2.108508	3.90054	5.342956

Link to *BindFit* (per-acetylated β-CD):

http://app.supramolecular.org/bindfit/view/651e899b-9f94-4a44-a174-ee02e8c44e8d

FT-IR characterization of guest 3

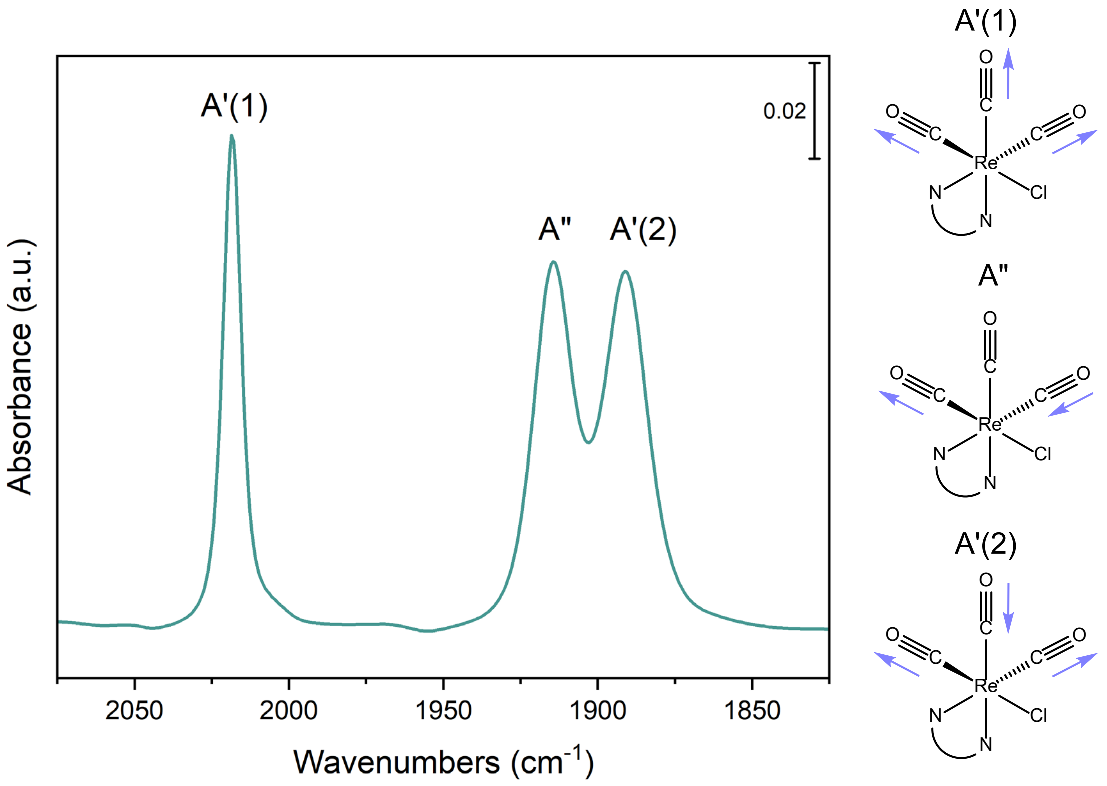

Figure S8. FT-IR spectrum of 1 mM guest **3** in DMF. The FT-IR spectrum of DMF was used as the background. The visualization of the assigned stretching modes are shown on the right.

SEIRAS replicate measurements of HG complexation

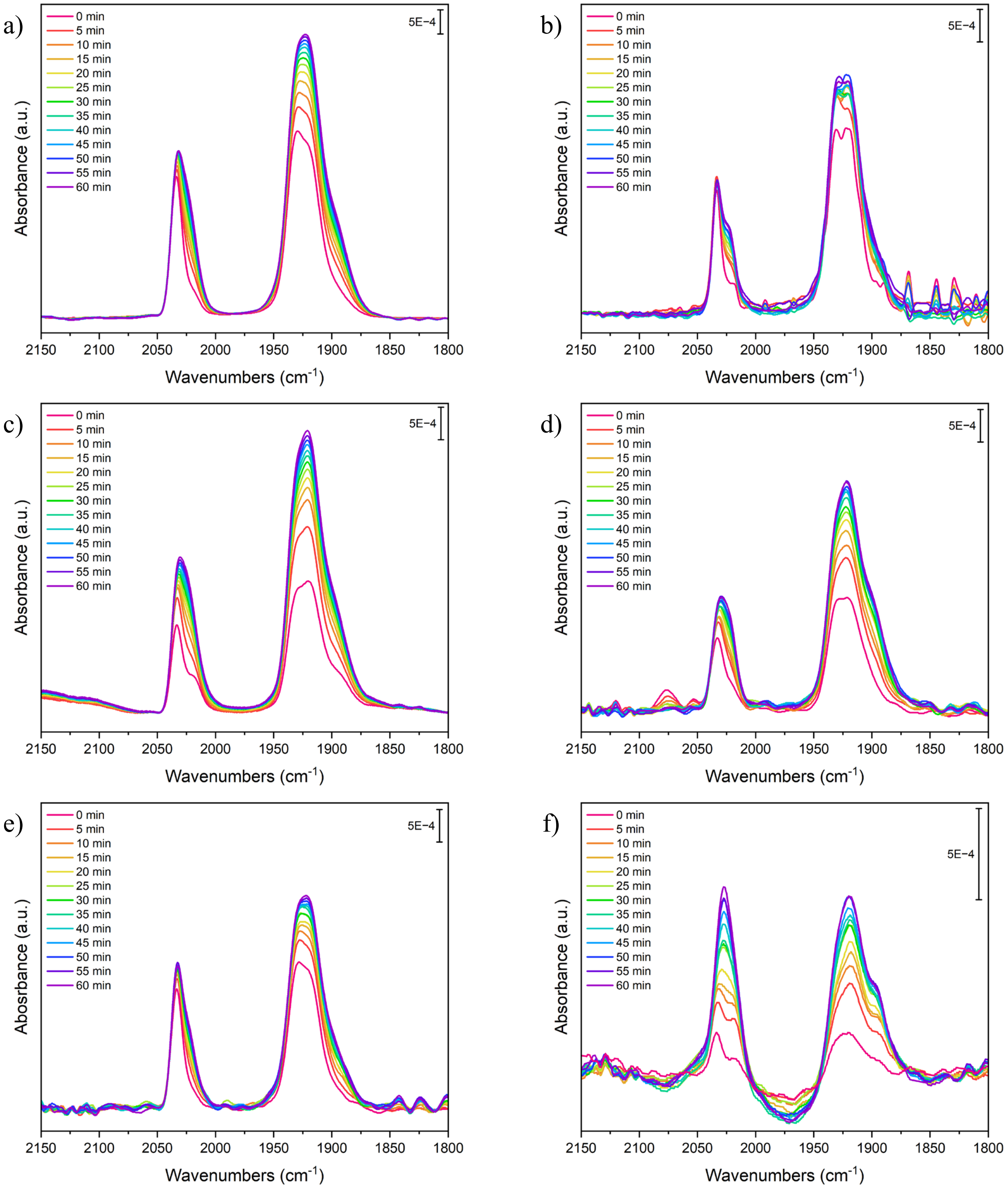

Figure S9. Six independent replicate experiments of SEIRAS measurements during 1h immersion of a host **1** modified Au surface in guest **3** solution, using the clean Au surface in MeOH as background spectrum. Rainbow-colored lines denote SEIRA spectra obtained from 0 min (pink) to 60 min (purple) immersion time.

SEIRAS replicate measurements of MeOH wash after HG complexation

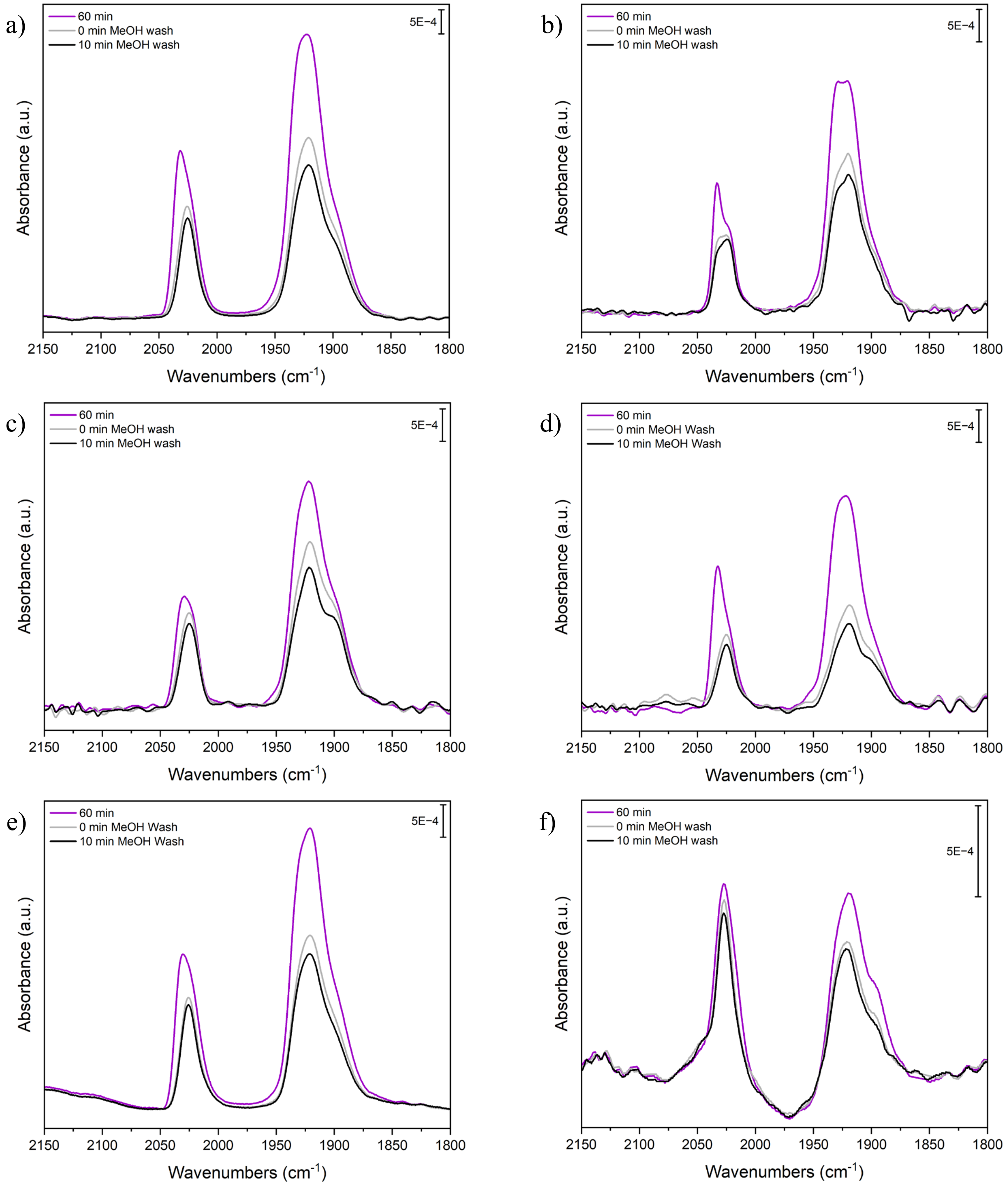

Figure S10. Six independent replicate experiments of SEIRAS measurements after 60 min HG complexation (purple), 0 min MeOH wash (grey) and 10 min MeOH wash (black), using the clean Au surface in MeOH as background spectrum. The 60 min data are from Figure S9. The data shows that the shoulder band around 2033 cm^{-1} disappears after the addition of MeOH with the exceptions of (b) & (f).

SEIRAS replicate measurements of physisorption

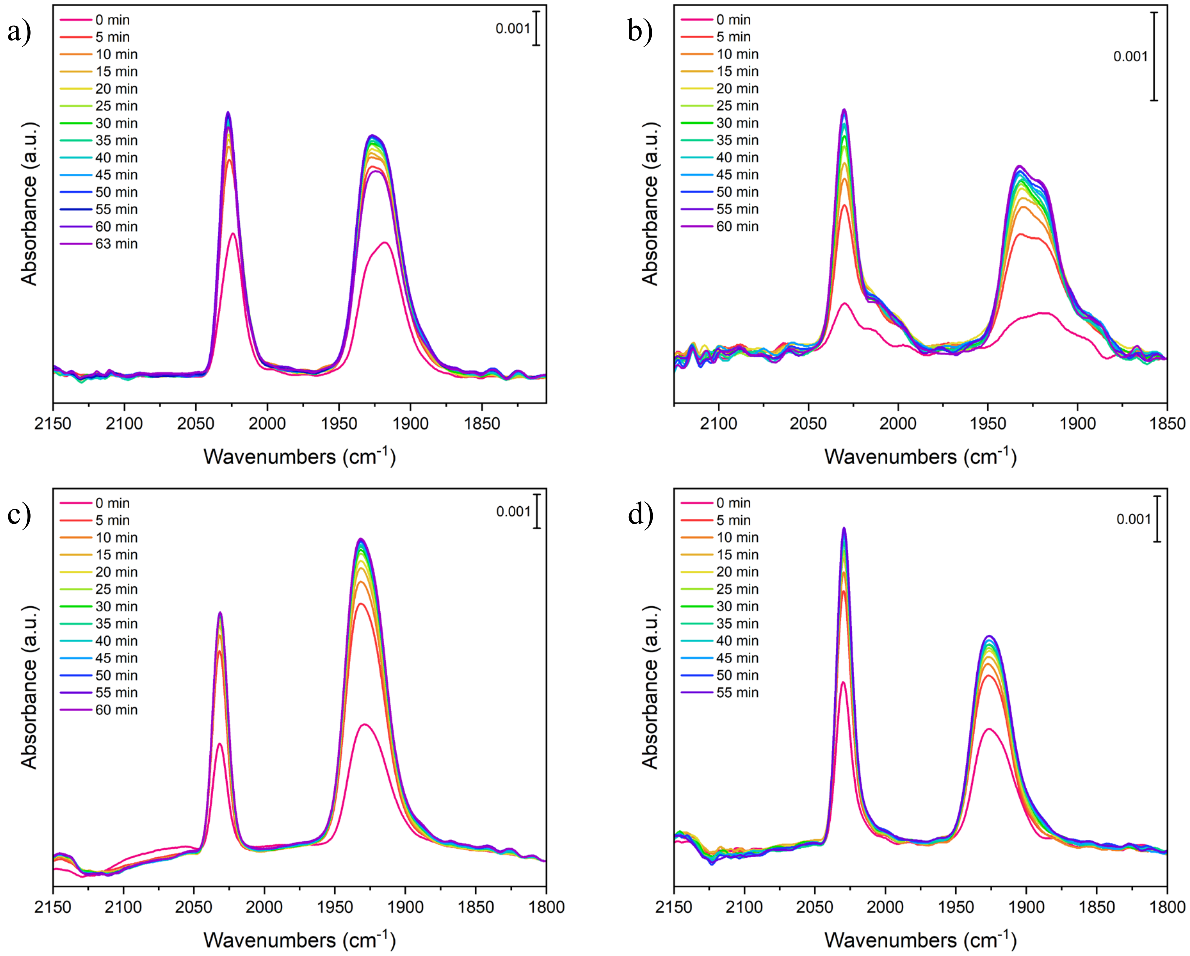

Figure S11. Four independent replicate experiments of SEIRAS measurements during 1h immersion of a clean Au surface in guest **3** solution, using the clean Au surface in MeOH as background spectrum. Rainbow-colored lines denote SEIRA spectra obtained from 0 min (pink) to 60 min (purple) immersion time.

SEIRAS replicate measurements of MeOH wash after physisorption

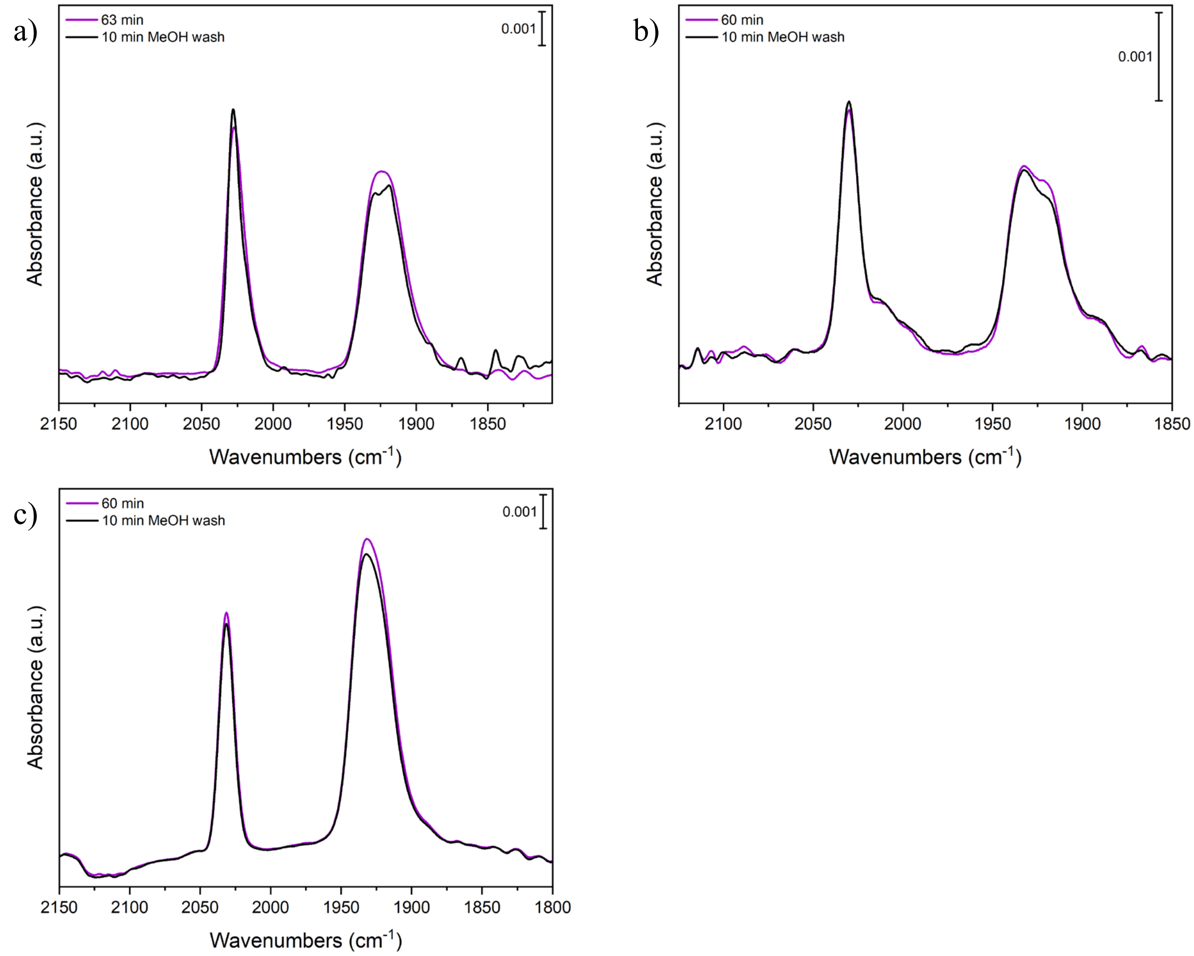

Figure S12. Three independent replicate experiments of SEIRAS measurements after 60 min physisorption (purple) and 10 min MeOH wash (black), using the clean Au surface in MeOH as background spectrum. The 60 min data are from Figure S11. Almost no changes were observed after MeOH wash.

Data fitting of the SEIRA spectra

All SEIRA spectra were analyzed by using OriginPro 2022 (OriginLab). Generally, the data between ~1975 and ~2100 cm^{-1} were selected to perform peak fitting of the A'(1) band using peak analyzer. First, a linear baseline correction was done to each spectrum by choosing two anchoring points. Then, the peak finding was performed with a local maximum method using the second derivative of the spectrum. The A'(1) band was then fitted with a Voigt function which gave the band location, integrated band area and full width at half maximum (FWHM). The integrated band area obtained from the fittings were then used to investigate the changes in the integrated band area as a function of immersion time, which were used for determination of the binding kinetics. The obtained band positions were used to investigate the A'(1) band position as a function of applied potential.

SEIRAS HG band growth overtime

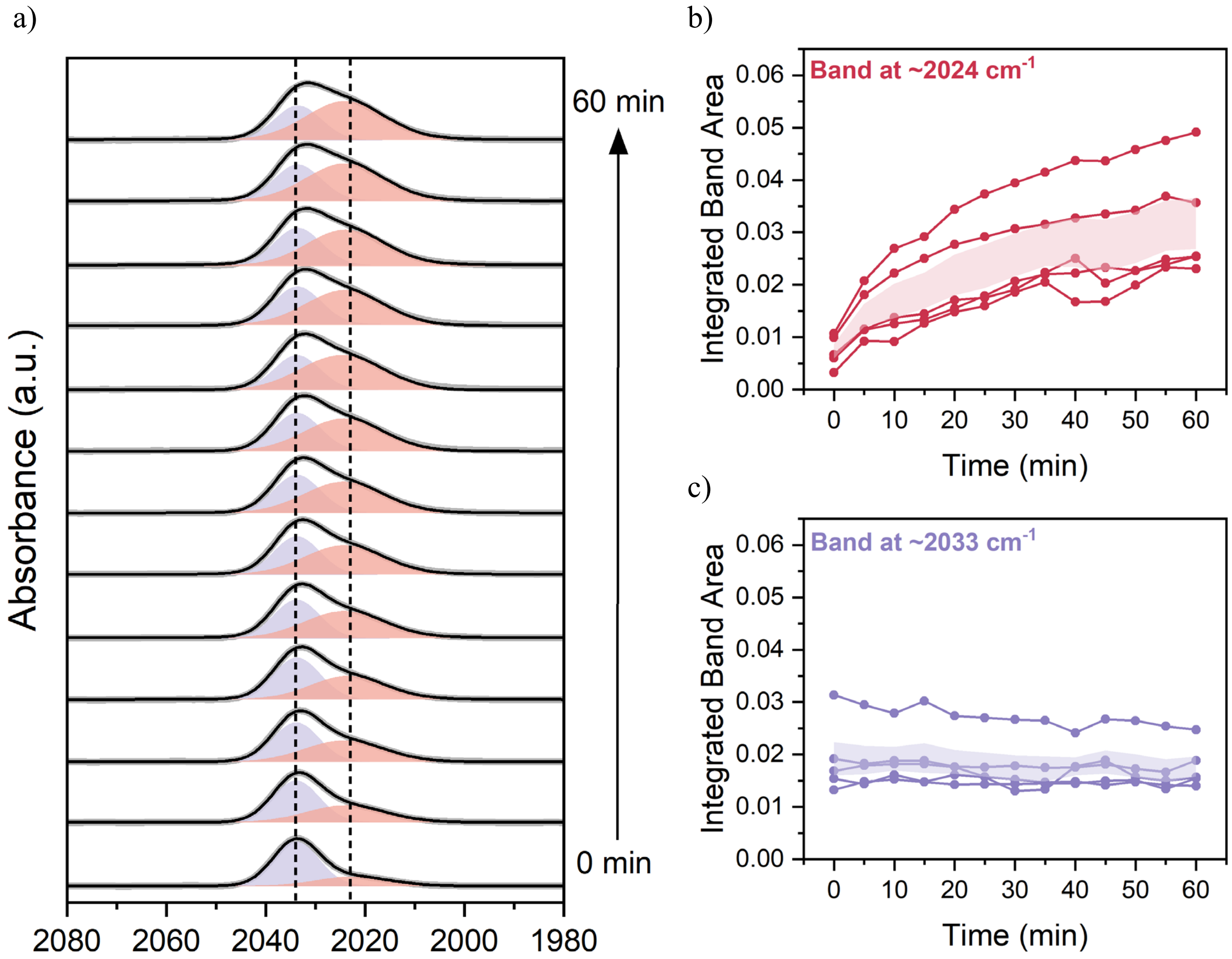

Figure S13. The changes in the integrated band area during HG complexation, obtained from Voigt fit. (a) An example of the Voigt fit for the A'(1) band from 0 to 60 minutes guest **3** immersion time. The fittings show the presence of the two bands during HG complexation (data from Figure S9a). The black lines show the cumulative fit peak & the grey line is the experimental data. Integrated band area as a function of time for the band around (b) 2024 cm^{-1} and (c) 2033 cm^{-1}. The connected dots are data obtained from five independent SEIRA measurements (Figure S9a-e) and the averaged data are shown as the light colored band in the respective plots. The increase in the integrated band area as a function of time in (b) suggests that this band corresponds to a binding event. Whereas almost no change or a slight decrease is observed for the band around 2033 cm^{-1}. These data, together with the disappearance of the band around 2033 cm^{-1} after MeOH wash (Figure S10), suggests that the band around 2024 cm^{-1} corresponds to the HG complexation and the one around 2033 cm^{-1} is the weakly-bound guests.

FT-IR measurement of solution phase HG complex

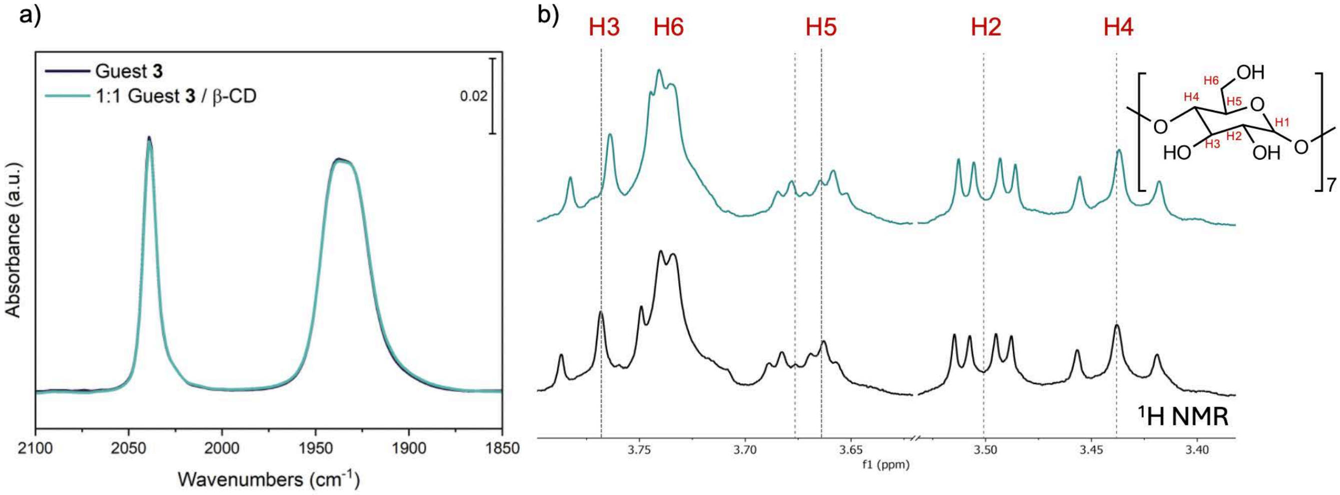

Figure S14. FT-IR spectra of 1 mM solutions of guest **3** (black line) and 1:1 mixture of guest **3** with β-CD (green line). Due to solubility limitations, instead of MeOH, 7:3 MeCN/H_2O mixture was used to prepare the sample solutions. FT-IR spectrum of the solvent mixture was used as background. No changes were observed when comparing the guest **3** spectra with the HG spectra. (b) ^{1}H-NMR spectra of β-CD (black line) and a 1:1 mixture of guest **3** with β-CD (blue line) in 7:3 CD_3CN/D_2O. The dashed lines guide the eye to the shifts upon HG binding. H3 and H5, which point into the cavity, show the strongest response. The ^{1}H NMR shifts confirm that HG interactions occur in the solvent mixture in (a) and do not influence the $\tilde{\nu}_{CO}$ bands.

FT-IR measurements of guest 3 in MeOH and band assignments

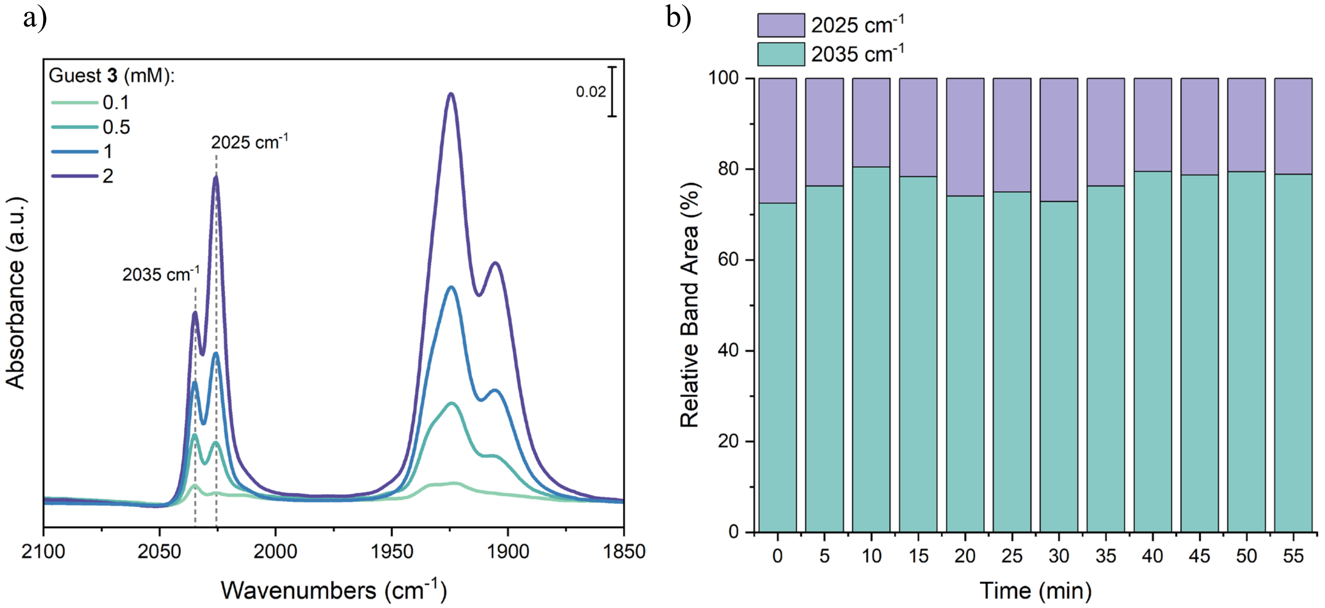

Figure S15. (a) FT-IR spectra of guest **3** solution in MeOH at various concentrations, using the MeOH spectrum as background. At all concentrations, two distinct A'(1) bands were observed

above 2000 cm^{-1}, with their relative intensities varying as a function of concentration. Such behavior was not observed when the FT-IR spectrum of guest **3** was measured in aprotic solvents such as DMF and MeCN, indicating that the appearance of two A'(1) bands in MeOH is due to H-bonded adducts/aggregates.[21,22] Once the guest is adsorbed onto the Au electrode, either through physisorption or HG (after MeOH wash), only one A'(1) band is observed, suggesting the presence of one species on the surface. Previous studies on metal carbonyl complexes show that H-bonding to the carbonyl groups leads to red shifts,[23,24] and the extent of the shift depends on the H-bond strength.[23,25] Based on this, we assign the 2025 cm^{-1} band to the guest **3** molecules with H-bonded carbonyl groups and the 2035 cm^{-1} band to the isolated guest **3** molecules; hence, we use the latter band position to compare the relative shifts observed in SEIRAS. The close proximity of the 2035 cm^{-1} band position to that of the weakly bound guests detected in SEIRAS further supports this assignment. In order to rule out a time-dependent conversion from one species to another in MeOH, which could impact the SEIRAS measurements during guest **3** adsorption, we investigated a 0.1 mM solution of guest **3** in MeOH by FT-IR, recording a spectrum every 5 minutes over 1 h: (b) The two A'(1) bands were fitted with Voigt functions to determine their relative band areas as a function of time. As shown in (b), no changes in the relative band areas were observed, suggesting that the H-bonded and isolated species are in equilibrium.

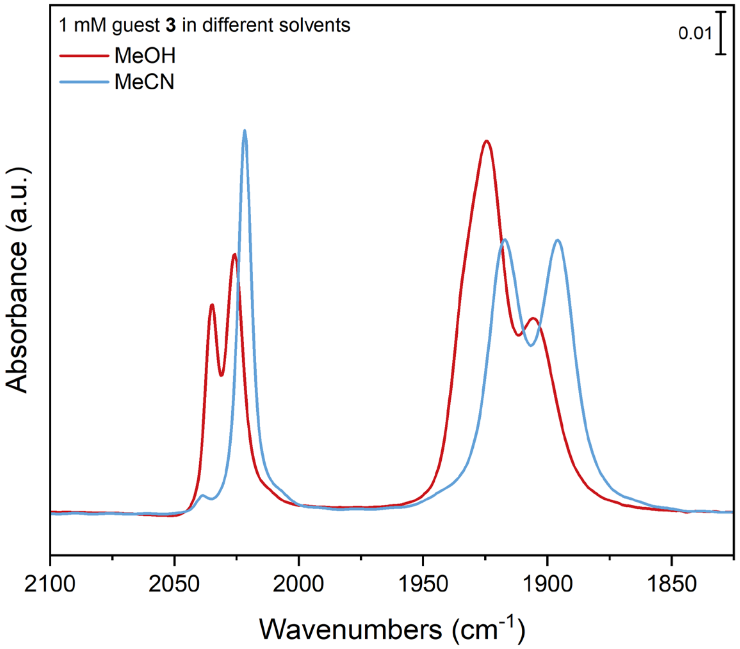

Figure S16. FT-IR spectra of guest **3** in MeOH (red line) and MeCN (blue line). In both measurements, the spectrum of the corresponding neat solvent was used as the background. Upon switching from MeOH to MeCN, a red shift is observed and the A'(1) band associated with the H-bonded adduct disappeared.

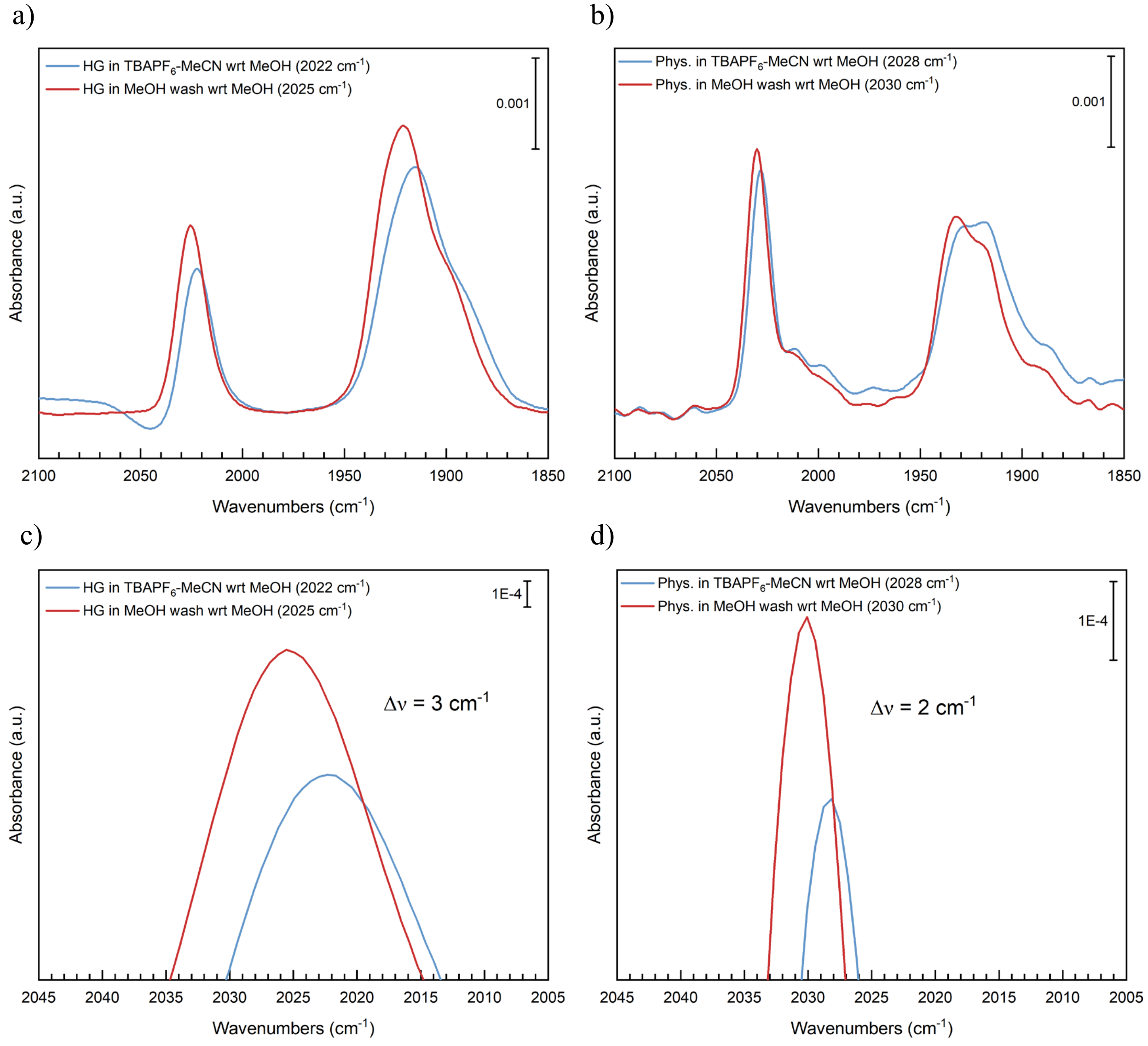

Figure S17. SEIRA spectra of (a) host-bound and (b) physisorbed guest **3** in MeOH (blue lines) and 0.2 M $TBAPF_6$ in MeCN (red lines), using clean Au in MeOH as the background spectrum. Zoom in of the SEIRA spectra showing the A'(1) band of (c) host-bound and (d) physisorbed guest **3**. In both cases a red shift is observed upon switching from MeOH to 0.2 M $TBAPF_6$ in MeCN. No changes in the $\tilde{\nu}_{CO}$ band shapes are observed, further supporting that the H-bonded species observed in the bulk FT-IR spectrum of guest **3** in MeOH is not detected on the surface.

A'(1) band comparisons

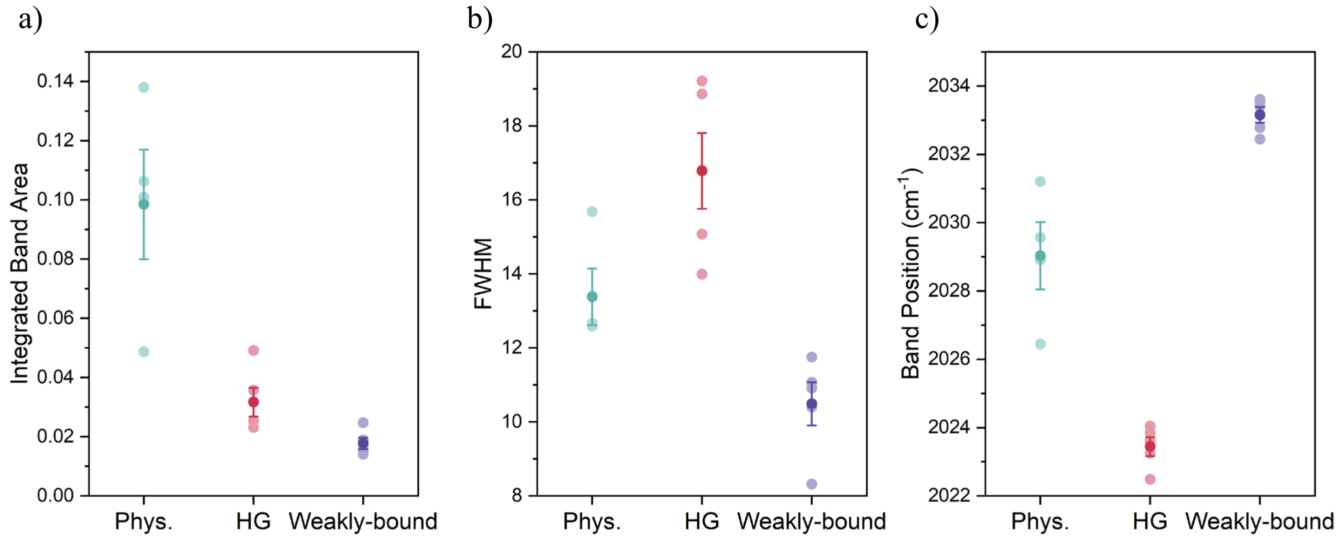

Figure S18. Comparison of the (a) integrated band area, (b) FWHM and (c) band position of the A'(1) band obtained from Voigt fit for the guests that are physisorbed, host-bound and in the bulk solution. The light colored data points show the data obtained from independent replicate experiments (Figures S9a-e & S11). The dark colored data points show the mean value and the error bars show the std. error of the mean.

Analysis of solvent features

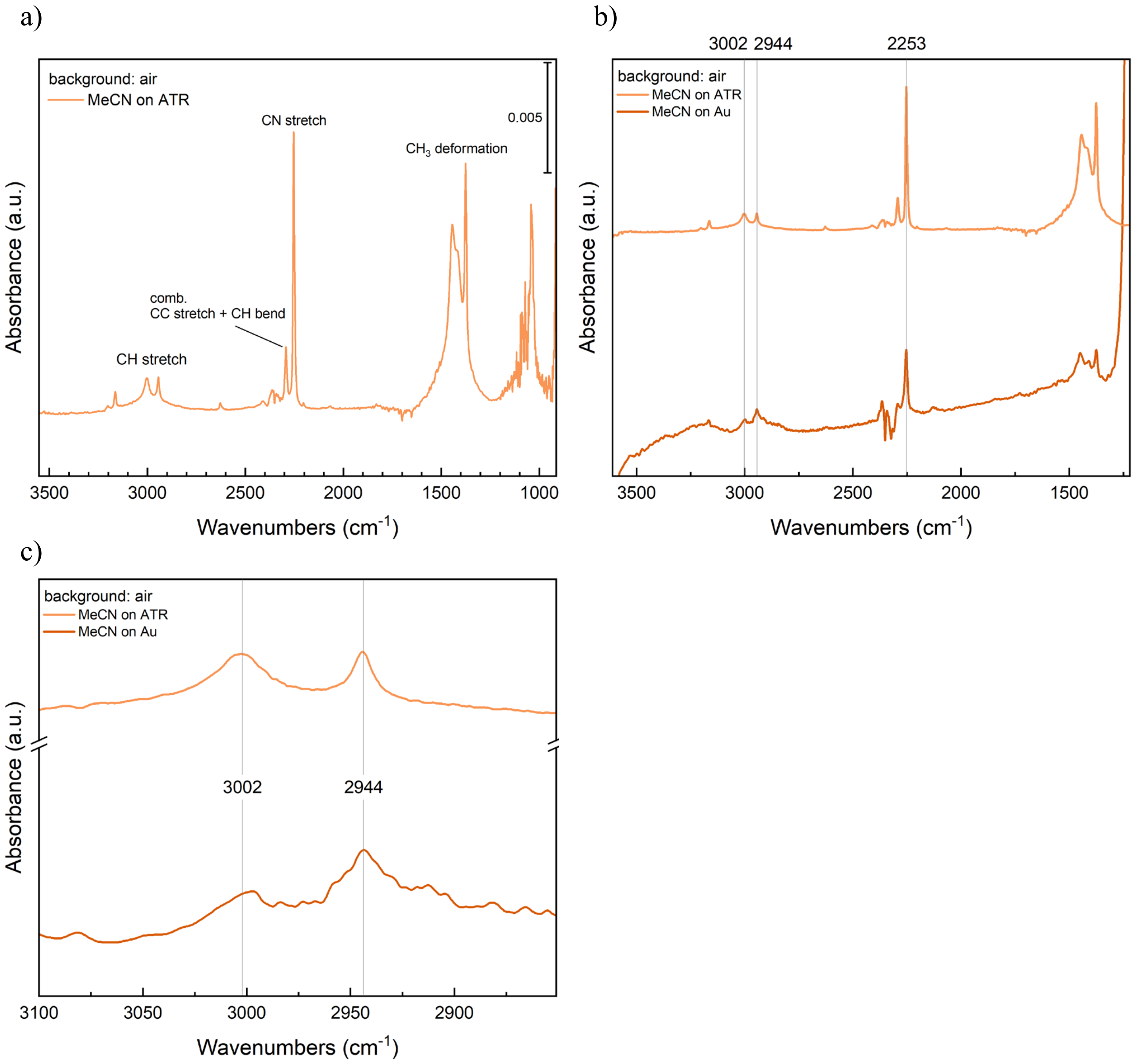

Figure S19. (a) ATR-IR spectrum of MeCN showing the band assignments. (b) MeCN spectra comparisons for ATR-IR (orange line) and SEIRAS (brown line). (c) Zoom in of the CH stretching bands. No discernible CH vibrational shifts were observed between bulk and SEIRAS measurements, hence no change in solvent structure can be observed in presence versus absence of the Au layer. In all spectra, a clean Au surface in air was used as the background.

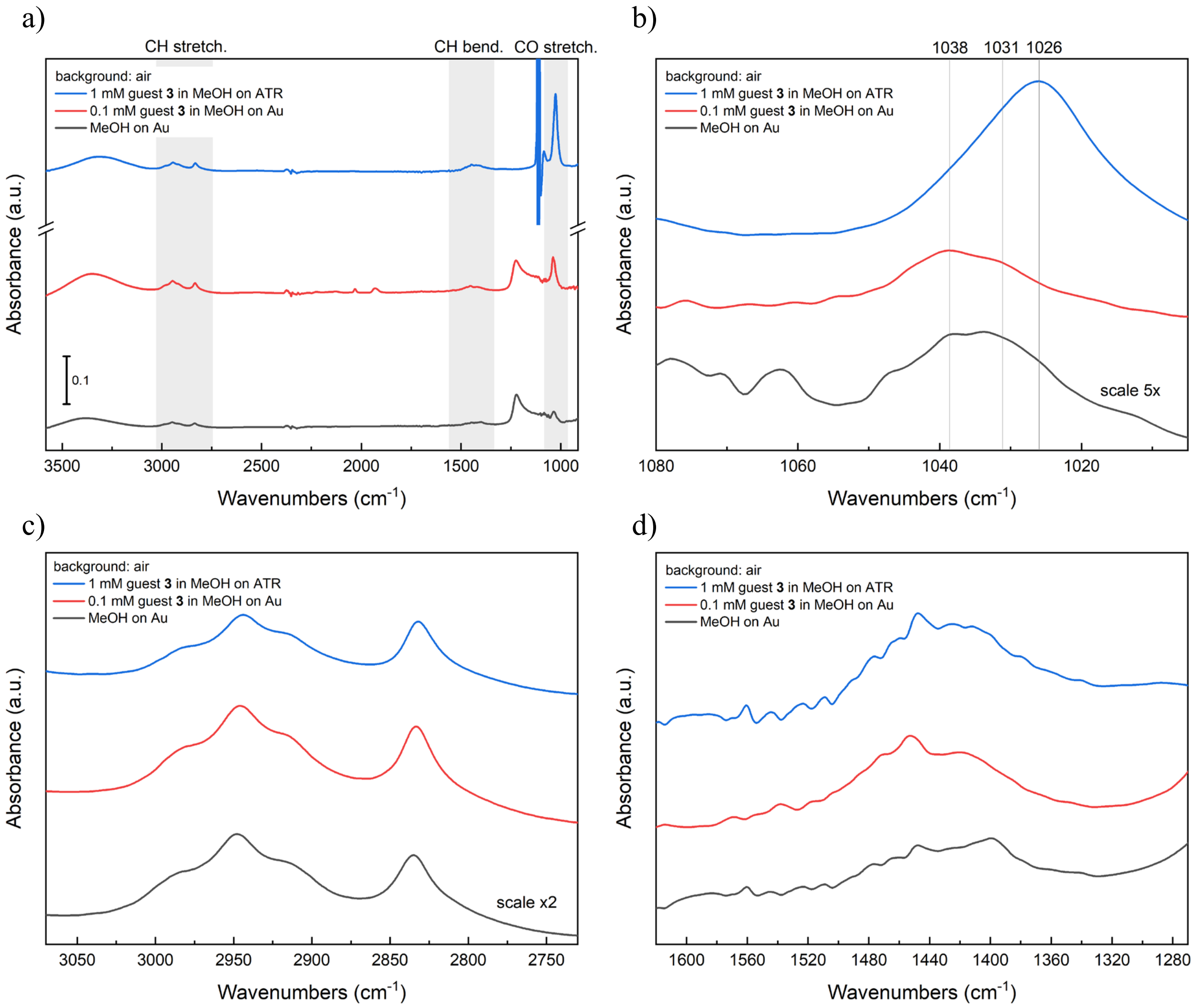

Figure S20. MeOH spectra comparisons obtained from: ATR-IR measurement of guest **3** in MeOH (blue line), SEIRAS measurement of guest **3** in MeOH (red line) and SEIRAS measurement of MeOH (black line). In (a), the entire spectral range is shown, and the bands between 1000-1060 cm^{-1}, 1360-1480 cm^{-1} and 2750-3000 cm^{-1} were assigned to C-O stretching, C-H bending and C-H stretching, respectively. (b) Zoom in of the C-O stretching of MeOH, showing that the CO stretching modes of MeOH are similar on SEIRAS regardless of the presence of guest **3**, while both are blue shifted compared to the band obtained from ATR-IR. Zoom in of (c) C-H stretching and (d) C-H bending showing no obvious changes in the solvent structure. In all spectra, a clean Au surface in air was used as the background.

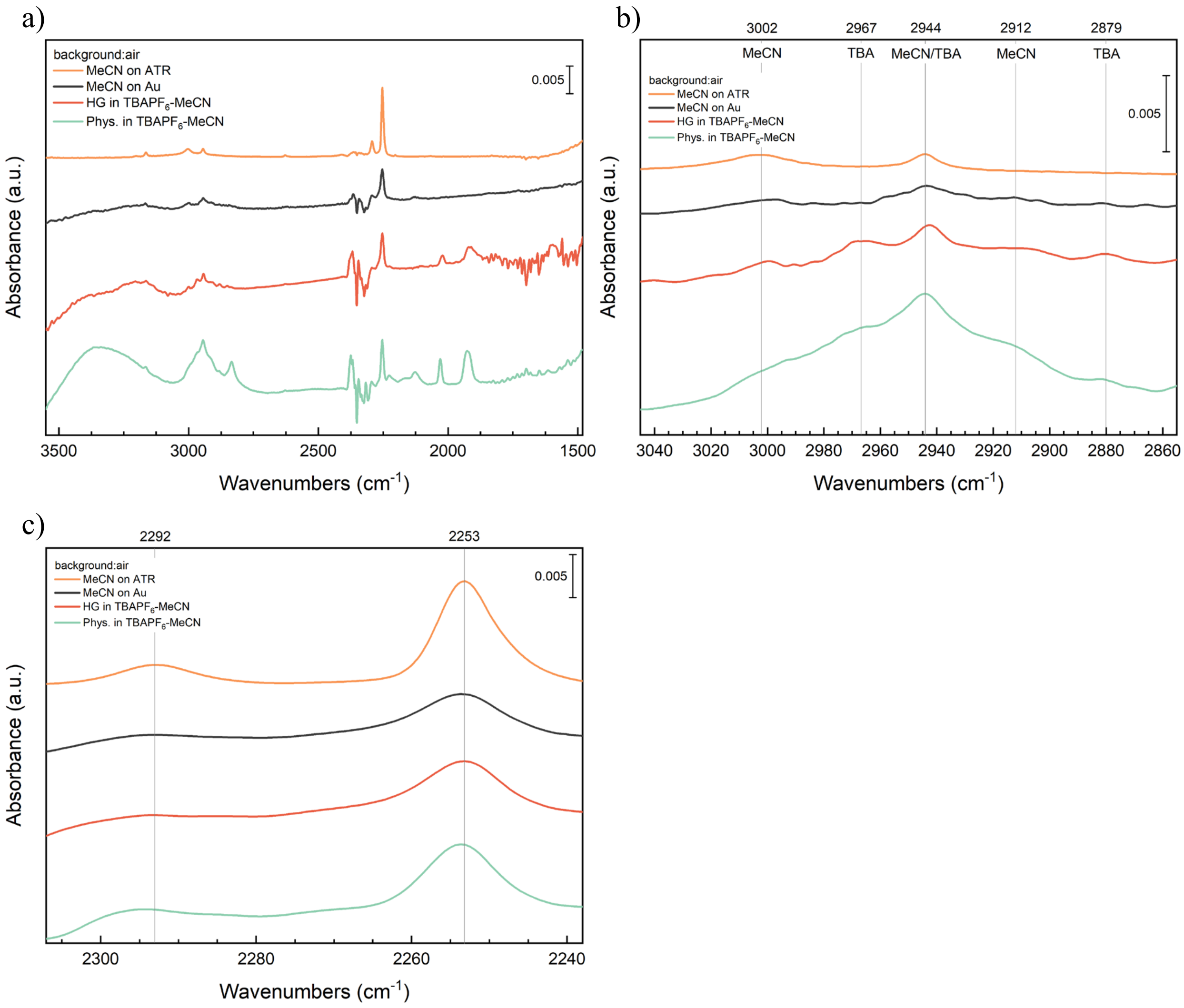

Figure S21. MeCN solvent features in the bulk detected by ATR-IR (orange line), SEIRAS (black lines) and SEIRAS in the presence of HG complexes (red lines) and physisorption (green lines). In (a), the entire spectral range is shown. In (b), a zoom in of the CH stretching modes from MeCN and TBA is shown.[26] In (c), a zoom in of the CN stretching and the combination of CC stretching and CH bending modes is shown. The observed solvent features are similar for physisorbed versus HG systems as well as versus the solvent features on bare Au, suggesting no clear change in interfacial solvent structure. In all spectra, a clean Au surface in air was used as the background.

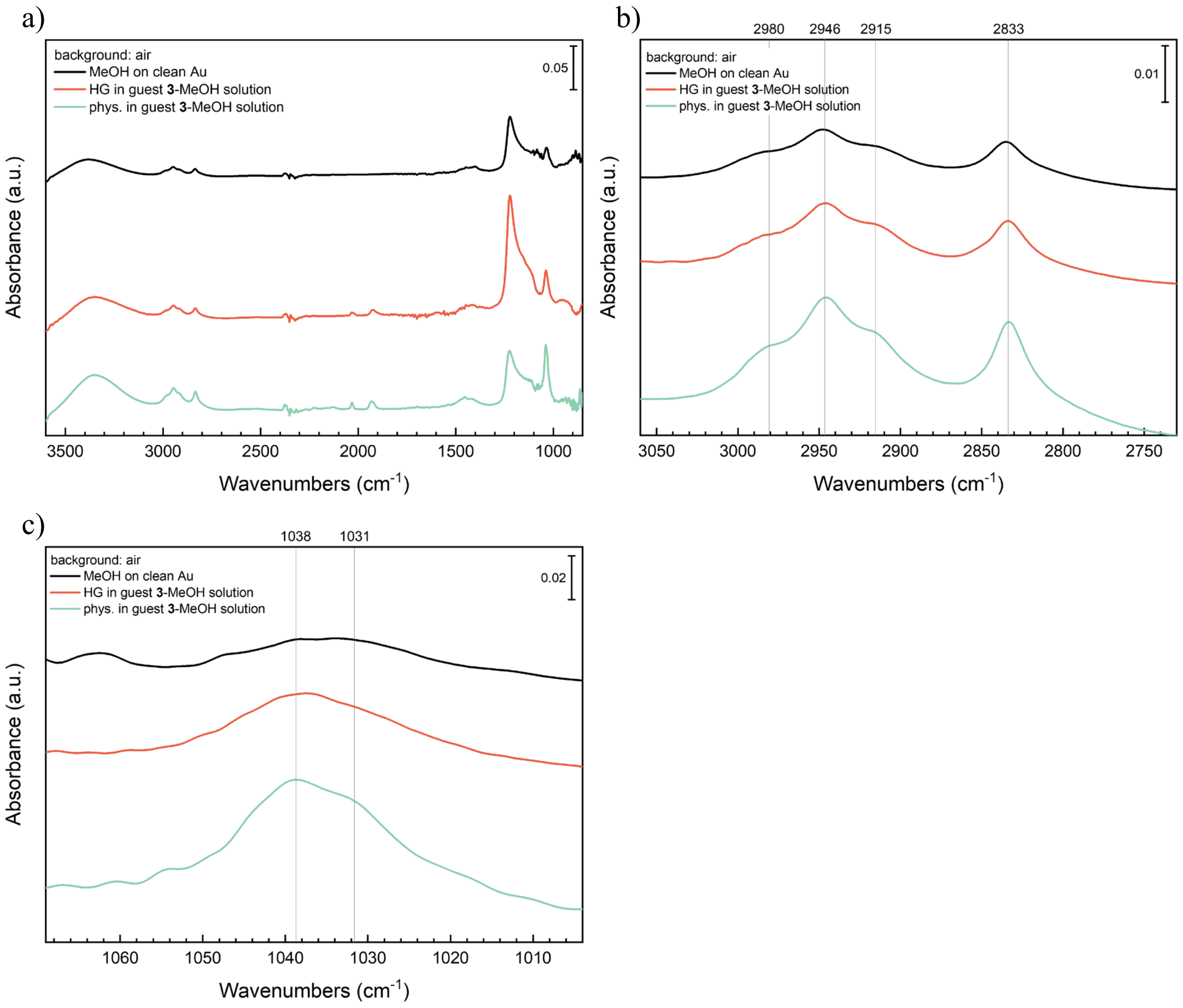

Figure S22. MeOH features detected by SEIRAS for clean Au (black lines), HG complexes on Au (red lines) and physisorption on Au (green lines). In (a), the entire spectral range is shown. In (b) and (c), a zoom in of the CH stretching and the CO stretching modes are shown, respectively. The observed solvent features are similar for physisorbed versus HG systems as well as versus the solvent features on bare Au, suggesting no clear change in interfacial solvent structure. In all spectra, a clean Au surface in air was used as the background.

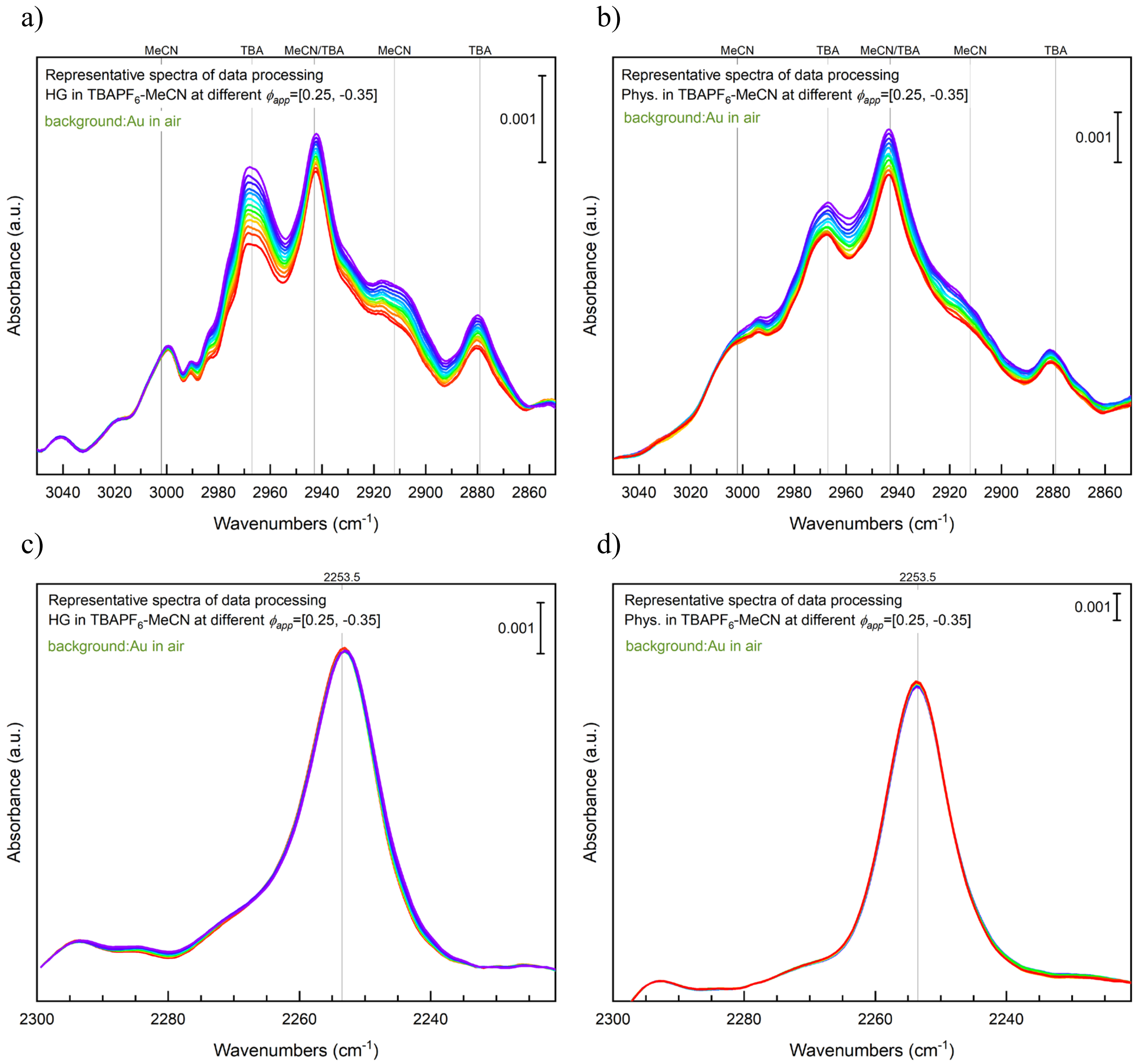

Figure S23. SEIRA spectra showing the bands associated with the $TBAPF_6$ in MeCN electrolyte[26] during an applied potential series for HG and physisorption. The CH stretching modes from TBA and MeCN for (a) HG and (b) physisorption. The CN stretching mode from MeCN for (c) HG and (d) physisorption. The observed changes in the band intensities and lack of change in wavenumbers with applied potential are similar for physisorbed versus HG systems showing no obvious differences in solvent structure across different systems. In all spectra, a clean Au surface in air was used as the background.

Physisorption A'(1) band comparison with varying guest 3 solution concentration

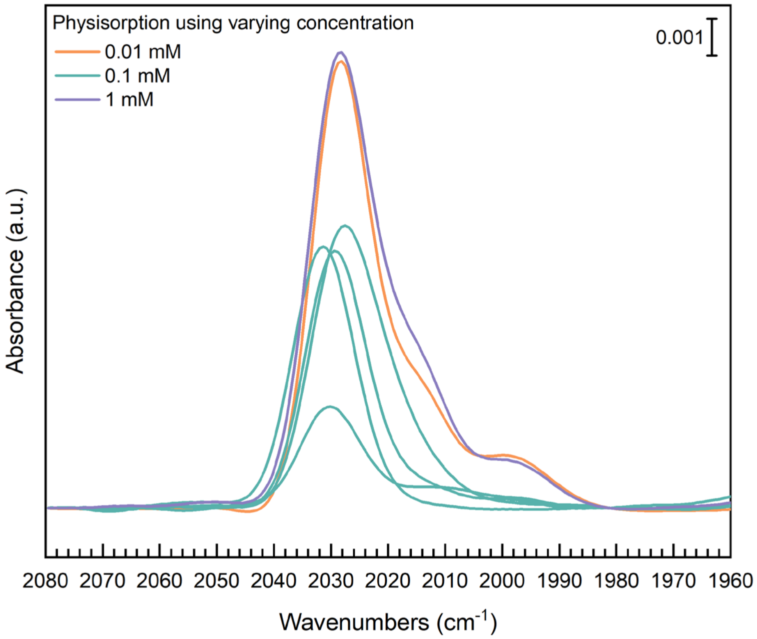

Figure S24. Six independent SEIRAS measurements of physisorption with varying guest **3** concentration, showing the A'(1) band. A clean Au surface in MeOH was used as the background spectrum in all cases. Spectra shown in green lines correspond to four independent SEIRAS replicate measurements shown in Figure S11.

Table S4. Integrated band area, FWHM and band position for the A'(1) band of physisorbed guest **3** at different concentrations. Values obtained from a Voigt fit of the spectra shown in Figure S24.

Guest 3 (*M*)	Area Intg.	FWHM	Center
0.01	0.14	11.4	2028.36
0.1	0.11	12.7	2028.85
0.1	0.11	12.6	2031.19
0.1	0.05	12.7	2029.59
0.1	0.14	15.7	2026.44
1	0.15	12.1	2028.44

Binding kinetics analysis

The comparison of HG and physisorption binding kinetics was done by fitting the plots of the integrated band area versus guest **3** immersion time (Figure S25) with a single exponential function (Eq. 2) to obtain the time constants. The single exponential fitting suggests that the binding follows 1st order Langmuir kinetics,[27,28] despite the fact that this model does not consider interacting sites

and multilayer formation. We therefore used Eq. 2 to estimate the effective timescale for comparison purposes.

$$A = A_0\left(1 - e^{-t/\tau}\right) \quad (2)$$

In Eq. 2, A is the integrated area of the A'(1) band at a given time t, A_0 is the asymptote and τ is the time constant. The average τ value for HG complexes and physisorption were 25.11 ± 3.59 min and 7.82 ± 0.47 min respectively (insets in Figure S25), which shows the rate for physisorption is ~3 times faster than HG complexation.

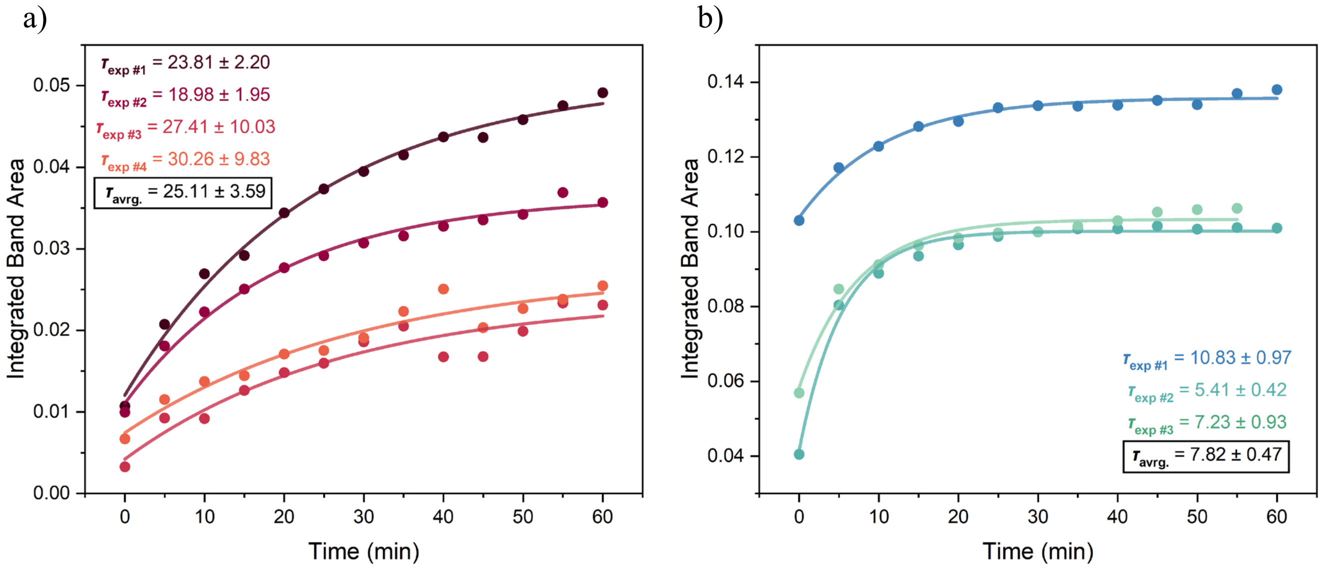

Figure S25. The integrated band area of A'(1) band as a function of guest **3** immersion time for (a) HG complexes and (b) physisorption. The different colors correspond to different independent replicate experiments. Each dataset is fitted with the Eq. 2 shown above, and the resulting exponential fits are shown as the solid lines. The insets show the τ values obtained from the exponential fit of each replicate and the calculated average. We note that the fitting of the data shown in blue circles in (b) was difficult due to the asymmetric band shape obtained in that particular measurement (Figure S11a). The fitting for that dataset was also done with 2 peaks, however, no clear trend was obtained in that case, therefore we used the 1 peak fitting data here.

Adsorption selectivity of guest 3 on Au(111)

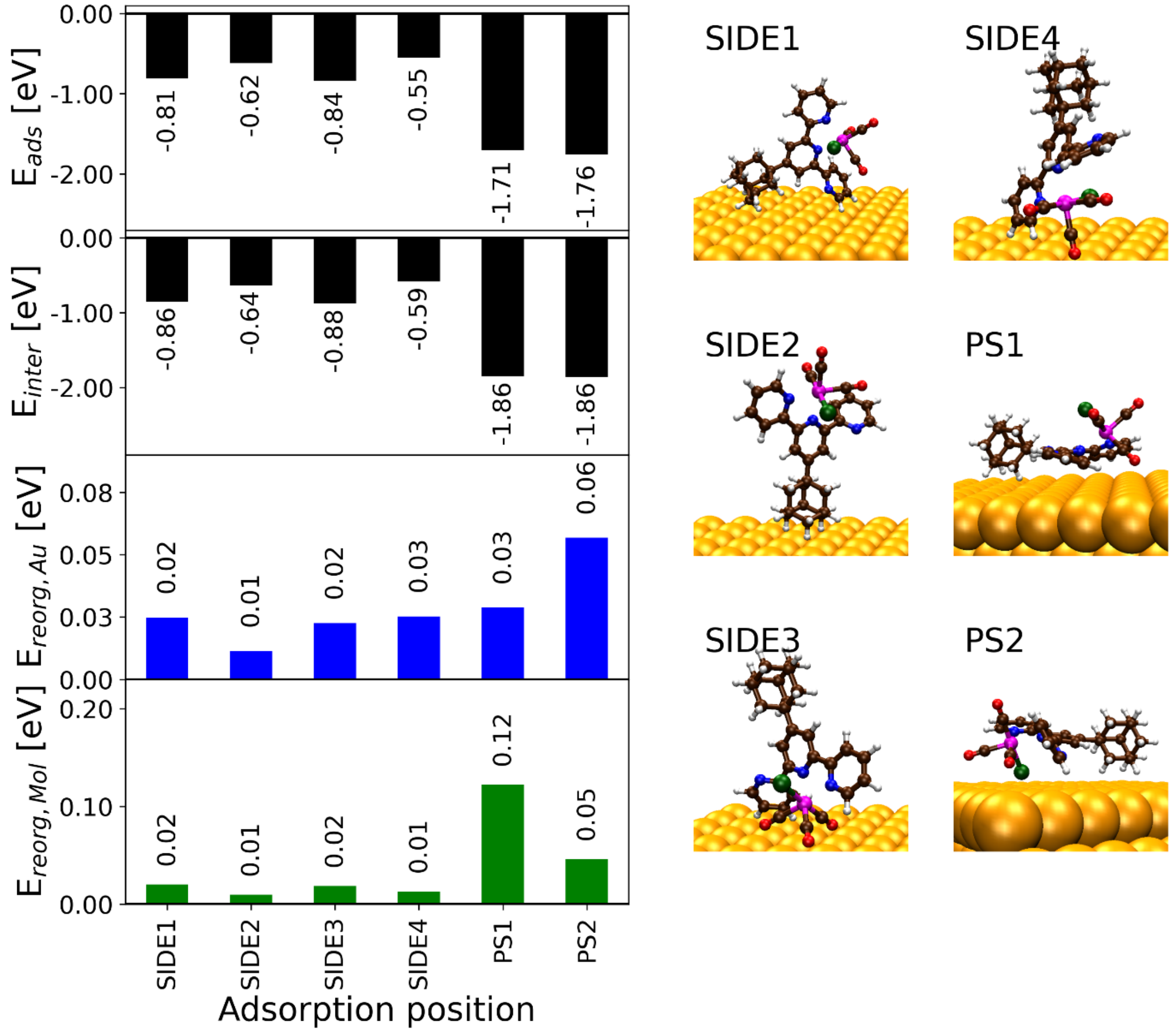

Figure S26. Adsorption, interaction and reorganization energies of guest **3** on Au(111) with the respective structures shown on the right. Au: gold, C: brown, Cl: green, H: white, N: blue, Re: magenta, O: red.

DFT calculations of guest **3** adsorption on Au(111) identified two stable configurations. As shown in Figure S26, horizontal adsorption on the clean gold surface yields strong adsorption energies of -1.71 eV and -1.76 eV for PS1 and PS2, respectively. These configurations are the most probable due to their significantly higher adsorption energies (≥0.98 eV) compared to perpendicular or angled orientations.

In these horizontal configurations, stronger interaction energies are observed, leading to an increase in the reorganization energies of both the molecule ($E_{reorg,Mol}$) and the surface ($E_{reorg,Au}$). Reorganization energies were determined as the energy difference between the isolated state of the molecule/surface (E_{iso}) and the adsorbed state (E_{opt}) from a single-point calculation using the geometry of the respective system. The results indicate that PS1 and PS2 exhibit similar interaction

energies, with differences in adsorption energy arising from variations in molecular and surface reorganization upon adsorption.

Adsorption selectivity of guest 3 in and around host 1

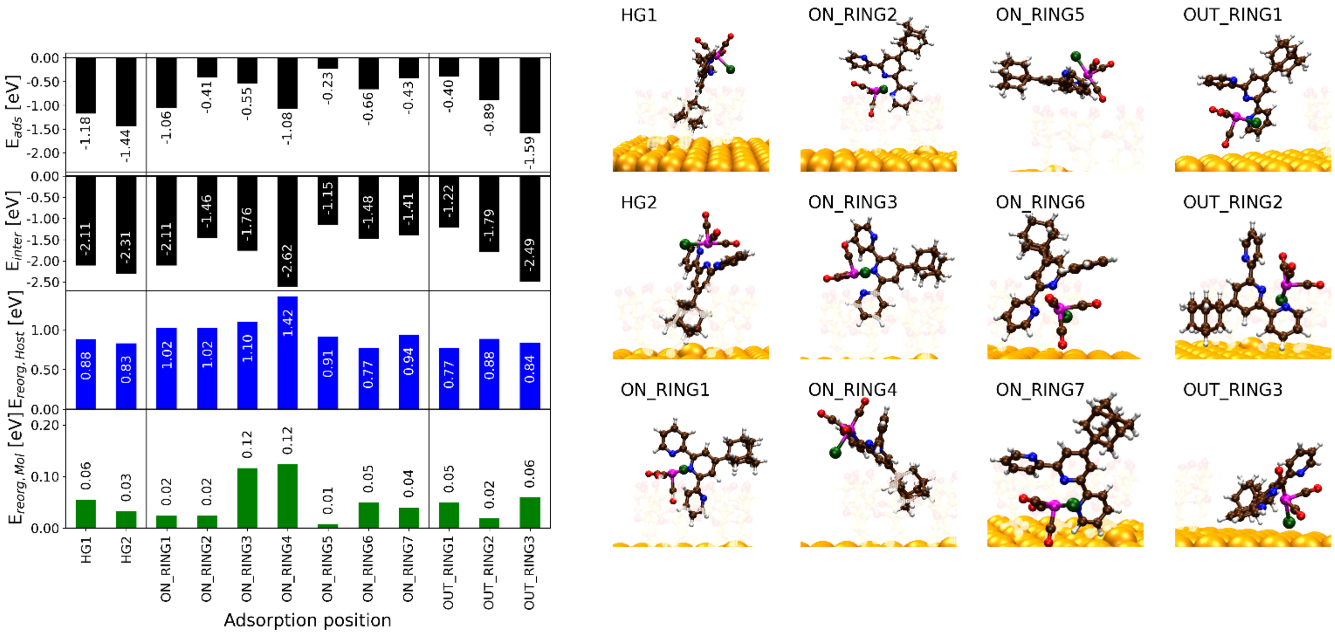

Figure S27. Adsorption, interaction and reorganization energies of guest **3** in and around the host 1 with the respective structures shown on the right. Au: gold, C: brown, Cl: green, H: white, N: blue, Re: magenta, O: red, S: yellow. The host **1** has been shaded to depict the adsorption of guest **3**.

DFT calculations of guest **3** adsorption via HG complexation identified two stable configurations, HG1 and HG2. As shown in Figure S27, HG2 is more stable than HG1 by 260 meV. This increased stability results from a 200 meV rise in interaction energy and a slight reduction in reorganization energy for both the host and the guest molecule. Consequently, this configuration reduces strain in the overall complex, enhancing stabilization. In our study, multiple in-ring configurations were tested, all of which relaxed into either HG1 or HG2 upon final optimization. The primary increase in adsorption energy is attributed to enhanced interaction energies, as the upright adsorption of HG2 provides a better structural fit compared to HG1. Compared to the most stable physisorption configurations, PS1 and PS2, the interaction energies (E_{inter}) of HG1 and HG2 were higher by 300-500 eV. However, due to the reorganization energy (E_{reorg}) that the host and the guest molecules need to invest to form the HG complex, the overall adsorption energies of the HG1 and HG2 were found to be smaller than the PS1 and PS2.

Beyond complexation, we investigated different adsorption configurations of guest **3** on and around the host **1** molecule. For on-ring adsorption, coordination via the free pyridine ring of the terpyridine ligand leads to a stable configuration in ON_RING1. Additionally, a fully horizontal adsorption results in ON_RING4, where the adamantane molecule falls into the host ring. However, this induces significant increase in the interaction energy and molecular strain in both the guest and host. Despite this, ON_RING4 is identified as a potential pre-state for HG1,

suggesting further rearrangement into the host cavity over time. Furthermore, the tested on-ring adsorption structures, ON_RING1 and ON_RING4 are identified as potential metastable states. All other configurations show no significant stabilization and are expected to transition into more stable states or undergo re-adsorption over time.

The adsorption of guest **3** outside the host ring (OUT_RING) was also examined, revealing three stable configurations. Among these, OUT_RING3 emerged as a competing candidate for guest **3** adsorption versus HG complexation. In this configuration, guest **3** adopts an almost horizontal orientation on the Au surface near the host ring. However, this structure is considered a variation of PS2, featuring weak coordination to the host ring.

Vibrational spectra of guest 3

Vibrational spectra were calculated by using the vibrational analysis tool implemented in CP2k. In the vibrational analysis only the atoms of the metal center and aligning atoms were allowed to relax. No significant changes were found in the positions of the chemical shift for the restraining of the other atoms compared to a non-restrained case. All spectra were computed for a threshold of ≤ 1x 10-8 Hartree for optimal accuracy of the vibrations.

The self-consistent continuum solvation model (SCCS)[29,30] was used for all the computations of vibrational spectra using implicit solvents. Using previous wave functions from PBE calculations in all cases, the Andreussi method[30] was employed for the calculations of the implicit solvent environment with the CD3 derivative method. The dielectric constants used were acetonitrile: 37.5, methanol: 32.7 and dimethyl sulfoxide: 46.8. Explicit solvent molecules were placed around the metal center of the guest complex using the PACKMOL package.[31] Structures were visualized using the VMD software. [32–38]

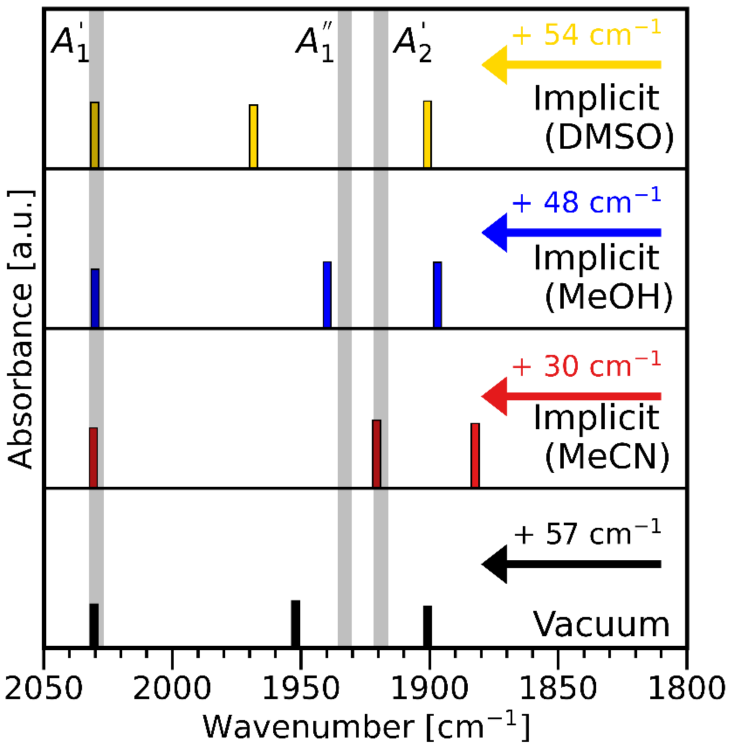

Figure S28. Vibrational spectra of guest **3** calculated by DFT calculations in vacuum and implicit solvents (MeCN, MeOH & DMSO). The bands were aligned according to the A'(1) mode with the respective shifts.

Table S5. Relative intensities of the vibrational bands calculated via DFT calculations in vacuum and implicit solvents (MeCN, MeOH & DMSO).

	**Wavenumbers (cm^{-1})**	**Shifted Wavenumbers (cm^{-1})**
**Vacuum**	1843.82	1900.82
	1895.07	1952.07
	1973.47	2030.47
**Implicit (MeCN)**	1852.24	1882.24
	1890.59	1920.59
	2000.70	2030.70
**Implicit (MeOH)**	1848.94	1896.94
	1891.81	1939.81

	1982.03	2030.03
**Implicit (DMSO)**	1846.82	1900.82
	1914.42	1968.42
	1976.21	2030.21

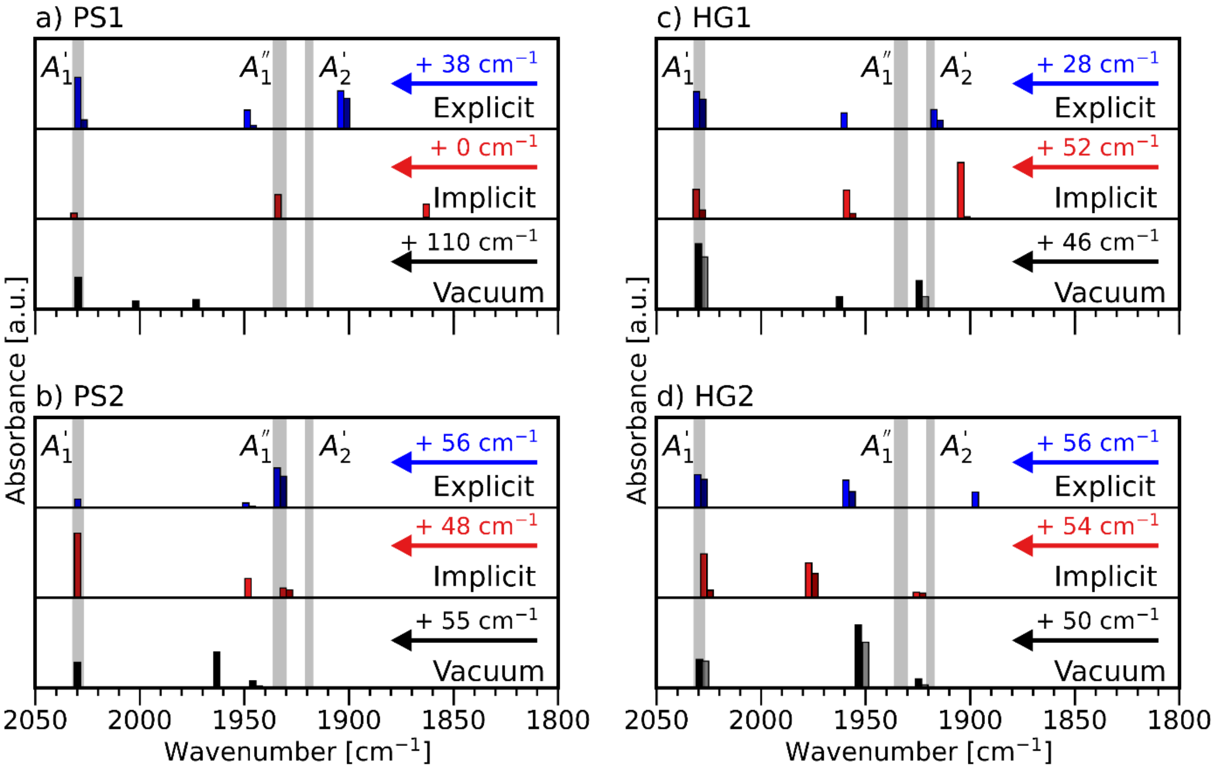

Figure S29. Calculated vibrational spectra of the CO vibrations of guest **3** (a & b) physiosorbed on Au(111) (PS) and (c & d) HG complex (HG) obtained from vacuum calculations, implicit acetonitrile (ε= 37.5) and explicit acetonitrile. The signals were shifted with respect to the A'(1) vibration of the CO -ligands. The black red and blue bars indicate the total intensity of the vibration, while the dark red, dark blue and grey bars represent the part of the z-component of this vibration. The experimental range of the vibrations is indicated via the grey shaded areas.

Table S6. Relative intensities of the vibrational peaks calculated via DFT calculations in vacuum, implicit and explicit solvent. I: Scaled total intensity with respect to the highest peak set as 1.00. I_z: z-Component of the peak scaled to the highest intensity set to 1.00. $I_{z,rel}$: Relation of the z-component as part of the total intensity of the peak.

		Wavenumbers (cm^{-1})	Shifted Wavenumbers (cm^{-1})	Total Intensity, *I* (a.u.)	z-Intensity, I_z (a.u.)	$I_{z,rel}$
**PS1**	*Vacuum*	1863.1	1973.1	0.29	0.11	1.16%
		1891.92	2001.92	0.26	1	12.01%
		1921.47	2029.47	1	0.01	0.03%
	Implicit	1863.06	1863.06	0.6	1	12.28%
		1933.91	1933.91	1	0.24	1.74%
		2031.45	2031.45	0.23	0.32	10.31%
	Explicit	1860.95	1903.95	0.73	1	80.35%
		1913.66	1948.66	0.36	0.11	18.14%
		1991.58	2029.58	1	0.29	17.25%
**PS2**	*Vacuum*	1890.94	1945.94	0.29	0.11	28.22%
		1906.19	1963.19	1	0.47	2.49%
		1974.79	2029.79	0.71	0.07	0.48%
	Implicit	1889.42	1931.42	0.15	1	78.83%
		1991.24	1948.27	0.3	0	0.09%
		1977.76	2029.76	1	0	0.01%
	Explicit	1878.34	1934.34	1	1	79.01%
		1893.3	1949.3	0.11	0.04	25.54%
		1973.69	2029.69	0.21	0.01	3.01%
**HG1**	*Vacuum*	1878.39	1924.39	0.44	0.24	43.50%

		1916.49	1962.49	0.19	0.01	2.60%
		1981.97	2029.97	1	1	79.90%
	Implicit	1852.68	1904.68	1	0.24	3.82%
		1907.24	1959.24	0.51	0.59	18.66%
		1978.9	2030.9	0.52	1	30.85%
	Explicit	1889.35	1917.35	0.52	0.28	43.70%
		1932.3	1960.3	0.43	0.01	2.00%
		2002.85	2030.35	1	1	80.00%
**HG2**	*Vacuum*	1871.71	1924.71	0.14	0.06	31.80%
		1902.02	1953.02	1	1	72.40%
		1976.94	2029.94	0.45	0.59	94.00%
	Implicit	1871.72	1925.72	0.12	0.18	84.76%
		1923.23	1977.23	0.79	1	69.52%
		1974.37	2028.37	1	0.3	16.35%
	Explicit	1841.55	1897.55	0.47	0	0.20%
		1903.43	1959.43	0.83	0.56	57.40%
		1974.3	2030.3	1	1	85.10%

Figure S29 represents the vibrational calculations for the two most stable configurations of the physisorbed states (PS) and host-guest complex (HG), evaluated in vacuum, implicit, and explicit acetonitrile using clusters of 42 to 47 acetonitrile molecules around the metal center. The accuracy of these models was assessed by comparing their vibrational frequencies with experimental data. Additionally, we explored a cluster approach, combining an extended explicit model with implicit solvent effects. However, due to its high computational cost, this method was not broadly applied to our system. The results from the cluster approach showed only minor shifts in vibrational frequencies compared to the explicit solvent model. Consequently, the marginal improvement in accuracy did not justify the significant computational expense.

Based on the results for the PS states, DFT calculations using implicit solvents show the best overall agreement with experimental data. Both PS1 and PS2 exhibit strong alignment of A'(1) vibrations with experiment, with PS1 achieving near-perfect overlap without signal shifts. Despite

their similar adsorption energies, PS2 is slightly more stable. However, greater stability corresponds to a reduction in molecular reorganization energy, which decreases from 0.12 eV in PS1 to 0.05 eV in PS2. The stronger distortion of the complex in PS1 increases molecular strain, leading to changes in dipole moment and, consequently, vibrational shifts.

For HG complexes, both structures exhibit similar behavior across all computational models. While vacuum calculations provide the best agreement with experimental results in both cases, both implicit and explicit solvation models generally reproduce the observed trends. Our calculations yielded the total vibrational intensity (I) and the corresponding z-component (I_z). As shown in Table S6, different solvation models do not reveal a clear trend in intensities or z-components. For proper comparison, the highest intensity in each model was normalized to 1.00. The ratio $I_{z,rel}$ was computed to compare the fraction of the z-component relative to the total intensity for each vibration. In general, the use of implicit solvents reveals a more consistent picture while being computationally demanding with respect to the explicit approach. However, the use of explicit solvent molecules affects the electronic structure of the ligands and hence lead to a change in the dipole moment.

The mismatch between calculated and experimental intensity ratios is associated with the lack of kinetic energy in the calculations. As a result, the computed values represent only a static snapshot of the system at a given moment. Since SEIRA intensity follows $I \propto \cos2(\theta)$, where θ is the angle of the molecule relative to the surface, the observed signal is influenced by the vibrational amplitude at this position. This effect significantly alters the intensity ratio, as more perpendicular vibrations with respect to the surface (lower θ) contribute more strongly to the overall intensity.

Distance analysis of guest 3 for most stable configurations of physisorption & HG

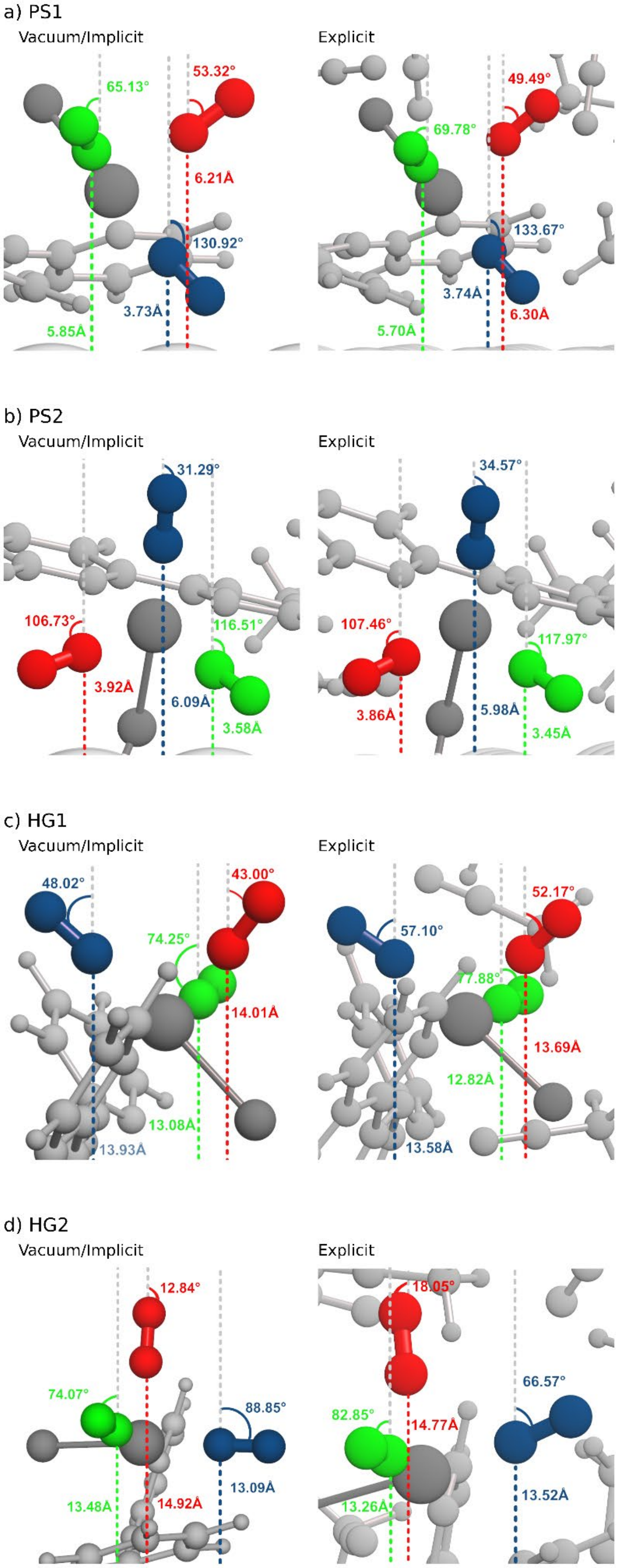

Figure S30. Distances and angles of the guest **3** CO's to Au(111) surface for (a) PS1, (b) PS2, (c) HG1 & (d) HG2 configurations obtained from DFT calculations. The analysis is shown in vacuum and explicit acetonitrile molecules around the metal center. The CO ligands have been highlighted in red, blue and green. The blue CO denotes the trans-CO ligands, while red and green denote the cis-ligands. The Re metal center and Cl ligand are colored in dark grey. The rest of the guest molecule, host **1** and residual solvent molecules were colored in light grey.

Projected density of state (PDOS) analysis

The projected density of states (PDOS) of the most stable configurations of the physisorption and the HG are displayed in Figures S31 to S34. All PDOS plots were aligned with the HOMO state of the Re metal center. In order to provide a more in depth analysis, we first calculated the PDOS of the most stable HG or physisorption configurations on Au. We then sequentially removed the Au layer – and in the HG case, the host as well – while keeping the configurations fixed. This provided the PDOS of guest **3** (and HG) in vacuum, but in the same geometries it adopts upon adsorption on Au. By comparing these results to the PDOS obtained of optimized guest **3** structure in vacuum, we were able to demonstrate the effect of configurational changes on the guest **3** states. Consequently, this comparison also allowed us to identify the electronic contributions that might arise from interactions with Au and/or the host molecule.

For PS2, we observe Cl-ligand coordination with the Au surface, leading to states at ~0.5 eV and 1.5 eV (Figure S32c). Similarly, in PS1, coordination occurs primarily via N atoms, with minor contributions from O atoms (Figure S31c). Both configurations reveal an increase of the Au states in the range of 0-2.0 eV, indicating interactions of the guest **3** and the formation of new states. This aligns with Figure S26, where the uncoordinated pyridine group of terpyridine faces the surface alongside contributions from one of the CO ligands.

For the HG1 (Figure S33) and HG2 (Figure S34) configurations, we observed that while the main states of guest **3** remain widely unchanged in the deformed configurations (Figures 33b & 34b), the interaction with host **1** leads to a significant change in the electronic structure of guest **3**. For instance, the second major signal of the Re states show a shift from ~ –0.7 eV to –0.4 eV upon HG complexation. Furthermore, the HG complexation in the absence of the Au surface, results in a slight increase in the molecular gap by 0.2 to 0.3 eV (Figures S33c & S34c). This gap is slightly decreased again for the host coordination on Au(111) (Figures S33d & S34d), while still remaining larger than in the isolated guest **3** state (Figures S33a & S34a). Additionally, adsorption of the HG complex on Au leads to formation of new host states between 0-2.0 eV (Figures S33d & S34d), originating from S-Au bonds, showing a strong interaction between the host **1** and the Au surface.

PS1

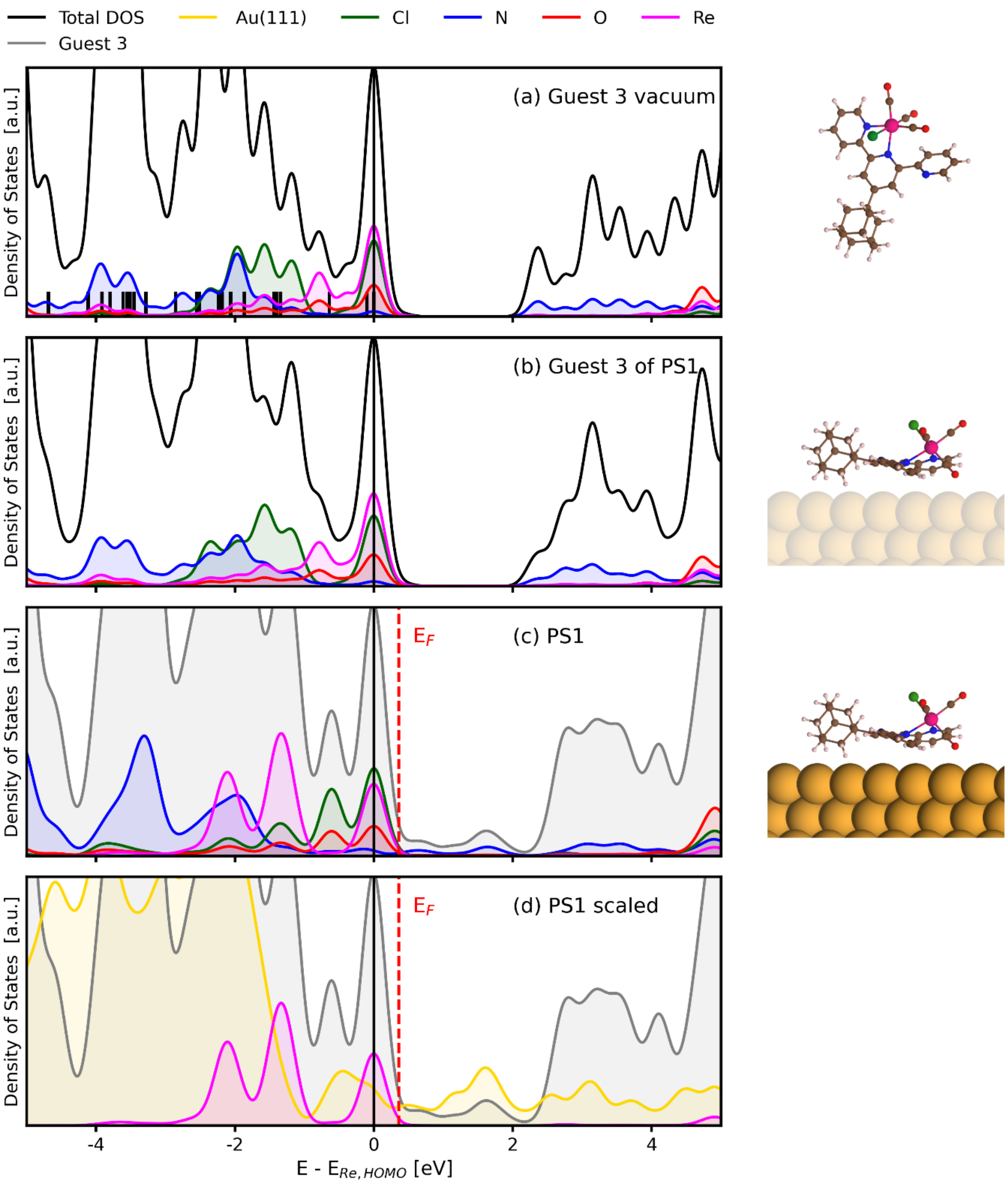

Figure S31. PDOS comparison for the physisorbed guest **3** on Au(111) in the PS1 configuration, calculated using the HSE06 functional. All plots are aligned with the Re HOMO level. (a) PDOS of the isolated guest **3** in vacuum.(b) PDOS of guest **3** in PS1 configuration without the Au(111). (c) PDOS of the full PS1 configuration including the Au(111) surface. (d) Scaled PDOS of the full PS1 configuration showing the Au-guest **3** interaction. The PDOS of Au(111) was decreased by a factors of 80. The red dashed lines in (c) and (d) show the Fermi level of the Au.

PS2

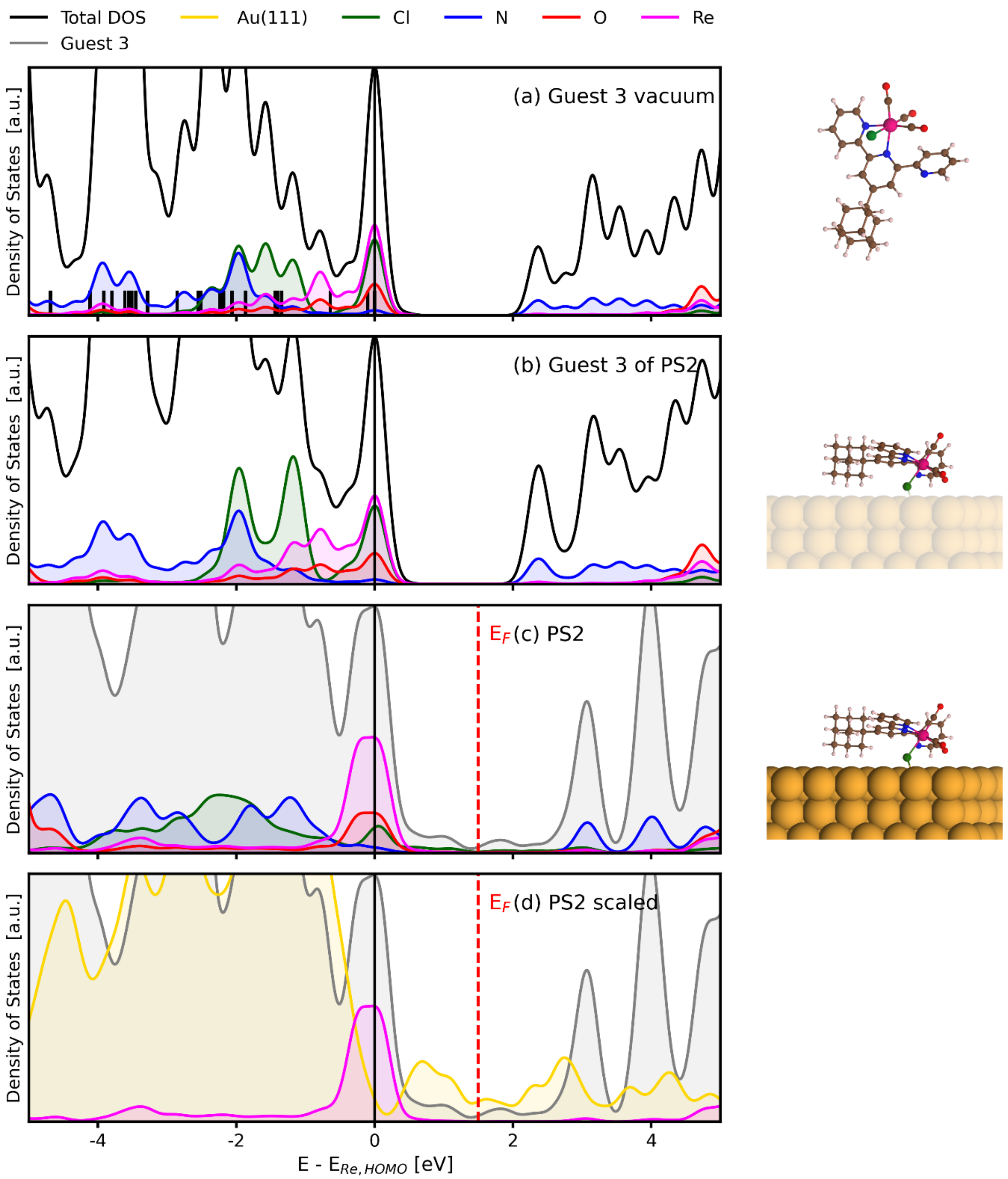

Figure S32. PDOS comparison for the physisorbed guest **3** on Au(111) in the PS2 configuration, calculated using the HSE06 functional. All plots are aligned with the Re HOMO level. (a) PDOS of the isolated guest **3** in vacuum.(b) PDOS of guest **3** in PS2 configuration without the Au(111). (c) PDOS of the full PS2 configuration including the Au(111) surface. (d) Scaled PDOS of the full PS2 configuration showing the Au-guest **3** interaction. The PDOS of Au(111) was decreased by a factors of 80. The red dashed lines in (c) and (d) show the Fermi level of the Au.

HG1

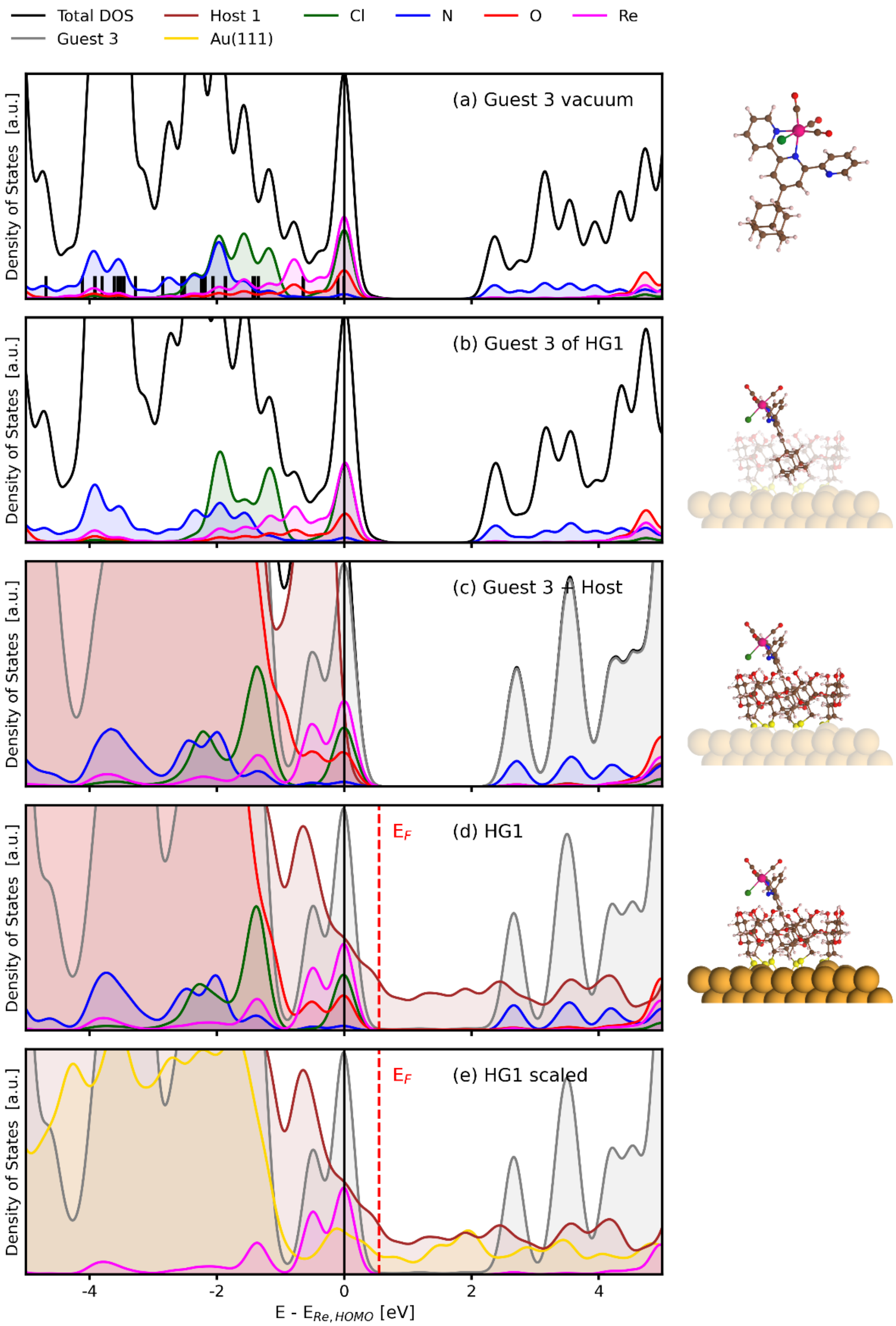

Total DOS
Host 1
Cl
N
O
Re
Guest 3
Au(111)
(a) Guest 3 vacuum
(b) Guest 3 of HG1
(c) Guest 3 + Host
(d) HG1
(e) HG1 scaled
E_F
Density of States [a.u.]
E - $E_{Re,HOMO}$ [eV]
-4
-2
0
2
4

Figure S33. PDOS comparison for the HG complex on Au(111) in the HG1 configuration, calculated using the HSE06 functional. All plots are aligned with the Re HOMO level. (a) PDOS of the isolated guest **3** in vacuum.(b) PDOS of guest **3** in HG1 configuration without the Au(111) and host **1**. (c) PDOS of the HG complex in HG1 configuration without the Au(111). (d) PDOS of the full HG1 configuration including the Au(111) surface. (e) Scaled PDOS of the full HG1 configuration showing the Au-host **1** interaction. The PDOS of Au(111) was decreased by a factors of 80. The red dashed lines in (d) and (e) show the Fermi level of the Au.

HG2

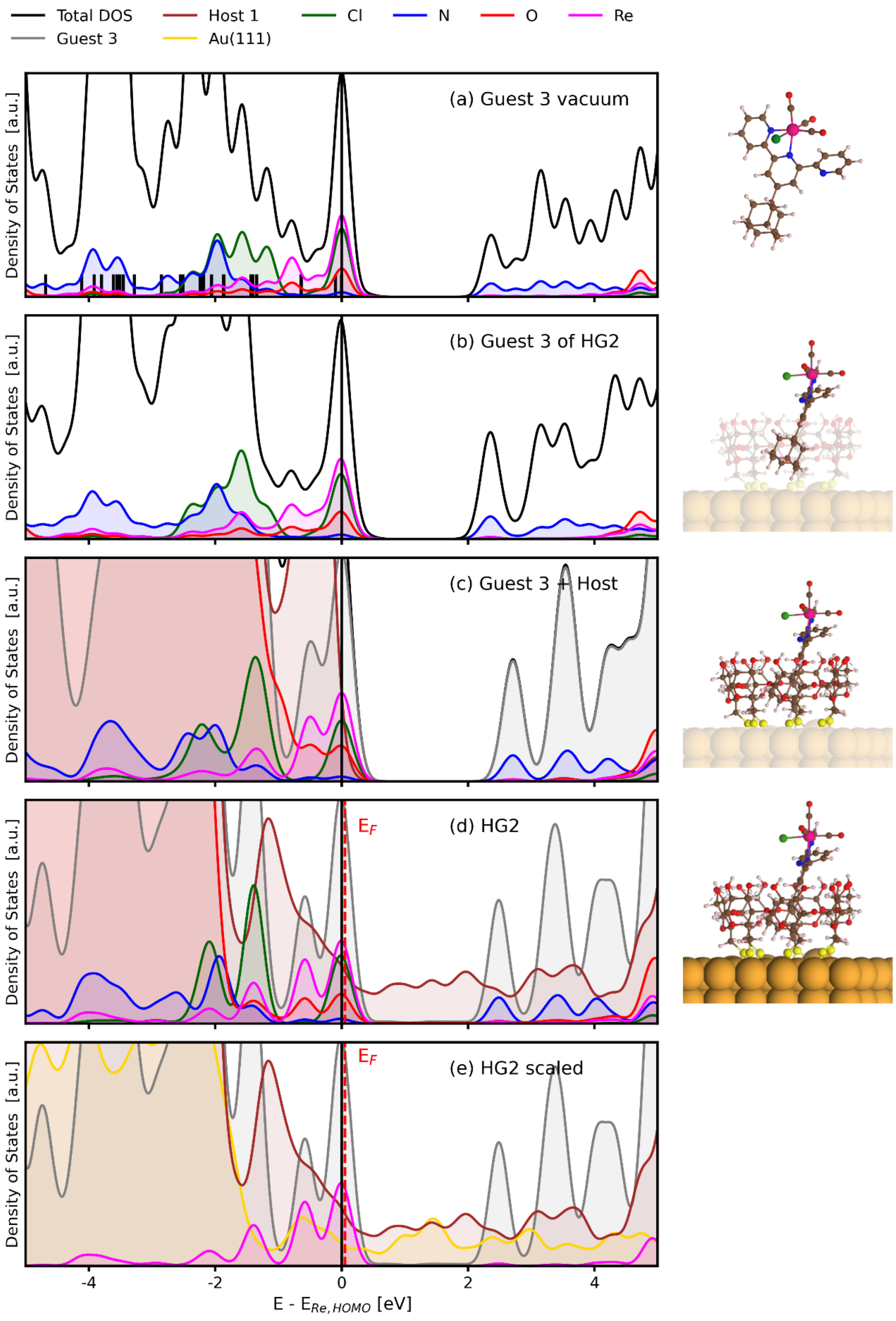
Total DOS
Host 1
Cl
N
O
Re
Guest 3
Au(111)
(a) Guest 3 vacuum
(b) Guest 3 of HG2
(c) Guest 3 + Host
(d) HG2
(e) HG2 scaled
E_F
Density of States [a.u.]
-4
-2
0
2
4
E - $E_{Re,HOMO}$ [eV]

Figure S34. PDOS comparison for the HG complex on Au(111) in the HG2 configuration, calculated using the HSE06 functional. All plots are aligned with the Re HOMO level. (a) PDOS of the isolated guest **3** in vacuum.(b) PDOS of guest **3** in HG2 configuration without the Au(111) and host **1**. (c) PDOS of the HG complex in HG2 configuration without the Au(111). (d) PDOS of the full HG2 configuration including the Au(111) surface. (e) Scaled PDOS of the full HG2 configuration showing the Au-host **1** interactions. The PDOS of Au(111) was decreased by a factors of 80. The red dashed lines in (d) and (e) show the Fermi level of the Au.

Cyclic voltammograms of Au with adsorbed guest 3

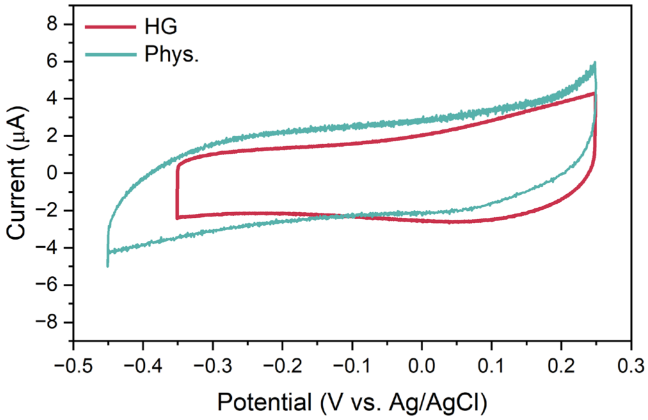

Figure S35. Representative cyclic voltammograms (CVs) of physisorbed (blue line) and host-bound (red line) guest **3** on Au. CVs were measured in 0.2 M $TBAPF_6$ in MeCN at a scan rate of 100 mV/s. No Faradaic waves were observed within the selected potential window in both cases.

Examples of SEIRA spectra showing the $\tilde{\nu}_{CO}$ bands during the applied potential

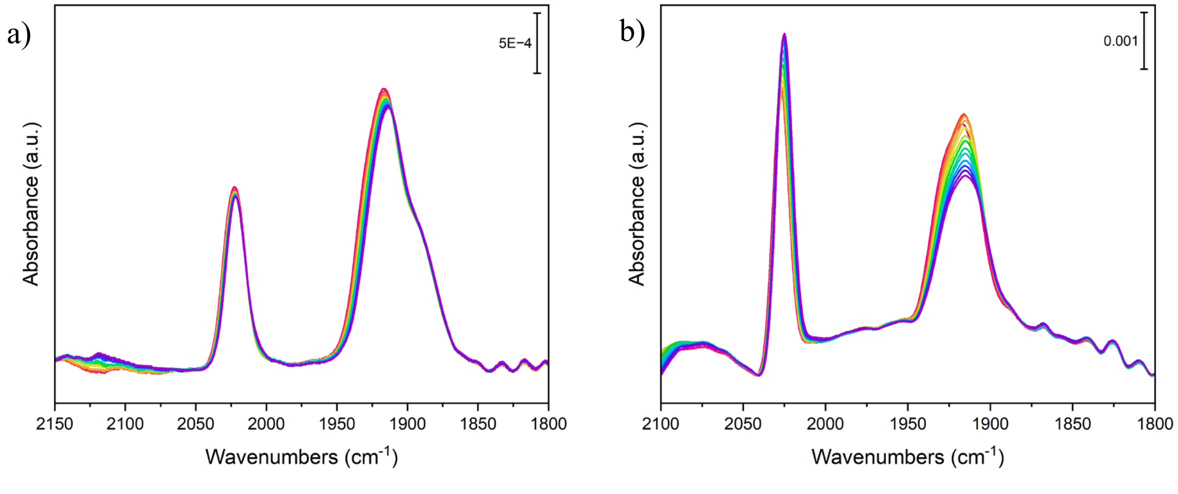

Figure S36. Representative examples of (a) HG and (b) physisorption SEIRA spectra recorded during the applied potential series, using either (a) host **1** functionalized or (b) clean Au in MeCN as the background spectrum. Rainbow colored lines denote the SEIRA spectra obtained from 0.25 V (pink) to -0.35 V (purple). In both cases, no additional band growth at higher or lower wavenumbers were observed, which shows that guest **3** is not going through a redox process at the selected potential window.

SEIRAS replicate measurements of HG complexes as a function of applied potential

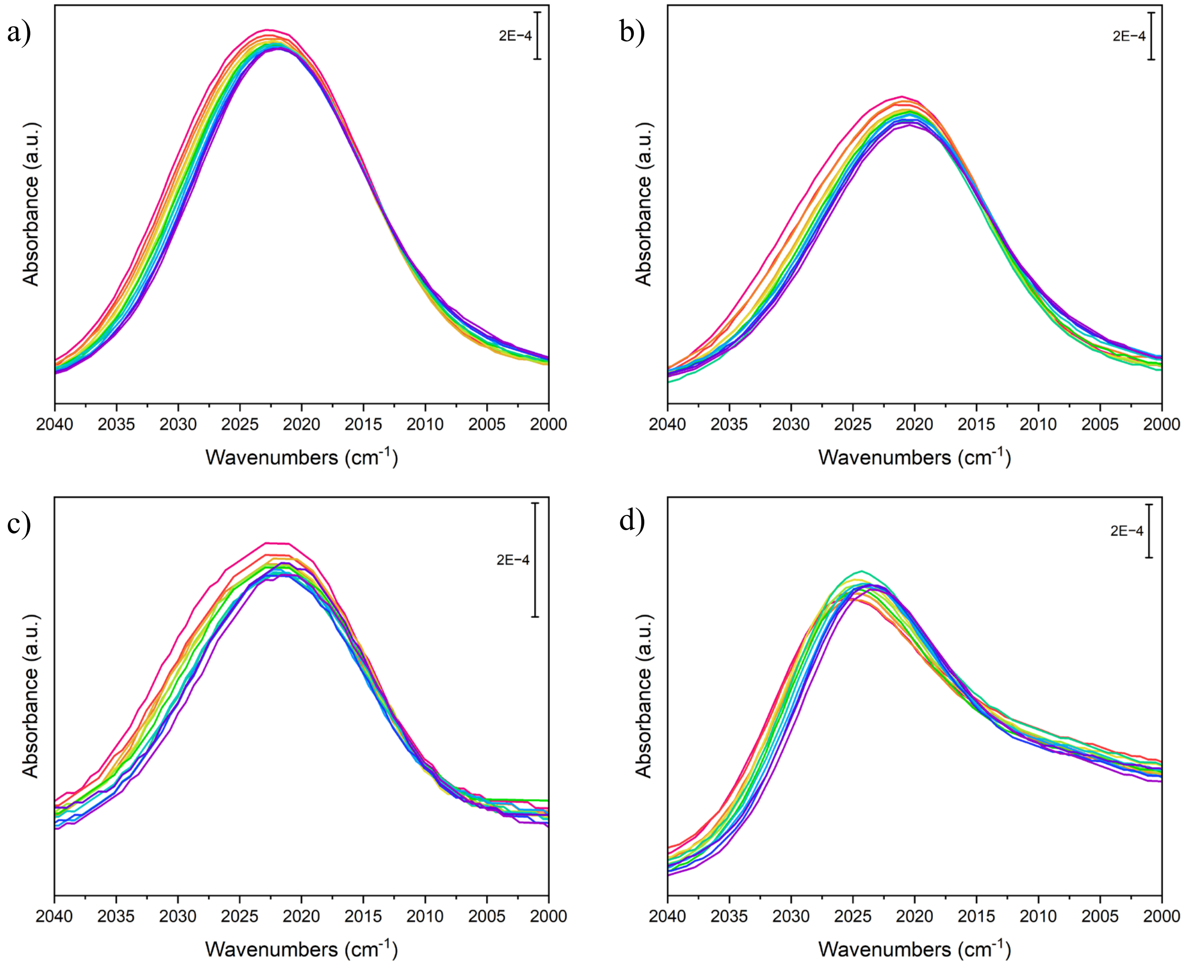

Figure S37. Three replicate experiments of SEIRAS measurements of Au bound HG complexes, showing the A'(1) band. (a) & (b) are obtained from the same experiment where the same potential window was covered twice. For all spectra, the host **1** functionalized Au surface in MeCN was used as the background spectrum, with the exception of (d), where the spectrum of clean Au in MeOH was used. Rainbow colored lines denote the SEIRA spectra obtained from 0.25 V (pink) to -0.35 V (purple). Ag/AgCl reference electrode prepared in 0.2 M $TBAPF_6$ in MeCN, was used for the measurement shown in (d).

SEIRAS replicate measurements of HG complexes as a function of applied potential (reverse scans)

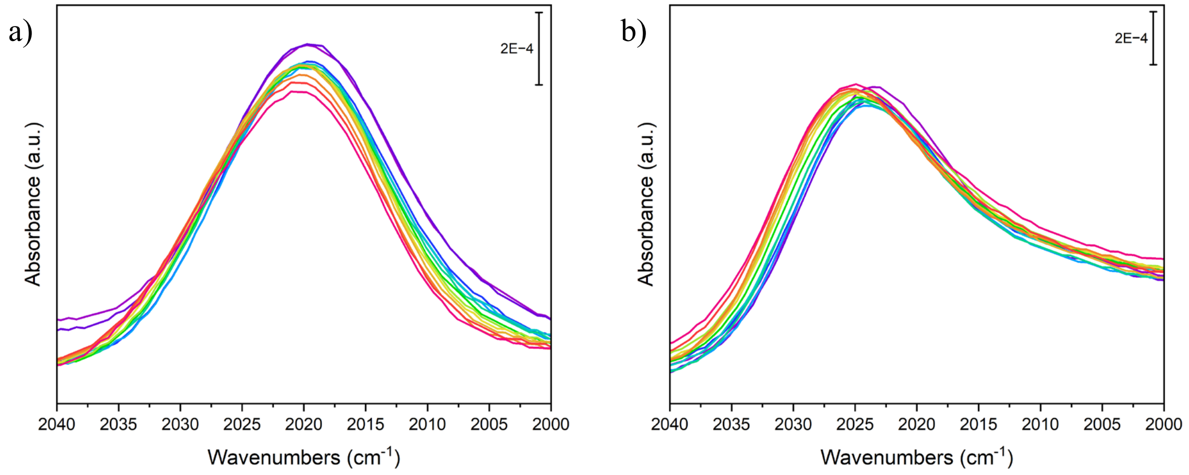

Figure S38. Two replicate experiments of SEIRAS measurements of Au bound HG complexes, showing the A'(1) band. For (a), the host **1** functionalized Au surface in MeCN was used as the background spectrum, and for (b), the spectrum of clean Au in MeOH was used. Rainbow colored lines denote the SEIRA spectra obtained from -0.35 V (purple) to 0.25 V (pink). Ag/AgCl reference electrode prepared in 0.2 M $TBAPF_6$ in MeCN, was used for the measurement shown in (b).

SEIRAS replicate measurements of physisorption as a function of applied potential

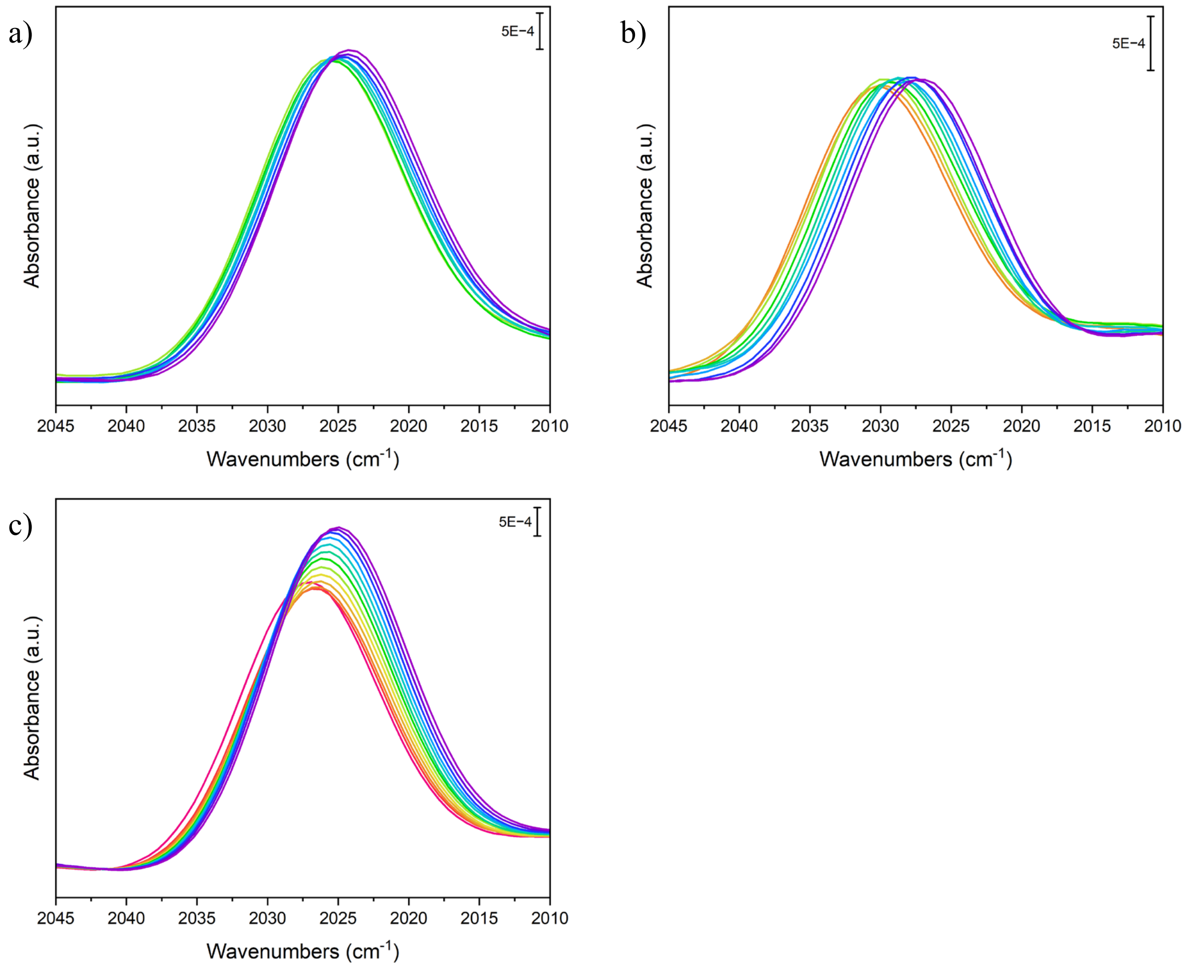

Figure S39. Three replicate experiments of SEIRAS measurements of physisorbed guests on Au, showing the A'(1) band. For all spectra, the clean Au surface in MeCN was used as the background spectrum, with the exception of (c), where the spectrum of clean Au in MeOH was used. Rainbow colored lines denote the SEIRA spectra obtained from (a) 0 V (light green) to -0.35 V (purple), (b) 0 V (orange) to -0.45 V (purple) & (c) 0.27 V (pink) to -0.33 V (purple). Ag/AgCl reference electrode prepared in 0.2 M $TBAPF_6$ in MeCN, was used for the measurement shown in (c).

SEIRAS replicate measurements of physisorption as a function of applied potential (reverse scans)

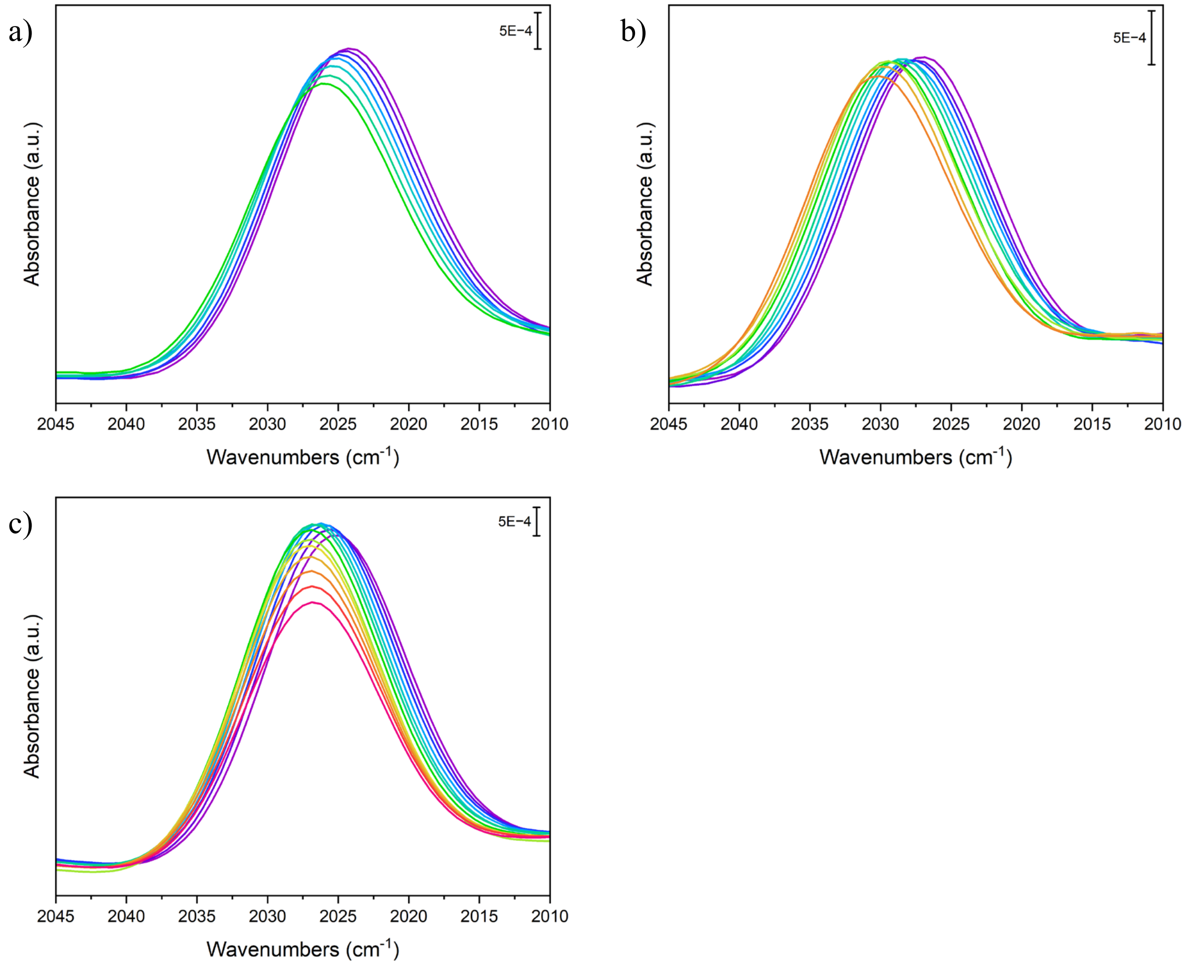

Figure S40. Three replicate experiments of SEIRAS measurements of physisorbed guests on Au, showing the A'(1) band. For all spectra, the clean Au surface in MeCN was used as the background spectrum, with the exception of (c), where the spectrum of clean Au in MeOH was used. Rainbow colored lines denote the SEIRA spectra obtained from (a) -0.35 V (purple) to -0.05 V (green), (b) -0.45 V (purple) to 0 V (orange) & (c) -0.33 V (purple) to 0.27 V (pink). Ag/AgCl reference electrode prepared in 0.2 M $TBAPF_6$ in MeCN, was used for the measurement shown in (c).

SEIRAS measurement of HG complexes as a function of applied potential at 1 M $TBAPF_6$ concentration

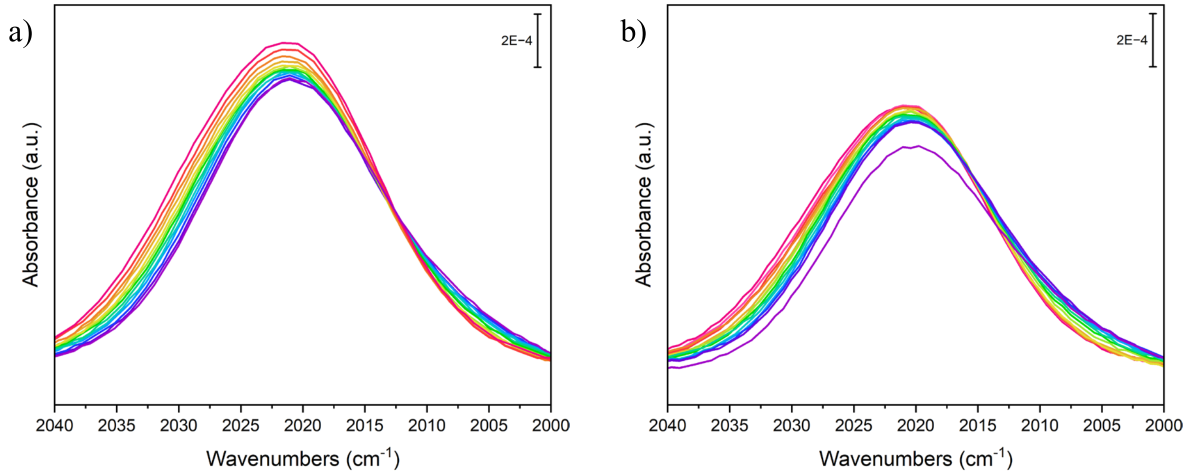

Figure S41. SEIRAS measurement of Au bound HG complexes, showing the A'(1) band as a function of applied potential. Host **1** functionalized Au surface in MeCN was used as the background spectrum. Rainbow colored lines denote the SEIRA spectra obtained from 0.25 V (pink) to -0.35 V (a, purple) or -0.40 V (b, purple). A baseline shift was observed after the measurements shown in (a) and (b) as we went to more negative applied potentials and then reversed to more positive applied potentials. The data with baseline shifts were hence not fitted. Nonetheless, reversibility obtained on a first scan to positive applied potentials (not shown in this figure) and the reproducible HG formation process shows the reliability of these measurements.

A'(1) band shifts as a function of applied potential for the HG complex at 1 M $TBAPF_6$ concentration

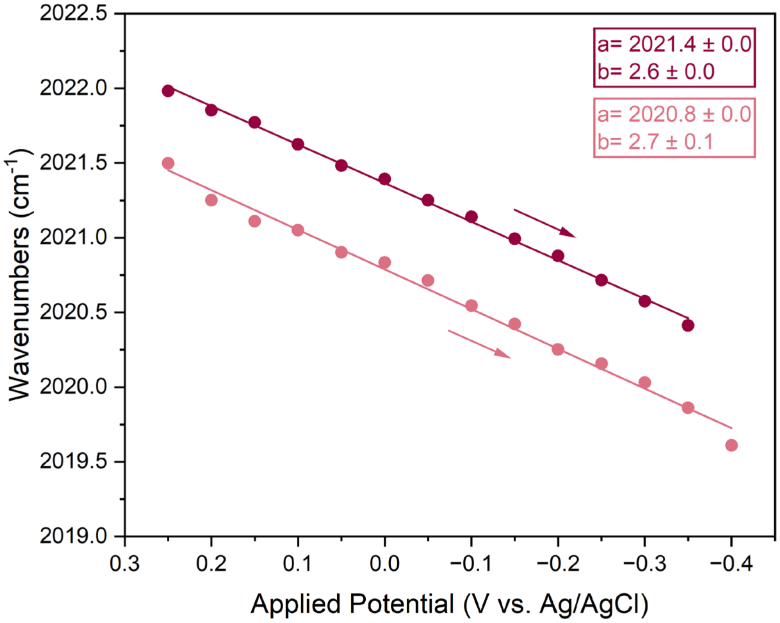

Figure S42. Plot of A'(1) band positions, obtained from Voigt fittings of the data shown in Figure S41, as a function of applied potential for the HG complex. Arrows show the direction of the applied potential. Linear fits of the A'(1) band position vs applied potential with intercept a and slope b for both sweeps are shown in color coded insets.

References

(1) Roithmeyer, H.; Séverу, L.; Moehl, T.; Spingler, B.; Blacque, O.; Fox, T.; Iannuzzi, M.; Tilley, S. D. Electrocatalytic Ammonia Oxidation with a Tailored Molecular Catalyst Heterogenized via Surface Host–Guest Complexation. *J. Am. Chem. Soc.* **2024**, *146* (1), 430–436. https://doi.org/10.1021/jacs.3c09725.

(2) Clark, R. C.; Reid, J. S. The Analytical Calculation of Absorption in Multifaceted Crystals. *Acta Cryst A* **1995**, *51* (6), 887–897. https://doi.org/10.1107/S0108767395007367.

(3) CrysAlisPro (Version 1.171.43.117a), Rigaku Oxford Diffraction Ltd, Yarnton, Oxfordshire, England, 2024.

(4) Dolomanov, O. V.; Bourhis, L. J.; Gildea, R. J.; Howard, J. a. K.; Puschmann, H. OLEX2: A Complete Structure Solution, Refinement and Analysis Program. *J Appl Cryst* **2009**, *42* (2), 339–341. https://doi.org/10.1107/S0021889808042726.

(5) Sheldrick, G. M. *SHELXT* – Integrated Space-Group and Crystal-Structure Determination. *Acta Crystallogr A Found Adv* **2015**, *71* (1), 3–8. https://doi.org/10.1107/S2053273314026370.

(6) Sheldrick, G. M. Crystal Structure Refinement with SHELXL. *Acta Cryst C* **2015**, *71* (1), 3–8. https://doi.org/10.1107/S2053229614024218.

(7) Spek, A. L. Structure Validation in Chemical Crystallography. *Acta Cryst D* **2009**, *65* (2), 148–155. https://doi.org/10.1107/S090744490804362X.

(8) Sauerbrey, G. Verwendung von Schwingquarzen zur Wägung dünner Schichten und zur Mikrowägung. *Z. Physik* **1959**, *155* (2), 206–222. https://doi.org/10.1007/BF01337937.

(9) Chang Chien, T.-C.; Delley, M. F. Disentangling Chemical Interaction and Electric Fields at Electrochemical Interfaces. *J. Phys. Chem. C* **2024**, *129* (1), 999–1012. https://doi.org/10.1021/acs.jpcc.4c07553.

(10) Kühne, T. D.; Iannuzzi, M.; Del Ben, M.; Rybkin, V. V.; Seewald, P.; Stein, F.; Laino, T.; Khaliullin, R. Z.; Schütt, O.; Schiffmann, F.; Golze, D.; Wilhelm, J.; Chulkov, S.; Bani-Hashemian, M. H.; Weber, V.; Borštnik, U.; Taillefumier, M.; Jakobovits, A. S.; Lazzaro, A.; Pabst, H.; Müller, T.; Schade, R.; Guidon, M.; Andermatt, S.; Holmberg, N.; Schenter, G. K.; Hehn, A.; Bussy, A.; Belleflamme, F.; Tabacchi, G.; Glöß, A.; Lass, M.; Bethune, I.; Mundy, C. J.; Plessl, C.; Watkins, M.; VandeVondele, J.; Krack, M.; Hutter, J. CP2K: An Electronic Structure and Molecular Dynamics Software Package - Quickstep: Efficient and Accurate Electronic Structure Calculations. *J. Chem. Phys.* **2020**, *152* (19), 194103. https://doi.org/10.1063/5.0007045.

(11) Hohenberg, P.; Kohn, W. Inhomogeneous Electron Gas. *Phys. Rev.* **1964**, *136* (3B), B864–B871. https://doi.org/10.1103/PhysRev.136.B864.

(12) Kohn, W.; Sham, L. J. Self-Consistent Equations Including Exchange and Correlation Effects. *Phys. Rev.* **1965**, *140* (4A), A1133–A1138. https://doi.org/10.1103/PhysRev.140.A1133.

(13) Goedecker, S.; Teter, M.; Hutter, J. Separable Dual-Space Gaussian Pseudopotentials. *Phys. Rev. B* **1996**, *54* (3), 1703–1710. https://doi.org/10.1103/PhysRevB.54.1703.

(14) Perdew, J. P.; Burke, K.; Ernzerhof, M. Generalized Gradient Approximation Made Simple. *Phys. Rev. Lett.* **1996**, *77* (18), 3865–3868. https://doi.org/10.1103/PhysRevLett.77.3865.

(15) Sabatini, R.; Gorni, T.; de Gironcoli, S. Nonlocal van Der Waals Density Functional Made Simple and Efficient. *Phys. Rev. B* **2013**, *87* (4), 041108. https://doi.org/10.1103/PhysRevB.87.041108.

(16) Fernández-Terán, R.; Sévery, L. Living Long and Prosperous: Productive Intraligand Charge-Transfer States from a Rhenium(I) Terpyridine Photosensitizer with Enhanced Light Absorption. *Inorg. Chem.* **2021**, *60* (3), 1334–1343. https://doi.org/10.1021/acs.inorgchem.0c01939.

(17) Schneider, H.-J.; Hacket, F.; Rüdiger, V.; Ikeda, H. NMR Studies of Cyclodextrins and Cyclodextrin Complexes. *Chem. Rev.* **1998**, *98* (5), 1755–1786. https://doi.org/10.1021/cr970019t.

(18) Hibbert, D. B.; Thordarson, P. The Death of the Job Plot, Transparency, Open Science and Online Tools, Uncertainty Estimation Methods and Other Developments in Supramolecular Chemistry Data Analysis. *Chem. Commun.* **2016**, *52* (87), 12792–12805. https://doi.org/10.1039/C6CC03888C.

(19) Thordarson, P. Determining Association Constants from Titration Experiments in Supramolecular Chemistry. *Chem. Soc. Rev.* **2011**, *40* (3), 1305–1323. https://doi.org/10.1039/C0CS00062K.

(20) Thordarson, P. *BindFit v0.5 | Supramolecular*. http://app.supramolecular.org/bindfit/ (accessed 2025-03-26).

(21) Woutersen, S.; Mu, Y.; Stock, G.; Hamm, P. Hydrogen-Bond Lifetime Measured by Time-Resolved 2D-IR Spectroscopy: *N*-Methylacetamide in Methanol. *Chemical Physics* **2001**, *266* (2), 137–147. https://doi.org/10.1016/S0301-0104(01)00224-5.

(22) Farmer, A. L.; Procacci, B.; Shaw, D. J.; Gurung, S.; Fairlamb, I. J. S.; Lynam, J. M.; Hunt, N. T. Ultrafast Vibrational Spectroscopic Analysis of the Ubiquitous Precatalyst [Mn2(CO)10] in Different Solvents. *The Journal of Chemical Physics* **2025**, *162* (17), 174302. https://doi.org/10.1063/5.0254482.

(23) Dub, P. A.; Filippov, O. A.; Belkova, N. V.; Daran, J.-C.; Epstein, L. M.; Poli, R.; Shubina, E. S. Hydrogen Bonding to Carbonyl Hydride Complex Cp*Mo(PMe3)2(CO)H and Its Role in Proton Transfer. *Dalton Trans.* **2010**, *39* (8), 2008–2015. https://doi.org/10.1039/B916084A.

(24) Hamley, P. A.; Kazarian, S. G.; Poliakoff, M. Hydrogen-Bonding and Photochemistry of Organometallics in Liquid Xenon Solution in the Presence of Proton Donors: A Low Temperature Infrared Study of the Interaction of (CF3)3COH with (C5Me5)M(CO)2L (M = Mn and Re; L = CO, N2, and H2) and with (C5Me5)V(CO)4. *Organometallics* **1994**, *13* (5), 1767–1774. https://doi.org/10.1021/om00017a038.

(25) Reek, J. N. H.; de Bruin, B.; Pullen, S.; Mooibroek, T. J.; Kluwer, A. M.; Caumes, X. Transition Metal Catalysis Controlled by Hydrogen Bonding in the Second Coordination Sphere. *Chem. Rev.* **2022**, *122* (14), 12308–12369. https://doi.org/10.1021/acs.chemrev.1c00862.

(26) Reinsberg, P. H.; Baltruschat, H. Potential- and Cation-Dependent Adsorption of Acetonitrile on Gold Investigated via Surface Enhanced Infrared Absorption Spectroscopy. *Electrochimica Acta* **2020**, *334,* 135609. https://doi.org/10.1016/j.electacta.2019.135609.

(27) Bard, A.; Rondon, R.; Marquez, D. T.; Lanterna, A. E.; Scaiano, J. C. How Fast Can Thiols Bind to the Gold Nanoparticle Surface? *Photochem & Photobiology* **2018**, *94* (6), 1109–1115. https://doi.org/10.1111/php.13010.

(28) DeVetter, B. M.; Mukherjee, P.; Murphy, C. J.; Bhargava, R. Measuring Binding Kinetics of Aromatic Thiolated Molecules with Nanoparticles via Surface-Enhanced Raman Spectroscopy. *Nanoscale* **2015**, *7* (19), 8766–8775. https://doi.org/10.1039/C5NR01006C.
(29) Yin, W.-J.; Krack, M.; Li, X.; Chen, L.-Z.; Liu, L.-M. Periodic Continuum Solvation Model Integrated with First-Principles Calculations for Solid Surfaces. *Progress in Natural Science: Materials International* **2017**, *27* (2), 283–288. https://doi.org/10.1016/j.pnsc.2017.03.003.
(30) Andreussi, O.; Dabo, I.; Marzari, N. Revised Self-Consistent Continuum Solvation in Electronic-Structure Calculations. *The Journal of Chemical Physics* **2012**, *136* (6), 064102. https://doi.org/10.1063/1.3676407.
(31) Martínez, L.; Andrade, R.; Birgin, E. G.; Martínez, J. M. PACKMOL: A package for building initial configurations for molecular dynamics simulations. *Journal of Computational Chemistry* **2009**, *30* (13), 2157–2164. https://doi.org/10.1002/jcc.21224.
(32) Humphrey, W.; Dalke, A.; Schulten, K. VMD: Visual Molecular Dynamics. *Journal of Molecular Graphics* **1996**, *14* (1), 33–38. https://doi.org/10.1016/0263-7855(96)00018-5.
(33) Stone, J. E.; Gullingsrud, J.; Grayson, P.; Schulten, K. A System for Interactive Molecular Dynamics Simulation. In *Proceedings of the 2001 symposium on Interactive 3D graphics*; ACM, 2001; pp 191–194. https://doi.org/10.1145/364338.364398.
(34) Eargle, J.; Wright, D.; Luthey-Schulten, Z. Multiple Alignment of Protein Structures and Sequences for VMD. *Bioinformatics* **2006**, *22* (4), 504–506. https://doi.org/10.1093/bioinformatics/bti825.
(35) Frishman, D.; Argos, P. Knowledge-Based Protein Secondary Structure Assignment. *Proteins: Structure, Function, and Bioinformatics* **1995**, *23* (4), 566–579. https://doi.org/10.1002/prot.340230412.
(36) Varshney, A.; Brooks, F. P.; Wright, W. V. Computing Smooth Molecular Surfaces. *IEEE Computer Graphics and Applications* **1994**, *14* (5), 19–25. https://doi.org/10.1109/38.310720.
(37) Sanner, M. F.; Olson, A. J.; Spehner, J.-C. Fast and Robust Computation of Molecular Surfaces. In *Proceedings of the eleventh annual symposium on Computational geometry - SCG '95*; ACM, 1995; pp 406–407. https://doi.org/10.1145/220279.220324.
(38) Sharma, R.; Zeller, M.; Pavlovic, V. I.; Huang, T. S.; Lo, Z.; Chu, S.; Zhao, Y.; Phillips, J. C.; Schulten, K. Speech/Gesture Interface to a Visual-Computing Environment. *IEEE Computer Graphics and Applications* **2000**, *20* (2), 29–37. https://doi.org/10.1109/38.824531.
